# Sex differences in clinically diagnosed psychiatric disorders over the lifespan: a nationwide register-based study in Sweden

**DOI:** 10.1016/j.lanepe.2024.101105

**Published:** 2024-10-21

**Authors:** Yihui Yang, Fang Fang, Filip K. Arnberg, Ralf Kuja-Halkola, Brian M. D'Onofrio, Henrik Larsson, Isabell Brikell, Zheng Chang, Ole A. Andreassen, Paul Lichtenstein, Unnur A. Valdimarsdóttir, Donghao Lu

**Affiliations:** a Institute of Environmental Medicine, Karolinska Institutet, Stockholm, Sweden; b National Centre for Disaster Psychiatry, Department of Medical Sciences, Uppsala University, Uppsala, Sweden; c Department of Medical Epidemiology and Biostatistics, Karolinska Institutet, Stockholm, Sweden; d Department of Psychological and Brain Sciences, Indiana University, Bloomington, USA; e School of Medical Sciences, Örebro University, Örebro, Sweden; f Department of Global Public Health and Primary Care, University of Bergen, Bergen, Norway; g Department of Biomedicine, Aarhus University, Aarhus, Denmark; h NORMENT Centre, Institute of Clinical Medicine, University of Oslo, Oslo, Norway; i Division of Mental Health and Addiction, Oslo University Hospital, Oslo, Norway; j Center of Public Health Sciences, Faculty of Medicine, University of Iceland, Reykjavik, Iceland; k Department of Epidemiology, Harvard T.H. Chan School of Public Health, Boston, MA, USA

**Keywords:** Sex difference, Psychiatric disorder, Women's mental health, Lifespan

## Abstract

**Background:**

Limited studies exist on sex differences in incidence rates of psychiatric disorders across the lifespan. This study aims to analyze sex differences in the incidence rates of clinically diagnosed psychiatric disorders over the lifespan.

**Methods:**

We conducted a nationwide register-based cohort study, including all individuals who were born in Sweden and lived in Sweden between 2003 and 2019, including 4,818,071 females and 4,837,829 males. We calculated sex- and age-specific standardized incidence rates for any and 10 major types of psychiatric disorders. Multivariable-adjusted incidence rate differences (IRDs) for diagnosed psychiatric disorders between females and males were estimated.

**Findings:**

During a follow-up of 119,420,908 person-years, males showed a higher incidence rate of any diagnosed psychiatric disorder than females at age 5–9 (IRD = −8.93; 95% CI: −9.08 to −8.79; per 1000 person-years), whereas females showed a higher rate than males at age 15–19 (IRD = 9.33; 95% CI: 9.12–9.54) and onwards (except age 60–69). Specifically, among females, excess rates were apparent for depressive, anxiety, eating, stress-related and bipolar disorders at age 10–54, whereas among males, excess rates were pronounced for autism and attention deficit hyperactivity disorders before age 14, drug use disorders at age 15–54, and alcohol use disorders in adulthood. For schizophrenia, the male excess at age 15–49 shifted to female excess at age 60–79. The magnitude of IRDs were greater in recent years and individuals with lower socioeconomic status.

**Interpretation:**

Knowledge about the lifespan and socioeconomic variations in the sex differences in rates of diagnosed psychiatric disorders may inform targeted screening/intervention strategies.

**Funding:**

Vetenskapsrådet, FORTE, Karolinska Institutet Strategic Research Area in Epidemiology and Biostatistics, and Icelandic Research Fund.

**Research in context**

**Evidence before this study**

Psychiatric disorders are pressing public health concerns. Although available evidence points towards substantial sex differences, it remains challenging to reduce the gap in mental health called by the World Health Organization. Previous evidence were mainly based on prevalence studies, without differentiating incident cases from prevalent and recurrent cases. In contrast, a longitudinal design assessing new-onset, or incident cases, of psychiatric disorders over the lifespan may inform optimal time windows for screening and interventions, with the ultimate goal of reducing the gap in mental health.

To identify studies which assessed sex differences in incidence rates of major psychiatric disorders, we searched literature in PubMed using the search terms in title or abstract (((Sex difference∗ [Title/Abstract]) OR (gender difference∗[Title/Abstract])) AND ((psychiatric disorder∗ [Title/Abstract]) OR (depress∗ [Title/Abstract]) OR (anxiety [Title/Abstract]) OR (bipolar [Title/Abstract]) OR (post-traumatic stress disorder [Title/Abstract]) OR (eating disorder∗ [Title/Abstract]) OR (attention deficit hyperactivity disorder∗ [Title/Abstract]) OR (autism [Title/Abstract]) OR (drug use disorder∗ [Title/Abstract]) OR (alcohol use disorder∗ [Title/Abstract]) OR (schizophrenia [Title/Abstract]))) AND (incidence [Title/Abstract]). We did not put restrictions on language or year of publication. We identified 555 literature. A total of 31 studies investigated a single psychiatric disorder or only presented sex-specific incidence rates of psychiatric disorders. Few studies used a sex/gender perspective, by specifically investigating magnitude of sex differences in incidence rates of psychiatric disorders. In addition, a life-course approach is important to study the time course of sex differences, to better guide gendered prevention or intervention strategies at different life stages. Two studies investigated sex differences in depression and schizophrenia over the lifespan, whereas the magnitude of difference has not been estimated in terms of incidence rate.

**Added value of this study**

In this nationwide register-based study, we found pronounced sex differences in the incidence rates of diagnosed psychiatric disorders over the lifespan, which varied depending on age, type of psychiatric disorders, calendar period, and socioeconomic status.

Our study significantly advanced the current knowledge on sex differences in psychiatric disorders. Specifically, we illustrated excess incidence rates among females with an onset in adolescence in stress-related, eating, and bipolar disorders and a bimodal excess incidence rate among females across lifespan in depressive disorders. We also showed excess incidence rates in early life among males with a sex-convergence in adult life for attention deficit hyperactivity disorder (ADHD) and excess incidence rates among males from age 20 onwards in alcohol use disorders. Second, we revealed that the incidence rate differences (IRDs) in all psychiatric disorders were enlarging over calendar period in Sweden, except for alcohol use disorders and schizophrenia. Third, we demonstrated greater IRDs in most psychiatric disorders among individuals with lower socioeconomic status.

**Implications of all the available evidence**

In the context of increasing sex differences in psychiatric disorders over time, it is urgent to reduce the sex differences in psychiatric disorders. Our findings that sex differences in psychiatric disorders exist almost across the whole life emphasized the adoption of gendered mental health prevention strategies and consistent risk factor management over the lifecourse. The variation of such differences across age and socioeconomic status suggests that the current knowledge - females predominate in internalizing disorders and males predominate in externalizing disorders - can be improved by incorporating information on age and socioeconomic status. Our study also provides evidence for screening and intervention strategies to target specific age groups and socially disadvantaged populations who experience significant sex disparity in psychiatric disorders.

## Introduction

Psychiatric disorders are among the most pressing public health concerns, causing 125 million disability-adjusted life-years globally in 2019,^1^ and the Coronavirus disease 2019 (COVID-19) pandemic has increased the burden of psychiatric disorders since then.^2^ Available evidence points towards substantial sex differences in susceptibility, chronicity, and symptomatology of psychiatric disorders.^3^ For example, females have a higher prevalence of internalizing disorders than males, including mood disorders and anxiety, whereas males predominate in externalizing disorders, like substance use disorders.^4^

Although sex differences in psychiatric disorders are well documented, it remains challenging to reduce the gap in mental health called by the World Health Organization.^5^^,^^6^ The current evidence on sex differences in psychiatric disorders is mainly based on prevalence studies,^7^, ^8^, ^9^ without differentiating incident cases from prevalent and recurrent cases. In contrast, a longitudinal design assessing new-onset, or incident cases, of psychiatric disorders over lifespan may inform optimal time windows for screening and interventions, with the ultimate goal of reducing the gap in mental health. However, among studies which were based on incidence rates of psychiatric disorders, most investigated a single psychiatric disorder,^10^, ^11^, ^12^ or presented incidence rates of psychiatric disorders among females and males separately.^13^, ^14^, ^15^, ^16^, ^17^, ^18^, ^19^ Few studies have specifically studied magnitude of sex differences in incidence rates for psychiatric disorders,^20^ which can provide direct information for gendered policies.

Age is an important effect modifier of sex differences in psychiatric disorders, as shown by age-dependent sex ratio in psychiatric disorders.^21^^,^^22^ It is important to apply a life-course approach to study the time course of sex differences, to better guide gendered prevention or intervention strategies at different life stages.^23^ Although sex differences in some common psychiatric disorders, e.g., depression, anxiety, are known to surface in adolescence, such knowledge was based on only a few studies,^18^^,^^24^^,^^25^ and the magnitude and direction of sex differences beyond adolescence was largely unknown. Only few studies investigated sex differences in depression and schizophrenia over the lifespan,^26^^,^^27^ whereas the magnitude of difference has not been estimated in terms of incidence rate.

Socioeconomic status is another important modifier of sex differences in psychiatric disorders.^26^^,^^28^ Knowing socioeconomic variation of sex differences can inform actionable prevention or intervention strategies. Although lower socioeconomic status is associated with higher risk of most psychiatric disorders,^29^ it remains unclear whether socially disadvantaged population faces greater sex differences.

Leveraging high-quality national registers in Sweden, we aimed to depict a comprehensive atlas of sex differences in the incidence rates of clinically diagnosed psychiatric disorders over the lifespan, focusing on analyzing such differences by calendar period, socioeconomic status, and different types of psychiatric disorders.

## Methods

### Study population and ascertainment of psychiatric disorders

Individuals who live in Sweden are entitled to high-cost-protection in healthcare, including mental health service, which ensures universal tax-funded healthcare access for everyone in Sweden. We conducted a nationwide register-based cohort study based on the Swedish Total Population Register (TPR), including 13,857,034 individuals born in Sweden during 1903–2019. Using the unique personal identification number, we linked the TPR to other national registers, including the National Patient, Migration, and Causes of Death Registers. All individuals aged less than 100 years and lived in Sweden during 2003–2019 were included in our study and were followed from birth or January 1st, 2003, whichever came later, until emigration, death, the 100th birthday, or December 31st, 2019, whichever occurred first. The year 2003 was chosen as the start of the follow-up because the registration of outpatient records started from 2001, and a two-year wash-out period was added to ensure the identification of incident cases of psychiatric disorder. We excluded individuals who emigrated (n = 402,312), died (n = 3,548,691), or turned age 100 (n = 250,131) before cohort entry, leaving a total of 9,655,900 individuals (4,818,071 females and 4,837,829 males) in the analysis.

We obtained information on legally registered sex from the TPR. In Sweden, sex is assigned at birth, except for a small group of people who medically and legally changed gender later in life.^30^ We identified diagnoses of psychiatric disorders from the National Patient Register (NPR), which covers all admissions to inpatient care since 1987 and > 80% visits to outpatient specialist care since 2001. Sweden used the International Classification of Diseases, Eighth Revision (ICD-8) from 1969 to 1986; ICD, Ninth Revision (ICD-9) from 1987 to 1996; and ICD, Tenth Revision (ICD-10) from 1997 onwards. We used the Swedish revision of the ICD codes to identify diagnoses of psychiatric disorders (ICD-8,9,10 was used to identify history of psychiatric disorders and ICD-10 was used to identify psychiatric disorder cases). In addition to any psychiatric disorder, we further studied 10 major categories of psychiatric disorders, including depressive disorders, anxiety disorders, stress-related disorders, eating disorders, attention deficit hyperactivity disorder (ADHD), autism spectrum disorder, alcohol and drug use disorders, bipolar disorder, and schizophrenia (ICD codes are summarized in Table S1). Most of these diagnoses have a high validity in the NPR.^31^, ^32^, ^33^, ^34^ Individuals with a diagnosed psychiatric disorder (i.e., any psychiatric disorder or a major category of psychiatric disorders) before cohort entry were excluded. Number of individuals, number of incident cases, and years of follow-up for the different analyses of any or major categories of psychiatric disorders are summarized in Table S2.

This study is approved by the Swedish Ethical Review Authority (2020-06540). Informed consent is waived for register-based studies in Sweden.

### Covariates

Information on all covariates was obtained annually during the follow-up. The Longitudinal Integration Database for Health Insurance and Labor Market (LISA) contains information on socioeconomic status for all Swedish residents aged ≥16 years from 1990 onward.^35^ From LISA, we collected data on household income and educational level for individuals ≥20 years. In contrast, we collected data on the highest parental income and educational attainment for individuals at age 20 or below.^36^ In addition, we collected data on civil status and region of residence from LISA. The latter was used to classify urbanization level of residence.

### Statistical analysis

First, we calculated sex- and age-specific incidence rates for any psychiatric disorder as well as different categories of psychiatric disorders, as the number of newly diagnosed cases divided by time at follow-up (person-years). The standardized incidence rate (SIR) was subsequently calculated via the method of direct standardization, using the distribution of calendar period of the accumulated person-years during follow-up as the standard.

Next, to shed light on differences in absolute risk, we estimated incidence rate differences (IRDs) and 95% confidence interval (CI) of any psychiatric disorder as well as different categories of psychiatric disorders, using adjusted Poisson regression, with a log link function. Specifically, we predicted number of cases among males and females separately, in 5-year age groups, using Poisson regression adjusted for age (as a continuous variable) and calendar period (grouped as every 4 years from 2003 to 2015–2019), and used delta-method to calculate standard errors. Then, we divided the differences in number of predicated cases, comparing females to males, by person-years among the reference group, i.e., males.^37^ To examine the temporal change in absolute differences between sexes, we calculated the IRDs for every four calendar years from 2003 to 2015–2019. To identify high-risk populations, we separately calculated the IRDs by proxies of socioeconomic status, including household income, educational level, civil status, and region of residence. Due to a very low missing rate in educational level, household income, civil status, region of residence and urbanization level, we used complete-case analysis in the stratified analyses by these indicators.

Finally, as utilization of healthcare services differs between sexes,^38^ the sex differences identified in the main analysis might be partially due to differential healthcare seeking behaviors between females and males. In a sensitivity analysis, we assessed IRDs for psychiatric disorders as the underlying reason for hospitalization, assuming that psychiatric disorders requiring inpatient care are more severe and therefore less affected by care-seeking behavior. We also calculated IRDs by urbanization level of residence, which serves a proxy of access to healthcare.

Although pharmacotherapy is a common treatment for psychiatric disorders, and relevant data are available in registers, we did not use such information as many psychotropic medications are prescribed as treatment across different psychiatric disorder subtypes and even for somatic conditions. Such analyses may therefore not correctly inform sex differences in type-specific psychiatric disorder rates.

Data management and analysis were performed using SAS, version 9.4 (SAS institute, Cary, NC) and Stata 17.0 (STATA, College Station, TX), respectively. R version 4.3.1 was used to visualize the data. Given that 10 disorder subtypes were tested, Bonferroni adjustment was applied by considering P < 0.005 as statistical significance. Because P values were difficult to visualize in figures, we corrected CIs accordingly (i.e., 99.5% CI) to have the consistent interpretation with the overall psychiatric disorder.

### Role of the funding source

The funders had no role in study design, data collection, data analysis, interpretation, or writing of the report.

## Results

During a follow-up of 119,420,908 person-years, we identified 604,271 (10.2 per 1000 person-years) first diagnoses of any psychiatric disorder among females and 549,851 (9.1 per 1000 person-years) among males (Table S2). The median (IQR) age at entry is 30 (6–54) years. Compared to males, females were older, more likely to have a lower income but a higher educational level, and non-cohabitating (Table 1).

**Table 1:** Characteristics of the study participants: a nationwide cohort study in Sweden, 2003–2019.

Characteristics	Females (N = 4,607,668)		Males (N = 4,628,707)	
	PY	%	PY	%
Total	59,293,572	100.0	60,127,336	100.0
Age, years				
0–9	8,355,499	14.1	8,731,282	14.5
10–19	7,499,759	12.6	7,752,650	12.9
20–29	6,585,684	11.1	7,289,131	12.1
30–39	6,391,460	10.8	7,093,478	11.8
40–49	7,000,781	11.8	7,547,819	12.6
50–59	7,084,609	11.9	7,372,894	12.3
60–69	6,927,492	11.7	6,957,425	11.6
70–79	5,222,662	8.8	4,711,685	7.8
80–89	3,364,886	5.7	2,297,950	3.8
90–100	860,741	1.5	373,021	0.6
Calendar period				
2003–2006	14,545,469	24.5	14,496,290	24.1
2007–2010	14,149,913	23.9	14,277,679	23.7
2011–2014	13,797,803	23.3	14,056,328	23.4
2015–2019	16,800,388	28.3	17,297,039	28.8
Educational level^a^				
Primary	8,797,073	14.8	9,403,395	15.6
Secondary	25,178,282	42.5	27,567,840	45.8
Post-secondary	24,014,438	40.5	21,801,445	36.3
Unknown	1,303,781	2.2	1,354,656	2.3
Household income^a^				
Q1	8,908,297	15.0	7,167,757	11.9
Q2	12,738,810	21.5	10,829,782	18.0
Q3	13,164,321	22.2	13,254,221	22.0
Q4	12,176,107	20.5	13,771,434	22.9
Q5	11,283,923	19.0	14,010,507	23.3
Unknown	1,022,116	1.7	1,093,635	1.8
Civil status^b^				
Non-cohabitating	32,093,176	54.1	30,868,719	51.3
Cohabitating	26,208,443	44.2	28,196,329	46.9
Unknown	991,955	1.7	1,062,288	1.8
Region of residence				
South of Sweden	13,681,324	23.1	13,778,650	22.9
Middle part of Sweden	33,137,682	55.9	33,485,476	55.7
North of Sweden	11,510,740	19.4	11,830,243	19.7
Unknown	963,827	1.6	1,032,967	1.7
Urbanization				
Large city	26,984,363	45.5	27,081,452	45.0
Dense region near large city	22,589,814	38.1	22,975,809	38.2
Dense region remotely located	3,908,557	6.6	4,040,400	6.7
Rural area near large city	1,442,831	2.4	1,480,813	2.5
Rural area (very) remotely located	3,404,182	5.7	3,515,894	5.8
Unknown	963,827	1.6	1,032,967	1.7

PY, person-year.

Table 1 shows information on all participants included in the analysis of any psychiatric disorder. During a follow-up of 119,420,908 person-years, individuals with complete information on educational level, household income, civil status, region of residence and urbanization level contributed to 117,430,213 (98%) person-years.

a For individuals aged less than 20 years, the highest parental income and educational attainment was used.

b For individuals aged no more than 16 years, their civil status was assigned “non-cohabitated”.

**Fig. 1:**
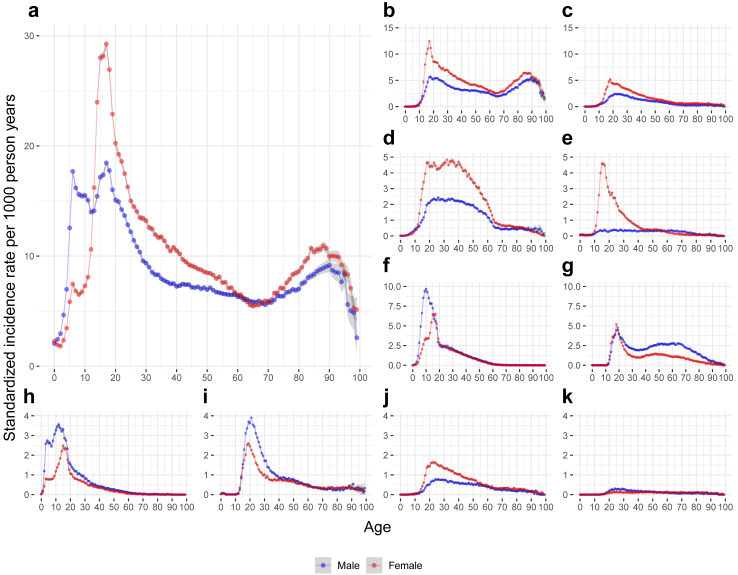
Standardized incidence rate of clinically diagnosed psychiatric disorders over the lifespan among females and males: a nationwide cohort study in Sweden, 2003–2019. This figure shows standardized incidence rate for a) any psychiatric disorder, b) depressive disorders, c) anxiety disorders, d) stress-related disorders, e) eating disorders, f) attention deficit hyperactivity disorder, g) alcohol use disorders, h) autism spectrum disorder, i) drug use disorders, j) bipolar disorder, and k) schizophrenia. Incidence rate was standardized by distribution of calendar period of the accumulated person-years during follow-up. Different scales are used for different diagnoses. The shaded bands indicate 95% CI.

Overall, males and females showed similar life-course trajectories in the SIR of clinically diagnosed psychiatric disorders. Both sexes had two peaks of SIR before adulthood (at ages 6 and 17), which are around the time when children start primary school or high school in Sweden. The SIR decreased afterward until another peak at age 80–90 (Fig. 1; Data are summarized in Table S3). For all categories of psychiatric disorders, we observed a single peak of SIR for both sexes, except for depression where a second peak was noted at age 80–90. The SIR of most disorders peaked before age 20, the SIR of bipolar disorder and schizophrenia peaked at age 20–30, whereas the SIR of stress-related disorder had a distinctly wide peak at age 20–40 (Figure S1).

**Fig. 2:**
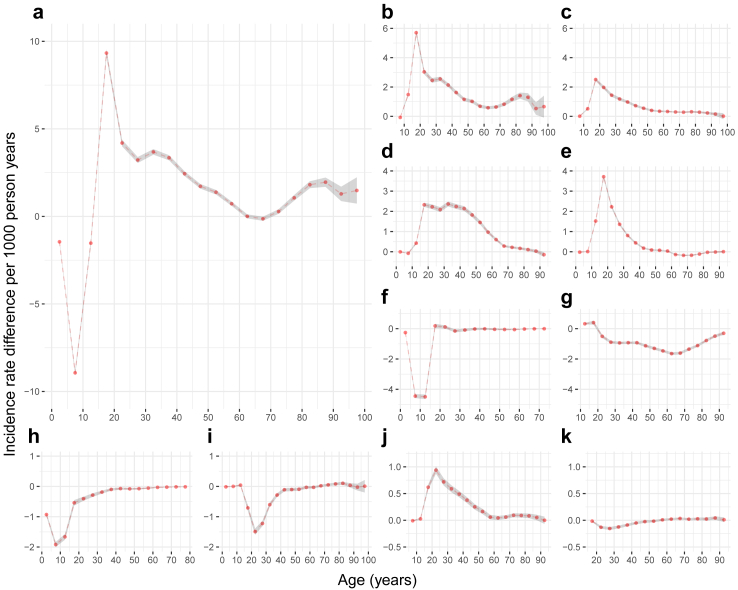
Incidence rate differences of clinically diagnosed psychiatric disorders over the lifespan in females compared to males: a nationwide cohort study in Sweden, 2003–2019. This figure shows incidence rate differences for a) any psychiatric disorder, b) depressive disorders, c) anxiety disorders, d) stress-related disorders, e) eating disorders, f) attention deficit hyperactivity disorder, g) alcohol use disorders, h) autism spectrum disorder, i) drug use disorders, j) bipolar disorder, and k) schizophrenia. The incidence rate differences were calculated for every 5-year age group and adjusted for age (as a continuous variable) and calendar year (grouped as every 4 years from 2003 to 2015–2019) at follow-up. The horizontal dashed line at 0 represents no difference in the incidence rates of females and males. An incidence rate difference above 0 indicates a higher incidence rate among females whereas an incidence rate difference below 0 indicates a higher incidence rate among males. The shaded bands indicate 95% CI for any psychiatric disorder and 99.5% CI for major types of psychiatric disorders.

Males showed a higher incidence rate of any psychiatric disorder than females before age 14, and the male excess was most pronounced at age 5–9 (IRD = −8.93; 95% CI: −9.08 to −8.79; per 1000 person-years) (Fig. 2; Table S4). However, females showed a higher incidence rate of any psychiatric disorder than males at age 15–59 and 70–99, and the female excess was most pronounced at age 15–19 (IRD = 9.33; 95% CI: 9.12–9.54). Excess incidence rates were apparent among females for depressive, anxiety, stress-related, eating, and bipolar disorders at age 10–54, whereas excess incidence rates were pronounced among males for ADHD and autism spectrum disorders before age 14, drug use disorders at age 15–54, and alcohol use disorders in adulthood. For schizophrenia, the male excess at age 15–49 shifted to a female excess at age 60–79.

**Fig. 3:**
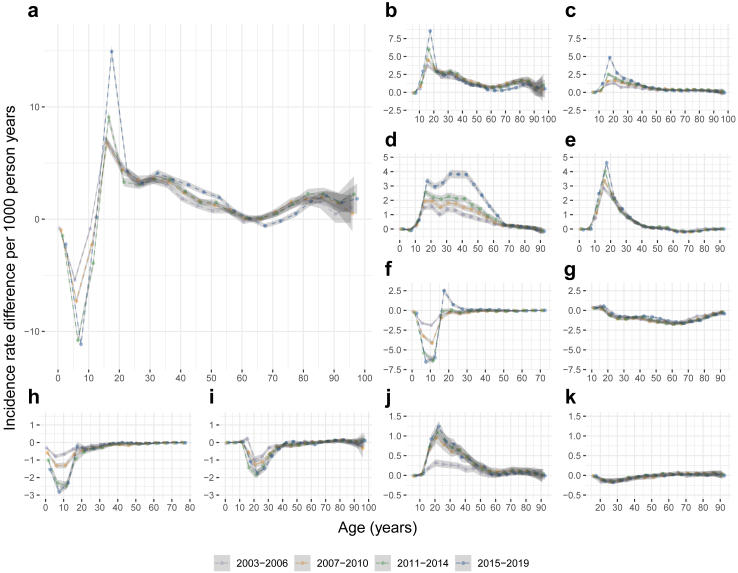
Incidence rate differences of psychiatric disorders over the lifespan in females compared to males, stratified analysis by calendar period: a nationwide cohort study in Sweden, 2003–2019. This figure shows incidence rate differences for a) any psychiatric disorder, b) depressive disorders, c) anxiety disorders, d) stress-related disorders, e) eating disorders, f) attention deficit hyperactivity disorder, g) alcohol use disorders, h) autism spectrum disorder, i) drug use disorders, j) bipolar disorder, and k) schizophrenia. The incidence rate differences were calculated for every 5-year age group and adjusted for age at follow-up (as a continuous variable). The horizontal dashed line at 0 represents no difference in the incidence rates of females and males. An incidence rate difference above 0 indicates a higher incidence rate among females whereas an incidence rate difference below 0 indicates a higher incidence rate among males. The shaded bands indicate 95% CI for any psychiatric disorder and 99.5% CI for major types of psychiatric disorders.

We found greater IRDs, both female excess and male excess, in recent calendar periods, compared to earlier calendar periods, for any psychiatric disorder (Fig. 3; Table S5). Such pattern was more pronounced before age 30. For all categories of psychiatric disorders, the IRDs appeared to increase over calendar period, at least in some age groups, except alcohol use disorders and schizophrenia.

The IRDs in any psychiatric disorder were more pronounced among individuals with a lower educational level or a lower household income, especially at younger age (Figures S2 and S3). The IRDs in stress-related disorders and alcohol use disorders were most pronounced among non-cohabitating individuals (Figure S4). Region of residence and urbanization level of residence did however not modify IRDs clearly (Figures S5 and S6). When ascertaining psychiatric disorders through inpatient care recodes alone, the sex differences attenuated to some extent (Figure S7).

## Discussion

In this nationwide cohort, we found that the life-course trajectory of the incidence rates of clinically diagnosed psychiatric disorders was similar in shape between sexes, indicating that both sexes demostrate psychiatric vulnerability at certain times during the lifespan. Despite this, there are pronounced sex differences in the incidence rates of psychiatric disorders over the lifespan, which varied depending on age, type of psychiatric disorders, calendar period, and socioeconomic status.

Our study significantly advanced the current knowledge on sex differences in psychiatric disorders. Specifically, we illustrated an excess incidence rate among females with an onset in adolescence in stress-related, eating, and bipolar disorders and a bimodal excess incidence rate in females across lifespan in depressive disorders (one at age 15–20, and another at age 80–84). We also showed an excess incidence rate among males in early life with a sex-convergence in adult life for ADHD. An excess incidence rate among males in alcohol use disorders was noted from age 20 onward, and became most pronounced at age 60–64. Second, we revealed that the IRDs in all psychiatric disorders were enlarging over calendar period in Sweden, except for alcohol use disorders and schizophrenia. Third, we demonstrated greater IRDs in most psychiatric disorders among individuals with lower socioeconomic status.

Given the complex relationship between sex and gender, the sex differences of psychiatric disorder may be results of both sex-based (e.g., genetics and gonadal hormones) and gender-based factors (e.g., gendered diagnosis, gender roles, and gender-based violence). While our study cannot differentiate sex from gender, future studies are needed to disentangle the effects of sex from gender on the dynamic differences of psychiatric disorders over lifespan.

Previous studies have shown sex differences in incidence rates of depression and anxiety emerged during adolescence.^10^^,^^14^ Using a life-course approach, our study illustrated that such differences in adolescence existed in a broader spectrum of psychiatric disorders, namely, eating, stress-related, and bipolar disorders.^14^^,^^39^ This may be attributable to an interplay between genetic factors, sex hormones, childhood adverse events, and other risk factors specific to each disorder, e.g., sex differences exist in fear physiology and body dissatisfaction,^39^^,^^40^ which are of relevance to stress-related and eating disorders. In addition, girls are perhaps better at recognizing and seeking help for these disorders, than boys.^38^ Interestingly, the sex differences in these disorders seem most striking during reproductive ages, indicating female sex hormones may be at play as well. For instance, ovarian steroid hormone fluctuations from puberty to menopause may predispose some females to the onset or worsening of psychiatric symptoms particularly during premenstrual phase, perinatal period, or menopause transition.^41^^,^^42^ Notably, for depression, there is a second peak of the IRDs at age 80–84, which has been scarcely reported. This may be partially explained by overlapping symptoms between depression and cognitive impairment, given the higher rate of cognitive decline and dementia among old females than males.^43^ More research is however warranted to understand this excess incidence rates in late-onset depression among females.

It is not surprising to find increasing IRDs over time in psychiatric disorders with excess incidence rates among females. Broadening of diagnostic criteria (especially for Post-Traumatic Stress Disorder (PTSD)),^44^ increasing coverage of NPR,^45^ as well as increasing awareness and healthcare seeking of psychiatric disorders, can contribute to increased incidence rates of these disorders. However, this alone cannot explain the greater increase in females rather than males and in some age groups instead of all ages. The increasing age at menopause^46^ may suggest a longer reproductive span in recent female populations, which may contribute to increasing IRDs over time in these disorders. For depressive, anxiety, and eating disorders, the increasing IRDs over time were most pronounced at age 15–19. Use of social media^47^ and psychological distress^48^ have increased during the recent decades among adolescents in Sweden, which may have affected girls more than boys.^40^^,^^49^ In addition, the IRDs in stress-related disorders during mid-life increased substantially from 2011–2014 to 2015–2019. Such increment echoes recent feminism campaigns and the increasing report of domestic violence among females in Sweden.^50^

The excess incidence rates among males in ADHD and autism spectrum disorders in childhood are well-documented.^14^ However, the sex differences in adult ADHD are less studied. Although the prevalence studies have shown a sex ratio close to 1:1 in adult, most of studies based on incidence rates have shown an excess incidence rates among males for ADHD in adults.^12^^,^^51^ Our data showed that IRDs in ADHD shifted from an excess incidence rate among males at age 5–9 to a slight excess incidence rate among females at age 15–24, indicating a sex convergence in adult ADHD. The catch-up effect in females might be explained by a diagnostic delay of childhood-onset ADHD among females, because ADHD symptoms are more internalizing among females than males.^52^

We observed a greater magnitude of IRDs in childhood autism spectrum disorders and ADHD over time. We have witnessed an increasing awareness and changes on diagnostic criteria of ADHD and autism during the past years.^53^ Moreover, ADHD and autism are often conceptualized as “male disorders”, which may lead to easier recognition of symtpoms and thus earlier diagnosis for boys.^54^

Drug use disorders displayed the greatest magnitude of IRDs in late adolescence, potentially because adolescent boys are more likely to engage in novelty–or risk-seeking behaviors.^55^ In contrast, although alcohol use disorders are often considered as male excess, the incidence rate was higher in girls than boys in adolescence, which is similar to the findings from other cohorts.^56^ Alcohol might have been used as a coping mechanism of negative emotions, e.g., depressive symptoms.^57^ The higher incidence rates of depression among female adolescents may thus drive the excess incidence rates among females in alcohol use disorders among adolescents. However, the female excess in alcohol use disorders shifted to male excess after age 20, and became most pronounced at age 60–64. Future studies are needed to illustrate reasons underlying the male excess in alcohol use disorders in midlife.

In line with previous studies,^58^ we found a shift from excess male incidence rates to excess female incidence rates at mid-life for schizophrenia. Although the exact mechanisms remain unknown to a great extent, sex-specific changes in gonadal hormones might have contributed to some extent.^58^ Interestingly, the IRDs in schizophrenia did not change over time. This may be partially explained by the strong genetic component of schizophrenia which is unlikely to change over time,^59^ as well as the minor changes in diagnostic criteria over the years.

Leveraging high-quality national registers in Sweden, we profiled a landscape of sex differences in the incidence rates of psychiatric disorders against an age continuum. However, several limitations should be considered. First, our findings may reflect sex differences in healthcare seeking behaviors. However, the analyses limited to psychiatric disorders ascertained through inpatient care yielded smaller yet significant IRDs. Similarly, comparable IRDs were observed across urbanization level of residence, a proxy for accessibility to specialized psychiatric care, suggesting that differential healthcare utilization between sexes cannot completely explain our findings. Second, although Sweden has universal health coverage, the incidence rates may be underestimated for those who did not seek healthcare or were attended only in primary care.^60^ That said, our findings may not generalize to milder psychiatric conditions, which are only managed in primary care. However, our findings are in line with a study showing the life-course pattern of sex difference in depressive symptoms assessed by questionnaires, indicating the underdiagnosis may not affect the pattern of sex differences (i.e., the relative manner).^26^ Third, we used the period between 1969 and 2003 as a “wash-out” to ascertain the first-ever diagnosis of psychiatric disorders. We may have misclassified some prevalent cases of psychiatric disorders as incident cases of psychiatric disorders, especially for individuals at mid- or late life. This could have led to a greater misclassification in the analysis of earlier calendar period (i.e., 2003–2006) than the analysis of 2015–2019. However, comparable patterns were observed at mid- or late life in the stratified analysis by calendar period, partly alleviating the concern. Fourth, we only included major groups of psychiatric disorders. Future studies including other types with known sex differences, e.g., personality disorders and conduct disorders,^61^^,^^62^ are needed. Fifth, to completely capture the medical history, we only included individuals who were born in Sweden. We therefore were unable to calculate the IRDs by migration status, which is an important proxy of socioeconomic status associated with psychiatric disorders.^63^ Sixth, our study focused on first diagnosis of psychiatric disorder. Future studies on sex differences in disease trajectories and comorbidities may help better understand potential sex differences in clinical courses of psychiatric disorders.^27^ Seventh, due to data unavailability, we did not identify intersex or gender diverse individuals. Future studies are needed to study the disparity in psychiatric disorders among intersex and gender diverse populations. Finally, our results may not generalize to populations with different racial or ethnic composition, social environment, and healthcare system as Sweden. However, a meta-analysis, which used data from 75 countries (including 19 in Africa, 20 in Asia, 4 in North America, 6 in South America, 24 in Europe and 2 in Oceania), showed that the odds ratio, comparing prevalence of depression among females to males, peaked at age 13–15.^26^ This is somewhat consistent with our result showing that the IRDs of depressive disorders peaked at age 15–19.

In conclusion, in the context of increasing sex differences in psychiatric disorders over time, it is urgent to reduce the sex differences in psychiatric disorders. Our findings that sex differences in psychiatric disorders exist almost across the whole life emphasized the adoption of gendered mental health prevention strategies and consistent risk factor management over the lifecourse. In addition, the significant variation of such differences across age and socioeconomic status not only suggests that the current knowledge that females predominate in internalizing disorders and males predominate in externalizing disorders may be further expanded by incorporating information on age and socioeconomic status, but also provides rationale for screening and intervention strategies to target at specific age groups and socially disadvantaged populations who experience significant sex disparity, with the ultimate goal of reducing sex differences in the mental health landscape.

## Contributors

YY, UAV, and DL conceived the study. YY and DL analyzed the data. YY drafted the manuscript. All authors interpreted the results, reviewed the manuscript and approved the decision to submit the manuscript. YY and DL accessed and verified the data. All authors were permitted to access the data upon request, and accepted the responsibility to submit the manuscript.

## Data sharing statement

The Public Access to Information and Secrecy Act in Sweden prohibits individual-level data being publicly available. Researchers who are interested in replicating this study can apply for individual-level data through Statistics Sweden (https://www.scb.se/en/services/ordering-data-and-statistics/ordering-microdata/). Data on patient health can be applied for through Socialstyrelsen (https://www.socialstyrelsen.se/en/statistics-and-data/registers/). Analysis coding can be available by request to the corresponding author.

## Declaration of interests

YY reports receiving grants from China Schaolarship Council. HL reports receiving grants from Shire Pharmaceuticals; personal fees from and serving as a speaker for Medice, Shire/Takeda Pharmaceuticals and Evolan Pharma AB; all outside the submitted work. HL is editor-in-chief of JCPP Advances. ZC reports receiving research grants from Swedish research council and Swedish research council for health, working life and welfare. OAA is a consultant to Cortechs. ai and Precision Health AS and has received speaker's honoraria form Lundbeck, Janssen, Sunovion and Otsuka outside of the submitted work. OAA receives grnats from Research Council of Norway, South-East Regional Health Authority, EEA and Norway, European Union's Horizon 2020 Research and Innovation Programme, and National Institutes of Health. UAV reports receiving grants from NordForsk, Rannis, ERC and Horizon2020. UAV reports receiving keynote lectures/honoraria from ECO (2024), EPA (2023), ISTSS (2022). DL reports receiving grants from Swedish Research Council, Swedish Research Council for Health, Working Life, and Welfare, Karolinska Institutet Strategic Research Area in Epidemiology and Biostatistics and European Research Council.
