# Supplementary material for: Sex differences in clinically diagnosed psychiatric disorders over the lifespan: a nationwide register-based study in Sweden
Source: Lancet Reg Health Eur. 2024 Oct 21;47:101105. doi: 10.1016/j.lanepe.2024.101105 (PMC11670697; doi:10.1016/j.lanepe.2024.101105)
Supplement: Appendix [file mmc1.pdf]

Supplementary appendix

Table of Contents

Figure S1. Standardized incidence rates of clinically diagnosed psychiatric disorders up to age 30 among females and males: a nationwide cohort study in Sweden, 2003-2019. 2

Figure S2. Incidence rate differences of psychiatric disorders over the lifespan in females compared to males, stratified analysis by educational level: a nationwide cohort study in Sweden, 2003-2019...3

Figure S3. Incidence rate differences of psychiatric disorders over the lifespan in females compared to males, stratified analysis by household income: a nationwide cohort study in Sweden, 2003-2019...4

Figure S4. Incidence rate differences of psychiatric disorders over the lifespan in females compared to males, stratified analysis by civil status: a nationwide cohort study in Sweden, 2003-2019...5

Figure S5. Incidence rate differences of psychiatric disorders over the lifespan in females compared to males, stratified analysis by region of residence: a nationwide cohort study in Sweden, 2003-2019...6

Figure S6. Incidence rate differences of psychiatric disorders over the lifespan in females compared to males, stratified analysis by level of urbanization: a nationwide cohort study in Sweden, 2003-2019...7

Figure S7. Incidence rate differences of psychiatric disorders requesting inpatient care over the lifespan in females compared to males: a nationwide cohort study in Sweden, 2003-2019 8

Table S1. Classification of any psychiatric disorder and major types of psychiatric disorders 9

Table S2. Number of individuals included, number of cases, follow-up time, and incidence rates of any and major types of psychiatric disorders..10

Table S3. Standardized incidence rate of clinically diagnosed psychiatric disorders over the lifespan among females and males: a nationwide cohort study in Sweden, 2003-2019.....11

Table S4. Incidence rate differences of clinically diagnosed psychiatric disorders over the lifespan in females compared to males: a nationwide cohort study in Sweden, 2003-2019. 35

Table S5. Incidence rate differences of psychiatric disorders over the lifespan in females compared to males, stratified analysis by calendar period: a nationwide cohort study in Sweden, 2003-2019...40

Figure S1. Standardized incidence rates of clinically diagnosed psychiatric disorders up to age 30 among females and males: a nationwide cohort study in Sweden, 2003-2019.

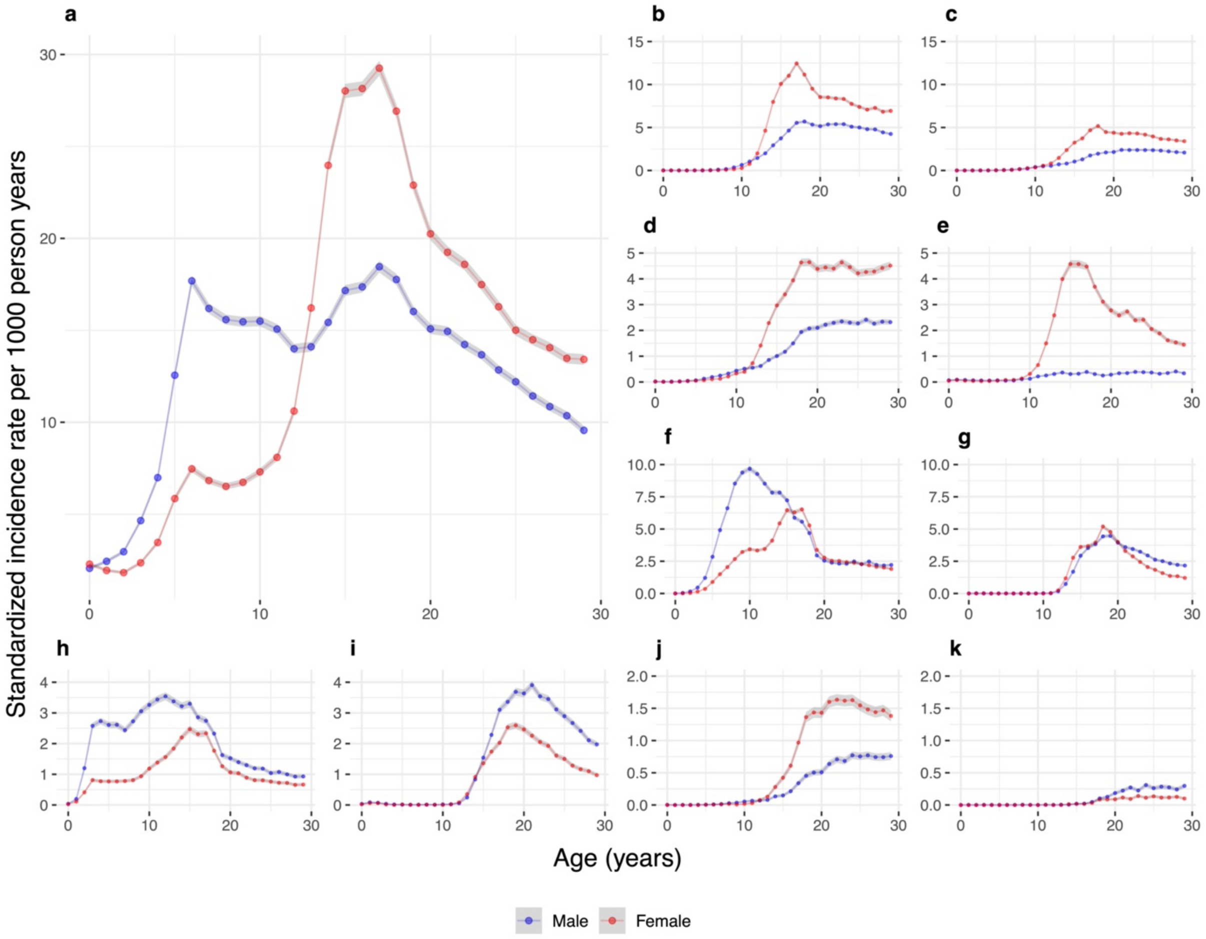

This figure shows standardized incidence rate for a) any psychiatric disorder, b) depressive disorders, c) anxiety disorders, d) stress-related disorders, e) eating disorders, f) attention deficit hyperactivity disorder, g) alcohol use disorders, h) autism spectrum disorder, i) drug use disorders, j) bipolar disorder, and k) schizophrenia.
Incidence rate was standardized by distribution of calendar period of the accumulated person-years during follow-up. Different scales are used for different diagnoses.

Figure S2. Incidence rate differences of psychiatric disorders over the lifespan in females compared to males, stratified analysis by educational level: a nationwide cohort study in Sweden, 2003-2019

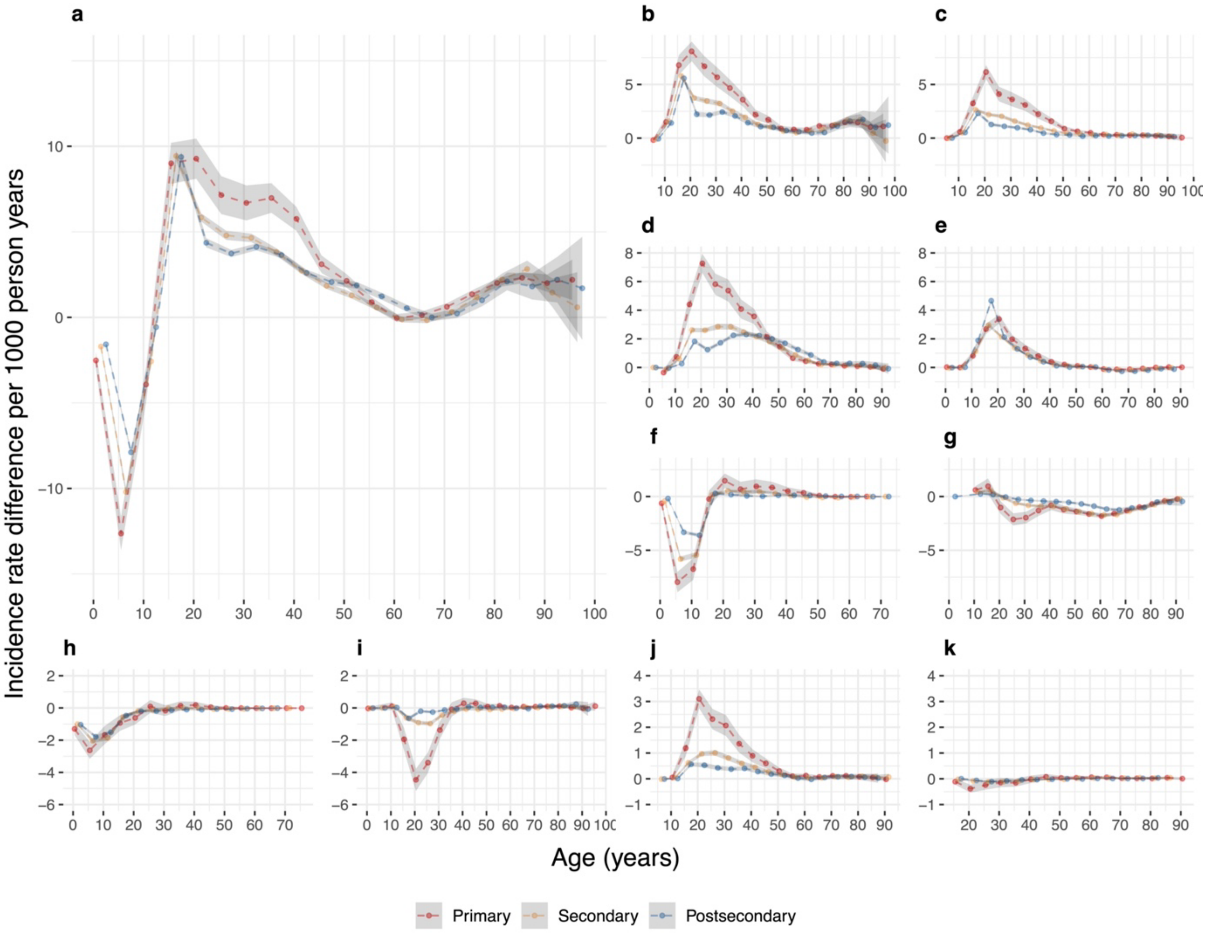

This figure shows incidence rate differences for a) any psychiatric disorder, b) depressive disorders, c) anxiety disorders, d) stress-related disorders, e) eating disorders, f) attention deficit hyperactivity disorder, g) alcohol use disorders, h) autism spectrum disorder, i) drug use disorders, j) bipolar disorder, and k) schizophrenia.
The incidence rate differences were calculated for every 5-year age group and adjusted for age (as a continuous variable) and calendar year (grouped as every 4 years from 2003 and 2015-2019) at follow-up. Please note different scales were used for different diagnoses.
The horizontal dashed line at 0 represents no difference in the incidence rates of females and males. An incidence rate difference above 0 indicates a higher incidence rate among females whereas an incidence rate difference below 0 indicates a higher incidence rate among males.

Figure S3. Incidence rate differences of psychiatric disorders over the lifespan in females compared to males, stratified analysis by household income: a nationwide cohort study in Sweden, 2003-2019

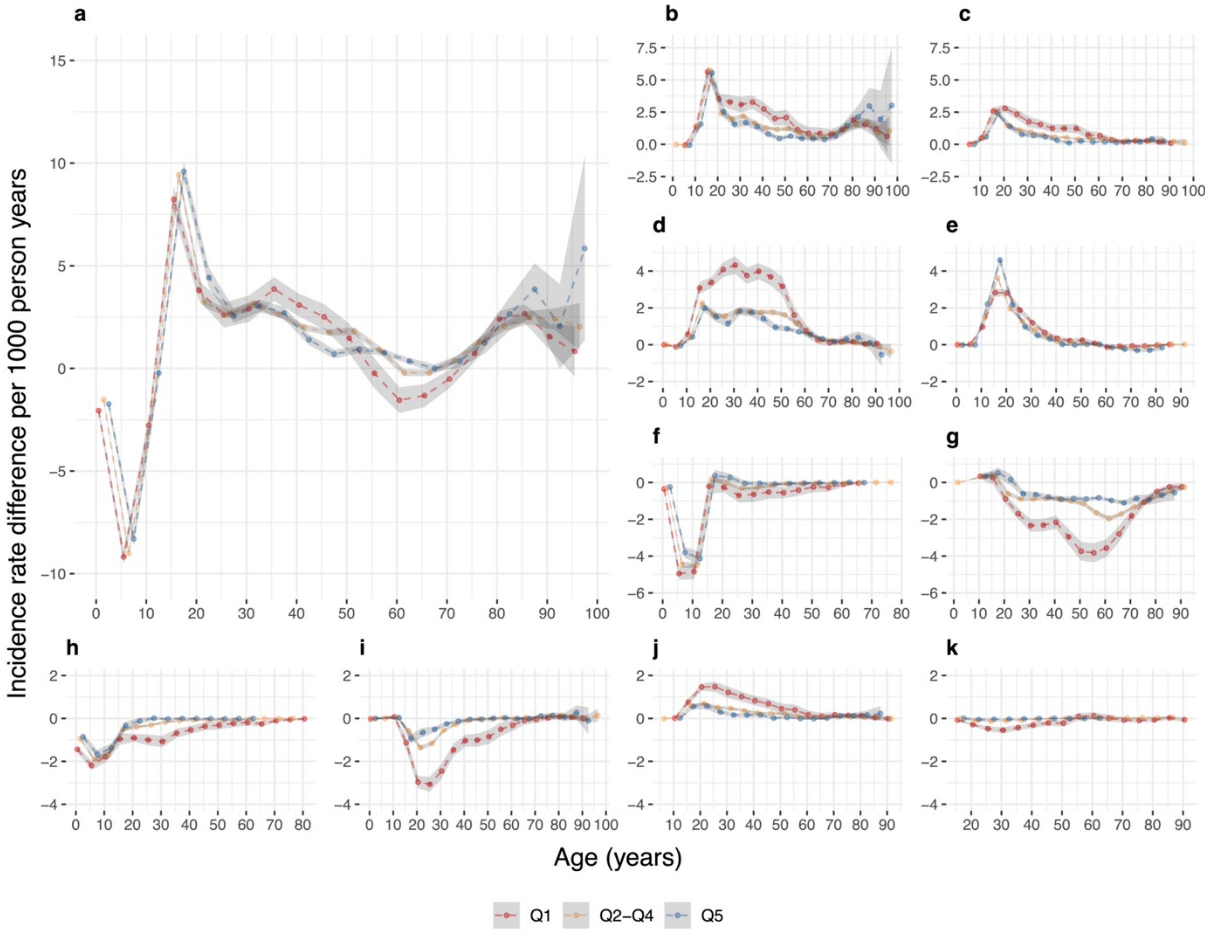

This figure shows incidence rate differences for a) any psychiatric disorder, b) depressive disorders, c) anxiety disorders, d) stress-related disorders, e) eating disorders, f) attention deficit hyperactivity disorder, g) alcohol use disorders, h) autism spectrum disorder, i) drug use disorders, j) bipolar disorder, and k) schizophrenia.
Q1, Q2, Q3, Q4 and Q5 represent 0-20%, 21-40%, 41-60%, 61-80% and 81-100% of household income by calendar year at follow-up, respectively.
The incidence rate differences were calculated for every 5-year age group and adjusted for age (as a continuous variable) and calendar year (grouped as every 4 years from 2003 and 2015-2019) at follow-up. Please note different scales were used for different diagnoses.
The horizontal dashed line at 0 represents no difference in the incidence rates of females and males. An incidence rate difference above 0 indicates a higher incidence rate among females whereas an incidence rate difference below 0 indicates a higher incidence rate among males.

Figure S4. Incidence rate differences of psychiatric disorders over the lifespan in females compared to males, stratified analysis by civil status: a nationwide cohort study in Sweden, 2003-2019

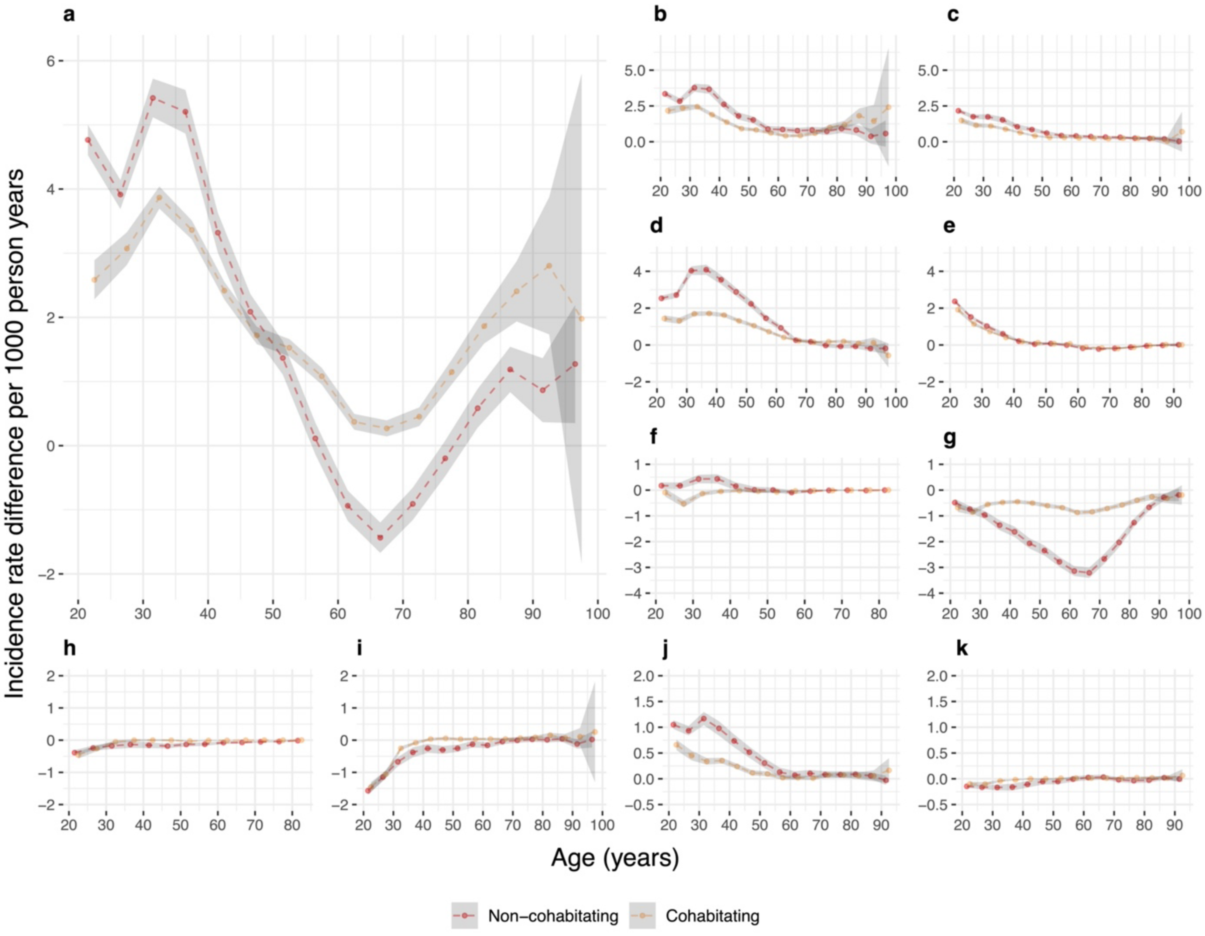

This figure shows incidence rate differences for a) any psychiatric disorder, b) depressive disorders, c) anxiety disorders, d) stress-related disorders, e) eating disorders, f) attention deficit hyperactivity disorder, g) alcohol use disorders, h) autism spectrum disorder, i) drug use disorders, j) bipolar disorder, and k) schizophrenia.

The incidence rate differences were calculated for every 5-year age group and adjusted for age (as a continuous variable) and calendar year (grouped as every 4 years from 2003 and 2015-2019) at follow-up. Please note different scales were used for different diagnoses.

The horizontal dashed line at 0 represents no difference in the incidence rates of females and males. An incidence rate difference above 0 indicates a higher incidence rate among females whereas an incidence rate difference below 0 indicates a higher incidence rate among males.

Figure S5. Incidence rate differences of psychiatric disorders over the lifespan in females compared to males, stratified analysis by region of residence: a nationwide cohort study in Sweden, 2003-2019

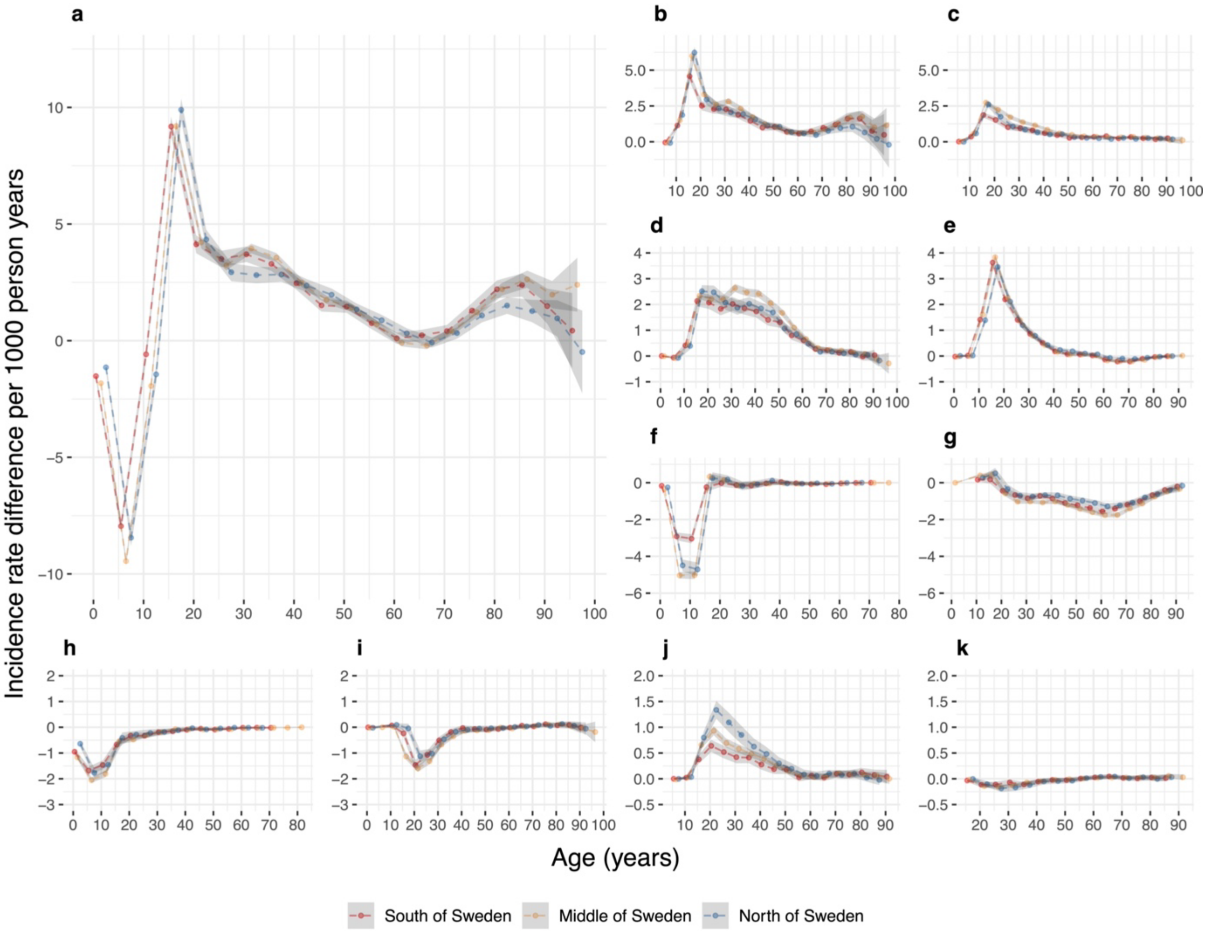

This figure shows incidence rate differences for a) any psychiatric disorder, b) depressive disorders, c) anxiety disorders, d) stress-related disorders, e) eating disorders, f) attention deficit hyperactivity disorder, g) alcohol use disorders, h) autism spectrum disorder, i) drug use disorders, j) bipolar disorder, and k) schizophrenia.
The incidence rate differences were calculated for every 5-year age group and adjusted for age (as a continuous variable) and calendar year (grouped as every 4 years from 2003 and 2015-2019) at follow-up. Please note different scales were used for different diagnoses.
The horizontal dashed line at 0 represents no difference in the incidence rates of females and males. An incidence rate difference above 0 indicates a higher incidence rate among females whereas an incidence rate difference below 0 indicates a higher incidence rate among males.

Figure S6. Incidence rate differences of psychiatric disorders over the lifespan in females compared to males, stratified analysis by level of urbanization: a nationwide cohort study in Sweden, 2003-2019

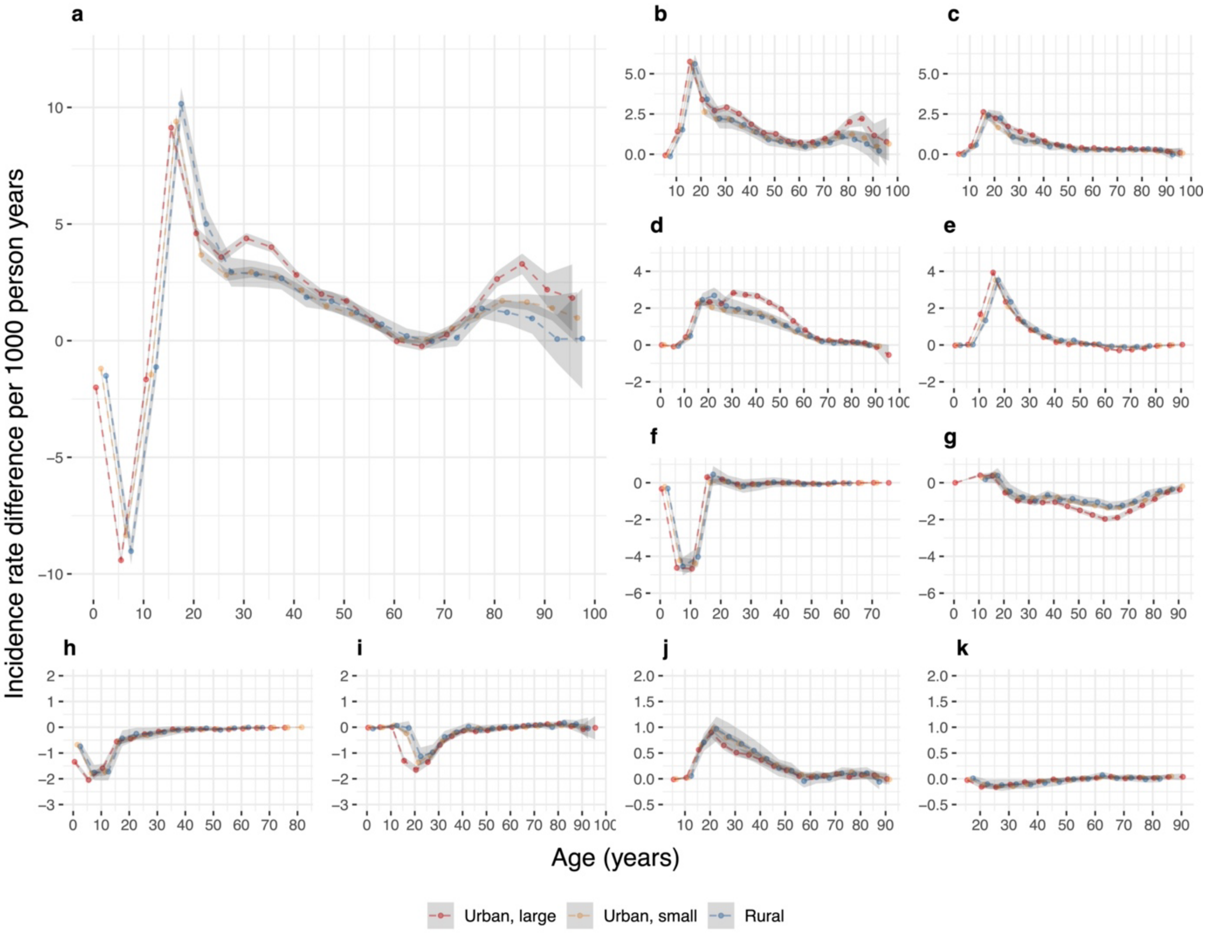

This figure shows incidence rate differences for a) any psychiatric disorder, b) depressive disorders, c) anxiety disorders, d) stress-related disorders, e) eating disorders, f) attention deficit hyperactivity disorder, g) alcohol use disorders, h) autism spectrum disorder, i) drug use disorders, j) bipolar disorder, and k) schizophrenia.

The incidence rate differences were calculated for every 5-year age group and adjusted for age (as a continuous variable) and calendar year (grouped as every 4 years from 2003 and 2015-2019) at follow-up. Please note different scales were used for different diagnoses.

The horizontal dashed line at 0 represents no difference in the incidence rates of females and males. An incidence rate difference above 0 indicates a higher incidence rate among females whereas an incidence rate difference below 0 indicates a higher incidence rate among males.

Figure S7. Incidence rate differences of psychiatric disorders requesting inpatient care over the lifespan in females compared to males: a nationwide cohort study in Sweden, 2003-2019

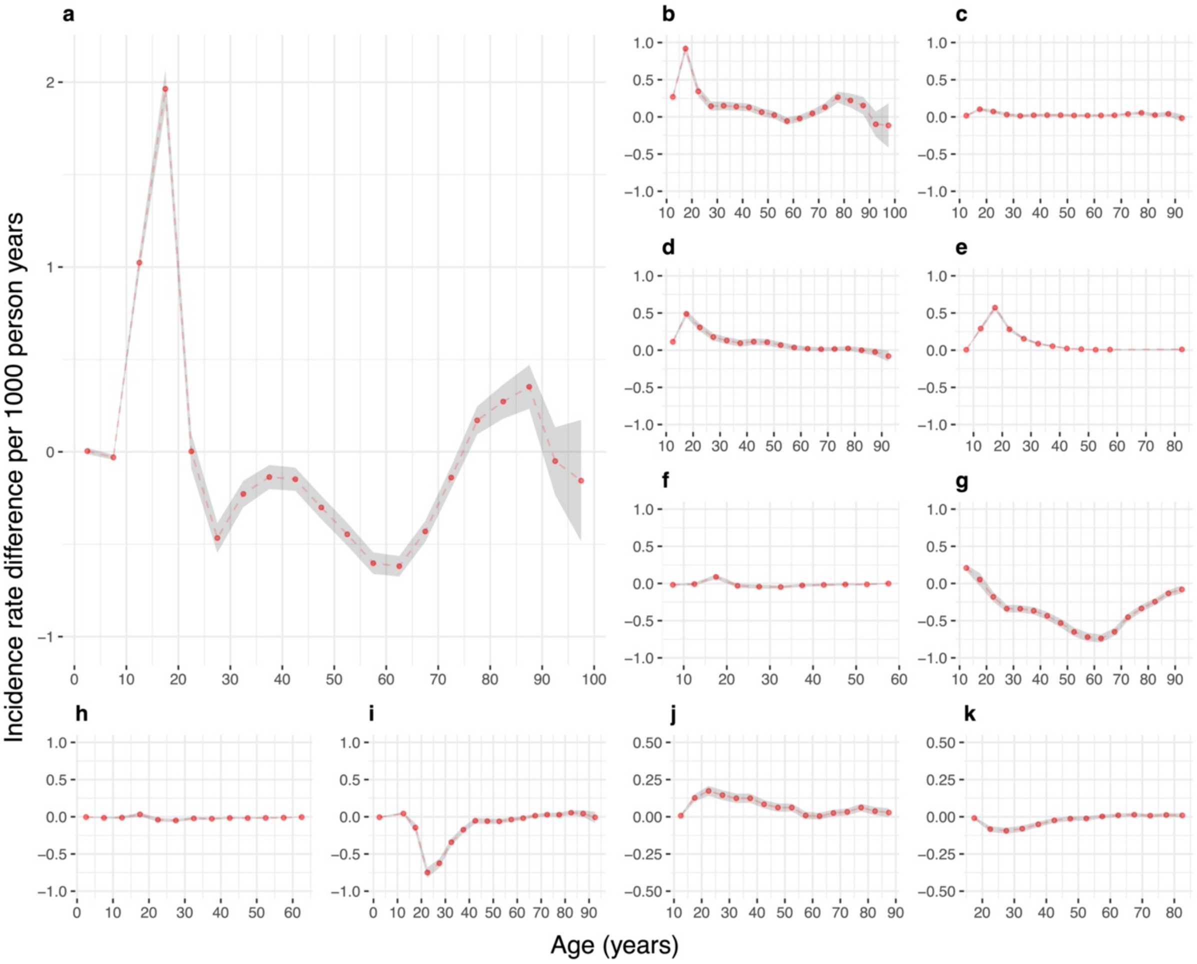

This figure shows incidence rate differences for a) any psychiatric disorder, b) depressive disorders, c) anxiety disorders, d) stress-related disorders, e) eating disorders, f) attention deficit hyperactivity disorder, g) alcohol use disorders, h) autism spectrum disorder, i) drug use disorders, j) bipolar disorder, and k) schizophrenia.
The incidence rate differences were calculated for every 5-year age group and adjusted for age (as a continuous variable) and calendar year (grouped as every 4 years from 2003 and 2015-2019) at follow-up. Please note different scales were used for different diagnoses.
The horizontal dashed line at 0 represents no difference in the incidence rates of females and males. An incidence rate difference above 0 indicates a higher incidence rate among females whereas an incidence rate difference below 0 indicates a higher incidence rate among males.

Table S1. Classification of any psychiatric disorder and major types of psychiatric disorders

	ICD 8	ICD 9	ICD 10
Any psychiatric disorder	291-315, except 3001, 3005-3009, 302, 305, 3061 3063, 3065-3069, 309	291-319, except 299 B/W/X, 300 B/F/G/H/W/X, 302, 306, 307A/D/G/H/W, 310, 315, 316	F10-F98, except F17, F80-F83 and F88-F89
Depressive disorders	3004	300E, 311	F32-F39
Anxiety disorders	3000, 3002	300A, 300C	F40.0, F40.1, F40.2, F41.0, F41.1
Stress-related disorders	307	308-309	F43
Eating disorders	-	307B, 307F	F50
ADHD	-	314	F90
Alcohol use disorders	303	303, 305A	F10
Autism spectrum disorders	-	299A	F84
Drug use disorders	304	304, 305X	F11- F19, except F17
Bipolar disorder	296 except 2960, 2962	296 except 296B/X	F30-F31
Schizophrenia	295	295	F20

ADHD, attention deficit hyperactivity disorder; ICD, International Classification of Diseases

Table S2. Number of individuals included, number of cases, follow-up time, and incidence rates of any and major types of psychiatric disorders

	Females				Males			
	No. of individuals included	No. of incident cases during follow-up	Person-years during follow-up	IR	No. of individuals included	No. of incident cases during follow-up	Person-years during follow-up	IR
Any psychiatric disorder	4,607,668	604,271	59,293,572	10.2	4,628,707	549,851	60,127,336	9.1
Depressive disorders	4,738,208	282,342	63,578,832	4.4	4,791,399	186,986	65,022,892	2.9
Anxiety disorders	4,793,487	107,357	65,526,828	1.6	4,823,047	64,066	66,214,236	1.0
Stress-related disorders	4,785,065	154,502	65,084,456	2.4	4,816,128	85,875	65,979,664	1.3
Eating disorders	4,807,464	55,910	66,048,644	0.8	4,834,873	17,743	66,722,328	0.3
ADHD	4,816,817	86,189	66,152,396	1.3	4,832,976	128,956	66,029,672	2.0
Alcohol use disorders	4,784,149	79,821	65,579,708	1.2	4,755,569	131,215	64,926,248	2.0
Autism spectrum disorders	4,816,379	32,834	66,430,160	0.5	4,833,914	61,743	66,445,604	0.9
Drug use disorders	4,798,959	44,916	66,067,276	0.7	4,811,423	64,655	66,106,640	1.0
Bipolar disorder	4,805,141	42,446	66,168,040	0.6	4,829,221	25,577	66,595,524	0.4
Schizophrenia	4,806,811	5,771	66,456,096	0.1	4,823,802	7,769	66,643,836	0.1

ADHD, attention deficit hyperactivity disorder; IR, incidence rate per 1,000 person-years

Table S3. Standardized incidence rate of clinically diagnosed psychiatric disorders over the lifespan among females and males: a nationwide cohort study in Sweden, 2003-2019

Psychiatric disorders/age	Incidence rate per 1000 person-years	
	Females	Males
Any psychiatric disorder		
0	2.28 (2.19 to 2.38)	2.06 (1.97 to 2.15)
1	1.94 (1.85 to 2.03)	2.44 (2.34 to 2.54)
2	1.82 (1.73 to 1.91)	2.96 (2.85 to 3.07)
3	2.36 (2.25 to 2.46)	4.65 (4.51 to 4.79)
4	3.46 (3.34 to 3.59)	6.99 (6.81 to 7.16)
5	5.85 (5.68 to 6.01)	12.56 (12.32 to 12.79)
6	7.47 (7.28 to 7.66)	17.69 (17.41 to 17.97)
7	6.83 (6.65 to 7.01)	16.19 (15.91 to 16.46)
8	6.51 (6.33 to 6.69)	15.58 (15.31 to 15.85)
9	6.73 (6.55 to 6.91)	15.46 (15.19 to 15.74)
10	7.30 (7.11 to 7.49)	15.50 (15.22 to 15.78)
11	8.09 (7.89 to 8.29)	15.07 (14.79 to 15.34)
12	10.61 (10.37 to 10.84)	14.00 (13.73 to 14.26)
13	16.21 (15.92 to 16.50)	14.10 (13.83 to 14.37)
14	23.97 (23.62 to 24.33)	15.43 (15.15 to 15.71)
15	28.02 (27.63 to 28.41)	17.16 (16.86 to 17.46)
16	28.15 (27.75 to 28.54)	17.36 (17.06 to 17.66)
17	29.25 (28.85 to 29.66)	18.46 (18.15 to 18.77)
18	26.92 (26.53 to 27.31)	17.76 (17.46 to 18.07)
19	22.89 (22.54 to 23.25)	16.02 (15.73 to 16.31)
20	20.25 (19.91 to 20.58)	15.08 (14.80 to 15.36)
21	19.24 (18.91 to 19.57)	14.94 (14.66 to 15.22)
22	18.59 (18.26 to 18.91)	14.23 (13.96 to 14.50)
23	17.49 (17.17 to 17.80)	13.67 (13.40 to 13.94)
24	16.28 (15.97 to 16.59)	12.84 (12.58 to 13.10)
25	15.00 (14.71 to 15.30)	12.20 (11.95 to 12.45)
26	14.49 (14.19 to 14.78)	11.43 (11.18 to 11.68)
27	14.06 (13.76 to 14.35)	10.85 (10.61 to 11.09)
28	13.48 (13.19 to 13.77)	10.36 (10.12 to 10.60)
29	13.42 (13.13 to 13.71)	9.56 (9.33 to 9.79)
30	13.22 (12.93 to 13.51)	9.36 (9.13 to 9.59)
31	12.59 (12.31 to 12.87)	9.00 (8.78 to 9.23)
32	12.46 (12.18 to 12.75)	8.54 (8.31 to 8.76)
33	11.94 (11.67 to 12.22)	8.31 (8.09 to 8.53)
34	11.68 (11.41 to 11.95)	8.29 (8.08 to 8.51)
35	11.73 (11.46 to 12.01)	7.95 (7.74 to 8.16)
36	11.37 (11.11 to 11.64)	7.78 (7.57 to 7.98)
37	10.86 (10.60 to 11.11)	7.64 (7.44 to 7.84)
38	10.46 (10.21 to 10.71)	7.48 (7.28 to 7.68)
39	10.79 (10.54 to 11.04)	7.51 (7.31 to 7.71)
40	10.50 (10.25 to 10.74)	7.25 (7.06 to 7.45)
41	10.21 (9.97 to 10.45)	7.32 (7.12 to 7.51)
42	9.70 (9.47 to 9.93)	7.46 (7.26 to 7.65)

43	9.49 (9.26 to 9.71)	7.41 (7.22 to 7.61)
44	9.23 (9.00 to 9.45)	7.41 (7.22 to 7.61)
45	9.11 (8.88 to 9.33)	7.17 (6.98 to 7.36)
46	8.99 (8.77 to 9.21)	7.13 (6.94 to 7.33)
47	8.83 (8.61 to 9.05)	7.21 (7.02 to 7.40)
48	8.76 (8.54 to 8.98)	7.02 (6.83 to 7.21)
49	8.58 (8.36 to 8.80)	7.14 (6.95 to 7.33)
50	8.49 (8.27 to 8.70)	6.96 (6.77 to 7.15)
51	8.44 (8.22 to 8.65)	7.13 (6.94 to 7.32)
52	8.27 (8.06 to 8.48)	6.90 (6.71 to 7.09)
53	7.97 (7.76 to 8.18)	6.72 (6.53 to 6.91)
54	8.11 (7.90 to 8.32)	6.67 (6.49 to 6.86)
55	7.59 (7.39 to 7.79)	6.59 (6.41 to 6.78)
56	7.62 (7.41 to 7.82)	6.54 (6.35 to 6.72)
57	7.10 (6.90 to 7.29)	6.49 (6.31 to 6.68)
58	7.14 (6.94 to 7.34)	6.47 (6.28 to 6.65)
59	6.73 (6.54 to 6.92)	6.49 (6.31 to 6.67)
60	6.48 (6.29 to 6.66)	6.32 (6.14 to 6.50)
61	6.49 (6.30 to 6.67)	6.26 (6.08 to 6.44)
62	6.21 (6.02 to 6.39)	6.04 (5.86 to 6.22)
63	5.86 (5.68 to 6.04)	6.07 (5.89 to 6.26)
64	5.56 (5.39 to 5.74)	5.92 (5.74 to 6.10)
65	5.46 (5.29 to 5.64)	5.85 (5.67 to 6.03)
66	5.56 (5.38 to 5.73)	5.83 (5.65 to 6.01)
67	5.56 (5.38 to 5.74)	5.93 (5.74 to 6.12)
68	5.81 (5.62 to 6.00)	5.69 (5.51 to 5.88)
69	5.94 (5.74 to 6.13)	5.58 (5.39 to 5.77)
70	5.74 (5.55 to 5.94)	5.89 (5.70 to 6.09)
71	6.03 (5.83 to 6.23)	5.79 (5.59 to 5.99)
72	6.65 (6.43 to 6.86)	6.01 (5.80 to 6.22)
73	6.66 (6.44 to 6.88)	6.39 (6.17 to 6.61)
74	7.12 (6.89 to 7.35)	6.35 (6.12 to 6.58)
75	7.05 (6.82 to 7.28)	6.36 (6.13 to 6.60)
76	7.58 (7.34 to 7.83)	6.70 (6.46 to 6.95)
77	7.89 (7.63 to 8.15)	6.76 (6.51 to 7.02)
78	8.33 (8.06 to 8.59)	6.97 (6.70 to 7.24)
79	8.46 (8.18 to 8.73)	6.90 (6.62 to 7.17)
80	8.60 (8.32 to 8.88)	7.01 (6.72 to 7.29)
81	8.97 (8.68 to 9.26)	7.51 (7.21 to 7.82)
82	9.54 (9.23 to 9.84)	7.63 (7.32 to 7.95)
83	9.79 (9.48 to 10.11)	8.05 (7.71 to 8.39)
84	10.71 (10.37 to 11.05)	8.19 (7.83 to 8.55)
85	10.39 (10.04 to 10.73)	8.35 (7.97 to 8.73)
86	10.57 (10.21 to 10.94)	8.61 (8.20 to 9.03)
87	10.62 (10.23 to 11.00)	8.77 (8.32 to 9.22)
88	10.92 (10.51 to 11.33)	8.90 (8.41 to 9.39)
89	10.71 (10.28 to 11.14)	9.05 (8.51 to 9.59)
90	9.97 (9.52 to 10.42)	9.15 (8.55 to 9.76)

	91 10.02 (9.54 to 10.51)	8.71 (8.05 to 9.37)
	92 9.99 (9.45 to 10.52)	8.54 (7.80 to 9.28)
	93 9.91 (9.31 to 10.50)	8.49 (7.63 to 9.34)
	94 9.20 (8.55 to 9.84)	7.66 (6.70 to 8.61)
	95 8.55 (7.83 to 9.27)	8.34 (7.17 to 9.51)
	96 8.31 (7.49 to 9.13)	5.61 (4.47 to 6.75)
	97 7.09 (6.20 to 7.98)	5.03 (3.70 to 6.36)
	98 5.28 (4.37 to 6.19)	4.85 (3.30 to 6.40)
	99 5.14 (3.99 to 6.28)	2.59 (1.15 to 4.02)
	100 6.92 (3.13 to 10.71)	/
Depressive disorders		
	0 0.01 (0.00 to 0.01)	0.00 (0.00 to 0.01)
	1 0.00 (0.00 to 0.01)	0.01 (0.00 to 0.01)
	2 0.00 (0.00 to 0.01)	0.00 (0.00 to 0.00)
	3 0.00 (0.00 to 0.00)	0.00 (0.00 to 0.01)
	4 0.01 (0.00 to 0.01)	0.01 (0.00 to 0.01)
	5 0.01 (0.00 to 0.01)	0.01 (0.00 to 0.02)
	6 0.01 (0.01 to 0.02)	0.04 (0.02 to 0.05)
	7 0.03 (0.02 to 0.04)	0.10 (0.08 to 0.12)
	8 0.08 (0.06 to 0.10)	0.17 (0.14 to 0.19)
	9 0.15 (0.12 to 0.18)	0.36 (0.32 to 0.40)
	10 0.28 (0.24 to 0.31)	0.64 (0.59 to 0.70)
	11 0.76 (0.70 to 0.82)	1.04 (0.97 to 1.10)
	12 1.98 (1.89 to 2.08)	1.45 (1.37 to 1.53)
	13 4.63 (4.48 to 4.78)	1.99 (1.90 to 2.09)
	14 7.98 (7.78 to 8.18)	2.92 (2.81 to 3.04)
	15 10.08 (9.85 to 10.30)	3.74 (3.60 to 3.87)
	16 11.02 (10.79 to 11.26)	4.64 (4.49 to 4.79)
	17 12.45 (12.20 to 12.70)	5.54 (5.38 to 5.70)
	18 11.16 (10.92 to 11.39)	5.70 (5.53 to 5.86)
	19 9.51 (9.29 to 9.73)	5.34 (5.18 to 5.49)
	20 8.54 (8.33 to 8.74)	5.15 (5.00 to 5.31)
	21 8.50 (8.30 to 8.71)	5.36 (5.21 to 5.52)
	22 8.38 (8.17 to 8.59)	5.39 (5.23 to 5.54)
	23 8.32 (8.11 to 8.52)	5.39 (5.23 to 5.55)
	24 7.75 (7.55 to 7.95)	5.09 (4.94 to 5.25)
	25 7.40 (7.20 to 7.59)	5.00 (4.85 to 5.15)
	26 7.07 (6.88 to 7.27)	4.80 (4.65 to 4.95)
	27 7.28 (7.08 to 7.48)	4.78 (4.62 to 4.93)
	28 6.84 (6.64 to 7.03)	4.44 (4.29 to 4.58)
	29 6.94 (6.74 to 7.13)	4.25 (4.10 to 4.39)
	30 6.97 (6.77 to 7.16)	4.04 (3.89 to 4.18)
	31 6.61 (6.41 to 6.80)	3.98 (3.83 to 4.12)
	32 6.26 (6.07 to 6.45)	3.79 (3.65 to 3.94)
	33 6.19 (6.00 to 6.38)	3.67 (3.53 to 3.81)
	34 5.84 (5.66 to 6.02)	3.60 (3.46 to 3.74)
	35 5.86 (5.68 to 6.05)	3.42 (3.28 to 3.55)
	36 5.67 (5.49 to 5.85)	3.35 (3.22 to 3.48)

37	5.35 (5.18 to 5.52)	3.41 (3.28 to 3.54)
38	5.26 (5.09 to 5.43)	3.31 (3.18 to 3.44)
39	5.23 (5.06 to 5.39)	3.20 (3.07 to 3.32)
40	5.03 (4.87 to 5.20)	3.14 (3.02 to 3.27)
41	4.90 (4.75 to 5.06)	3.09 (2.97 to 3.22)
42	4.71 (4.55 to 4.86)	3.19 (3.06 to 3.31)
43	4.64 (4.49 to 4.79)	3.15 (3.03 to 3.27)
44	4.44 (4.30 to 4.59)	3.12 (3.00 to 3.24)
45	4.40 (4.25 to 4.54)	3.08 (2.96 to 3.20)
46	4.15 (4.00 to 4.29)	2.95 (2.83 to 3.06)
47	4.18 (4.04 to 4.33)	3.11 (2.99 to 3.23)
48	4.14 (3.99 to 4.28)	3.03 (2.91 to 3.15)
49	4.19 (4.05 to 4.34)	3.11 (2.99 to 3.24)
50	4.08 (3.94 to 4.23)	3.00 (2.88 to 3.12)
51	4.01 (3.87 to 4.15)	3.00 (2.88 to 3.12)
52	3.84 (3.70 to 3.98)	2.88 (2.76 to 3.00)
53	3.84 (3.70 to 3.98)	2.88 (2.76 to 3.00)
54	3.85 (3.71 to 3.99)	2.74 (2.62 to 2.85)
55	3.65 (3.52 to 3.79)	2.87 (2.75 to 2.99)
56	3.57 (3.44 to 3.71)	2.70 (2.59 to 2.82)
57	3.34 (3.21 to 3.47)	2.72 (2.61 to 2.84)
58	3.33 (3.20 to 3.46)	2.63 (2.51 to 2.74)
59	3.06 (2.94 to 3.18)	2.65 (2.54 to 2.76)
60	3.03 (2.90 to 3.15)	2.50 (2.39 to 2.61)
61	3.02 (2.90 to 3.14)	2.36 (2.26 to 2.47)
62	2.79 (2.67 to 2.91)	2.19 (2.08 to 2.29)
63	2.73 (2.61 to 2.85)	2.18 (2.08 to 2.29)
64	2.54 (2.43 to 2.66)	2.00 (1.90 to 2.10)
65	2.55 (2.43 to 2.66)	1.96 (1.86 to 2.06)
66	2.61 (2.49 to 2.73)	1.95 (1.84 to 2.05)
67	2.67 (2.55 to 2.79)	2.05 (1.95 to 2.16)
68	2.90 (2.77 to 3.03)	2.06 (1.95 to 2.17)
69	2.84 (2.71 to 2.97)	2.20 (2.08 to 2.31)
70	2.97 (2.84 to 3.11)	2.31 (2.19 to 2.43)
71	3.08 (2.94 to 3.22)	2.29 (2.16 to 2.41)
72	3.44 (3.29 to 3.59)	2.38 (2.25 to 2.51)
73	3.61 (3.45 to 3.77)	2.73 (2.58 to 2.87)
74	3.94 (3.77 to 4.11)	2.90 (2.75 to 3.05)
75	4.03 (3.85 to 4.20)	2.99 (2.84 to 3.15)
76	4.33 (4.15 to 4.51)	3.32 (3.15 to 3.49)
77	4.64 (4.45 to 4.83)	3.25 (3.08 to 3.42)
78	4.90 (4.70 to 5.10)	3.61 (3.43 to 3.80)
79	4.97 (4.76 to 5.17)	3.66 (3.47 to 3.86)
80	5.11 (4.90 to 5.32)	3.57 (3.37 to 3.77)
81	5.32 (5.10 to 5.53)	4.18 (3.96 to 4.40)
82	5.73 (5.50 to 5.96)	4.24 (4.01 to 4.47)
83	5.90 (5.66 to 6.14)	4.54 (4.29 to 4.79)
84	6.33 (6.08 to 6.59)	4.69 (4.42 to 4.95)

85	6.40 (6.13 to 6.66)	4.86 (4.58 to 5.15)
86	6.31 (6.03 to 6.58)	4.96 (4.65 to 5.27)
87	6.28 (5.99 to 6.57)	5.15 (4.81 to 5.48)
88	6.35 (6.04 to 6.65)	4.97 (4.61 to 5.33)
89	6.28 (5.96 to 6.60)	5.34 (4.94 to 5.75)
90	5.67 (5.34 to 6.00)	5.08 (4.64 to 5.53)
91	5.59 (5.23 to 5.94)	4.82 (4.34 to 5.31)
92	5.29 (4.90 to 5.67)	4.95 (4.39 to 5.50)
93	5.32 (4.89 to 5.75)	4.72 (4.09 to 5.35)
94	4.60 (4.15 to 5.05)	4.51 (3.79 to 5.22)
95	4.83 (4.30 to 5.36)	4.50 (3.65 to 5.35)
96	4.19 (3.61 to 4.76)	2.66 (1.88 to 3.45)
97	2.93 (2.37 to 3.50)	2.04 (1.20 to 2.89)
98	2.43 (1.81 to 3.04)	2.62 (1.51 to 3.74)
99	1.77 (1.10 to 2.45)	1.45 (0.35 to 2.54)
100	3.27 (0.64 to 5.90)	/
Anxiety disorders		
0	0.00 (0.00 to 0.00)	0.00 (0.00 to 0.00)
1	0.00 (0.00 to 0.00)	0.00 (0.00 to 0.01)
2	0.00 (0.00 to 0.00)	0.00 (0.00 to 0.01)
3	0.01 (0.00 to 0.01)	0.00 (0.00 to 0.01)
4	0.01 (0.00 to 0.02)	0.00 (0.00 to 0.01)
5	0.02 (0.01 to 0.04)	0.03 (0.02 to 0.04)
6	0.05 (0.03 to 0.06)	0.04 (0.03 to 0.06)
7	0.07 (0.06 to 0.09)	0.08 (0.06 to 0.10)
8	0.15 (0.13 to 0.18)	0.16 (0.13 to 0.18)
9	0.27 (0.24 to 0.31)	0.21 (0.18 to 0.25)
10	0.36 (0.32 to 0.40)	0.39 (0.35 to 0.43)
11	0.56 (0.51 to 0.61)	0.48 (0.44 to 0.53)
12	0.82 (0.75 to 0.88)	0.54 (0.49 to 0.59)
13	1.46 (1.37 to 1.54)	0.72 (0.66 to 0.77)
14	2.38 (2.27 to 2.49)	0.81 (0.74 to 0.87)
15	3.25 (3.12 to 3.37)	1.04 (0.97 to 1.11)
16	3.74 (3.60 to 3.88)	1.29 (1.21 to 1.37)
17	4.68 (4.53 to 4.83)	1.74 (1.65 to 1.83)
18	5.17 (5.01 to 5.33)	1.95 (1.86 to 2.05)
19	4.47 (4.32 to 4.62)	2.10 (2.00 to 2.19)
20	4.39 (4.24 to 4.53)	2.15 (2.05 to 2.25)
21	4.27 (4.12 to 4.41)	2.39 (2.29 to 2.49)
22	4.33 (4.18 to 4.47)	2.38 (2.28 to 2.48)
23	4.30 (4.16 to 4.45)	2.38 (2.28 to 2.48)
24	4.17 (4.03 to 4.31)	2.37 (2.27 to 2.48)
25	3.96 (3.82 to 4.10)	2.36 (2.26 to 2.46)
26	3.67 (3.54 to 3.81)	2.31 (2.21 to 2.42)
27	3.62 (3.48 to 3.75)	2.22 (2.12 to 2.32)
28	3.49 (3.36 to 3.63)	2.14 (2.03 to 2.24)
29	3.40 (3.27 to 3.54)	2.08 (1.98 to 2.18)
30	3.32 (3.19 to 3.46)	2.13 (2.02 to 2.23)

31	3.23 (3.10 to 3.36)	1.94 (1.84 to 2.04)
32	3.08 (2.95 to 3.21)	1.83 (1.74 to 1.93)
33	2.99 (2.86 to 3.12)	1.65 (1.56 to 1.74)
34	2.70 (2.58 to 2.82)	1.80 (1.70 to 1.89)
35	2.71 (2.59 to 2.84)	1.52 (1.43 to 1.61)
36	2.55 (2.43 to 2.67)	1.56 (1.47 to 1.64)
37	2.47 (2.36 to 2.59)	1.46 (1.37 to 1.54)
38	2.29 (2.18 to 2.40)	1.41 (1.32 to 1.49)
39	2.25 (2.14 to 2.36)	1.37 (1.29 to 1.45)
40	2.16 (2.06 to 2.27)	1.33 (1.25 to 1.41)
41	2.10 (2.00 to 2.20)	1.26 (1.19 to 1.34)
42	2.04 (1.94 to 2.14)	1.29 (1.21 to 1.36)
43	1.89 (1.79 to 1.99)	1.23 (1.15 to 1.30)
44	1.73 (1.64 to 1.82)	1.20 (1.13 to 1.28)
45	1.72 (1.63 to 1.81)	1.11 (1.04 to 1.18)
46	1.65 (1.56 to 1.74)	1.07 (1.00 to 1.14)
47	1.61 (1.52 to 1.70)	1.07 (1.00 to 1.14)
48	1.53 (1.45 to 1.62)	1.03 (0.96 to 1.10)
49	1.55 (1.46 to 1.63)	1.03 (0.96 to 1.10)
50	1.37 (1.29 to 1.45)	0.96 (0.89 to 1.02)
51	1.44 (1.36 to 1.52)	1.04 (0.97 to 1.11)
52	1.32 (1.24 to 1.40)	0.96 (0.90 to 1.03)
53	1.31 (1.23 to 1.39)	0.90 (0.83 to 0.96)
54	1.30 (1.22 to 1.38)	0.87 (0.80 to 0.93)
55	1.23 (1.15 to 1.30)	0.82 (0.76 to 0.88)
56	1.17 (1.09 to 1.24)	0.77 (0.71 to 0.83)
57	1.04 (0.96 to 1.11)	0.74 (0.68 to 0.80)
58	1.04 (0.97 to 1.12)	0.71 (0.66 to 0.77)
59	0.97 (0.90 to 1.04)	0.69 (0.63 to 0.74)
60	0.92 (0.85 to 0.98)	0.54 (0.49 to 0.59)
61	0.89 (0.83 to 0.96)	0.57 (0.52 to 0.62)
62	0.81 (0.75 to 0.87)	0.49 (0.44 to 0.54)
63	0.75 (0.69 to 0.81)	0.44 (0.39 to 0.48)
64	0.70 (0.64 to 0.76)	0.43 (0.39 to 0.48)
65	0.63 (0.57 to 0.68)	0.38 (0.34 to 0.43)
66	0.59 (0.53 to 0.64)	0.39 (0.34 to 0.43)
67	0.68 (0.62 to 0.74)	0.34 (0.30 to 0.39)
68	0.67 (0.61 to 0.73)	0.34 (0.30 to 0.39)
69	0.68 (0.62 to 0.74)	0.31 (0.27 to 0.35)
70	0.56 (0.50 to 0.62)	0.32 (0.28 to 0.36)
71	0.56 (0.50 to 0.62)	0.28 (0.23 to 0.32)
72	0.60 (0.54 to 0.67)	0.28 (0.24 to 0.33)
73	0.59 (0.52 to 0.65)	0.29 (0.24 to 0.33)
74	0.59 (0.53 to 0.66)	0.32 (0.27 to 0.37)
75	0.58 (0.51 to 0.64)	0.27 (0.23 to 0.32)
76	0.59 (0.53 to 0.66)	0.29 (0.24 to 0.34)
77	0.64 (0.57 to 0.70)	0.31 (0.26 to 0.36)
78	0.61 (0.55 to 0.68)	0.33 (0.28 to 0.39)

	79	0.64 (0.57 to 0.72)	0.27 (0.22 to 0.33)
	80	0.64 (0.57 to 0.72)	0.28 (0.22 to 0.33)
	81	0.57 (0.50 to 0.64)	0.30 (0.24 to 0.36)
	82	0.59 (0.52 to 0.67)	0.28 (0.22 to 0.34)
	83	0.53 (0.46 to 0.60)	0.31 (0.25 to 0.38)
	84	0.56 (0.48 to 0.63)	0.34 (0.27 to 0.41)
	85	0.50 (0.42 to 0.57)	0.34 (0.27 to 0.41)
	86	0.54 (0.46 to 0.61)	0.33 (0.26 to 0.41)
	87	0.55 (0.47 to 0.63)	0.28 (0.21 to 0.36)
	88	0.66 (0.57 to 0.76)	0.29 (0.20 to 0.38)
	89	0.52 (0.43 to 0.60)	0.37 (0.26 to 0.47)
	90	0.42 (0.33 to 0.51)	0.31 (0.20 to 0.41)
	91	0.43 (0.34 to 0.53)	0.22 (0.12 to 0.32)
	92	0.38 (0.28 to 0.48)	0.25 (0.13 to 0.36)
	93	0.36 (0.25 to 0.47)	0.14 (0.04 to 0.25)
	94	0.34 (0.22 to 0.46)	0.31 (0.13 to 0.50)
	95	0.27 (0.15 to 0.39)	0.34 (0.11 to 0.57)
	96	0.31 (0.16 to 0.46)	0.14 (0.00 to 0.31)
	97	0.29 (0.12 to 0.46)	0.44 (0.05 to 0.83)
	98	0.11 (0.00 to 0.24)	0.09 (0.00 to 0.27)
	99	0.30 (0.04 to 0.56)	/
	100	/	/
Stress-related disorders			
	0	0.01 (0.01 to 0.02)	0.02 (0.01 to 0.03)
	1	0.01 (0.00 to 0.01)	0.01 (0.00 to 0.01)
	2	0.00 (0.00 to 0.01)	0.01 (0.01 to 0.02)
	3	0.02 (0.01 to 0.03)	0.02 (0.01 to 0.03)
	4	0.03 (0.02 to 0.05)	0.04 (0.03 to 0.06)
	5	0.05 (0.04 to 0.07)	0.06 (0.05 to 0.08)
	6	0.07 (0.05 to 0.09)	0.13 (0.10 to 0.15)
	7	0.11 (0.09 to 0.13)	0.19 (0.16 to 0.22)
	8	0.12 (0.10 to 0.15)	0.25 (0.21 to 0.28)
	9	0.21 (0.18 to 0.25)	0.33 (0.29 to 0.37)
	10	0.33 (0.29 to 0.37)	0.44 (0.39 to 0.48)
	11	0.40 (0.35 to 0.44)	0.51 (0.47 to 0.56)
	12	0.73 (0.67 to 0.79)	0.55 (0.50 to 0.60)
	13	1.41 (1.33 to 1.50)	0.62 (0.57 to 0.67)
	14	2.29 (2.18 to 2.39)	0.85 (0.79 to 0.92)
	15	2.97 (2.85 to 3.09)	1.01 (0.94 to 1.08)
	16	3.40 (3.27 to 3.53)	1.17 (1.10 to 1.25)
	17	3.94 (3.80 to 4.08)	1.50 (1.42 to 1.58)
	18	4.64 (4.49 to 4.79)	1.94 (1.84 to 2.03)
	19	4.65 (4.50 to 4.80)	2.08 (1.98 to 2.17)
	20	4.39 (4.24 to 4.53)	2.10 (2.00 to 2.20)
	21	4.44 (4.29 to 4.59)	2.22 (2.12 to 2.32)
	22	4.40 (4.25 to 4.54)	2.29 (2.19 to 2.40)
	23	4.64 (4.49 to 4.79)	2.35 (2.25 to 2.45)
	24	4.46 (4.31 to 4.60)	2.30 (2.20 to 2.41)

25	4.22 (4.07 to 4.36)	2.27 (2.17 to 2.37)
26	4.26 (4.12 to 4.41)	2.41 (2.31 to 2.52)
27	4.28 (4.14 to 4.43)	2.26 (2.16 to 2.37)
28	4.42 (4.27 to 4.57)	2.34 (2.23 to 2.44)
29	4.51 (4.36 to 4.67)	2.32 (2.21 to 2.43)
30	4.61 (4.45 to 4.77)	2.24 (2.14 to 2.35)
31	4.66 (4.50 to 4.82)	2.22 (2.12 to 2.33)
32	4.84 (4.68 to 5.01)	2.25 (2.14 to 2.36)
33	4.49 (4.33 to 4.65)	2.35 (2.24 to 2.46)
34	4.73 (4.56 to 4.89)	2.28 (2.17 to 2.39)
35	4.72 (4.56 to 4.89)	2.35 (2.24 to 2.46)
36	4.77 (4.61 to 4.94)	2.33 (2.22 to 2.44)
37	4.63 (4.47 to 4.80)	2.24 (2.13 to 2.35)
38	4.41 (4.26 to 4.57)	2.22 (2.12 to 2.33)
39	4.49 (4.34 to 4.65)	2.22 (2.12 to 2.33)
40	4.69 (4.53 to 4.85)	2.22 (2.12 to 2.32)
41	4.52 (4.37 to 4.68)	2.18 (2.07 to 2.28)
42	4.34 (4.20 to 4.49)	2.20 (2.10 to 2.30)
43	4.12 (3.98 to 4.27)	2.14 (2.04 to 2.24)
44	4.15 (4.01 to 4.29)	2.11 (2.01 to 2.21)
45	4.14 (3.99 to 4.28)	2.01 (1.92 to 2.11)
46	3.77 (3.64 to 3.91)	2.01 (1.91 to 2.10)
47	3.93 (3.79 to 4.07)	2.02 (1.92 to 2.12)
48	3.63 (3.50 to 3.77)	1.95 (1.85 to 2.05)
49	3.58 (3.44 to 3.71)	1.96 (1.86 to 2.05)
50	3.38 (3.25 to 3.51)	1.94 (1.84 to 2.03)
51	3.36 (3.23 to 3.49)	1.83 (1.73 to 1.92)
52	3.14 (3.02 to 3.27)	1.76 (1.67 to 1.86)
53	3.02 (2.90 to 3.15)	1.72 (1.63 to 1.81)
54	3.08 (2.96 to 3.21)	1.55 (1.46 to 1.64)
55	2.72 (2.60 to 2.84)	1.59 (1.50 to 1.67)
56	2.64 (2.53 to 2.76)	1.54 (1.46 to 1.63)
57	2.36 (2.25 to 2.47)	1.42 (1.34 to 1.51)
58	2.32 (2.21 to 2.43)	1.43 (1.35 to 1.51)
59	2.26 (2.15 to 2.36)	1.35 (1.27 to 1.43)
60	2.02 (1.92 to 2.12)	1.18 (1.11 to 1.26)
61	1.80 (1.70 to 1.89)	1.13 (1.06 to 1.20)
62	1.60 (1.51 to 1.69)	0.91 (0.85 to 0.98)
63	1.31 (1.23 to 1.39)	0.86 (0.80 to 0.92)
64	1.03 (0.96 to 1.11)	0.63 (0.57 to 0.68)
65	0.84 (0.78 to 0.91)	0.54 (0.49 to 0.59)
66	0.84 (0.77 to 0.90)	0.51 (0.46 to 0.56)
67	0.76 (0.69 to 0.82)	0.53 (0.48 to 0.58)
68	0.74 (0.68 to 0.81)	0.50 (0.44 to 0.55)
69	0.70 (0.64 to 0.77)	0.42 (0.38 to 0.47)
70	0.63 (0.57 to 0.69)	0.42 (0.37 to 0.47)
71	0.69 (0.62 to 0.75)	0.44 (0.39 to 0.49)
72	0.62 (0.56 to 0.68)	0.43 (0.38 to 0.49)

73	0.65 (0.59 to 0.72)	0.48 (0.42 to 0.53)
74	0.65 (0.58 to 0.71)	0.40 (0.35 to 0.46)
75	0.66 (0.59 to 0.72)	0.43 (0.38 to 0.49)
76	0.64 (0.57 to 0.70)	0.42 (0.36 to 0.48)
77	0.60 (0.54 to 0.67)	0.49 (0.43 to 0.56)
78	0.59 (0.53 to 0.66)	0.43 (0.37 to 0.50)
79	0.56 (0.49 to 0.63)	0.47 (0.40 to 0.54)
80	0.56 (0.49 to 0.63)	0.40 (0.34 to 0.47)
81	0.56 (0.49 to 0.63)	0.45 (0.38 to 0.52)
82	0.58 (0.51 to 0.65)	0.48 (0.40 to 0.55)
83	0.53 (0.46 to 0.60)	0.47 (0.39 to 0.55)
84	0.55 (0.48 to 0.63)	0.47 (0.39 to 0.55)
85	0.50 (0.42 to 0.57)	0.41 (0.33 to 0.50)
86	0.48 (0.41 to 0.56)	0.41 (0.32 to 0.49)
87	0.43 (0.36 to 0.50)	0.37 (0.28 to 0.46)
88	0.38 (0.31 to 0.46)	0.52 (0.40 to 0.63)
89	0.37 (0.30 to 0.45)	0.35 (0.25 to 0.45)
90	0.30 (0.23 to 0.37)	0.43 (0.31 to 0.56)
91	0.27 (0.19 to 0.34)	0.36 (0.23 to 0.49)
92	0.25 (0.16 to 0.33)	0.44 (0.28 to 0.61)
93	0.25 (0.16 to 0.34)	0.49 (0.29 to 0.69)
94	0.22 (0.12 to 0.31)	0.28 (0.10 to 0.45)
95	0.17 (0.07 to 0.26)	0.41 (0.17 to 0.66)
96	0.13 (0.03 to 0.23)	0.44 (0.13 to 0.76)
97	0.05 (0.00 to 0.12)	0.29 (0.00 to 0.58)
98	0.04 (0.00 to 0.11)	0.12 (0.00 to 0.36)
99	0.16 (0.00 to 0.34)	/
100	/	/
Eating disorders		
0	0.05 (0.03 to 0.06)	0.07 (0.05 to 0.08)
1	0.08 (0.06 to 0.10)	0.09 (0.07 to 0.11)
2	0.05 (0.04 to 0.07)	0.08 (0.06 to 0.10)
3	0.04 (0.03 to 0.06)	0.07 (0.05 to 0.08)
4	0.04 (0.02 to 0.05)	0.06 (0.04 to 0.07)
5	0.04 (0.03 to 0.05)	0.05 (0.04 to 0.07)
6	0.05 (0.04 to 0.07)	0.06 (0.05 to 0.08)
7	0.08 (0.06 to 0.09)	0.05 (0.04 to 0.07)
8	0.08 (0.06 to 0.10)	0.06 (0.04 to 0.07)
9	0.14 (0.11 to 0.16)	0.11 (0.08 to 0.13)
10	0.31 (0.27 to 0.35)	0.12 (0.10 to 0.15)
11	0.66 (0.60 to 0.71)	0.22 (0.18 to 0.25)
12	1.50 (1.41 to 1.58)	0.25 (0.22 to 0.29)
13	2.59 (2.47 to 2.70)	0.31 (0.27 to 0.35)
14	3.99 (3.85 to 4.13)	0.37 (0.33 to 0.41)
15	4.58 (4.43 to 4.73)	0.31 (0.27 to 0.35)
16	4.57 (4.42 to 4.72)	0.32 (0.28 to 0.36)
17	4.48 (4.33 to 4.63)	0.39 (0.35 to 0.43)
18	3.69 (3.56 to 3.83)	0.31 (0.27 to 0.34)

19	3.12 (2.99 to 3.24)	0.25 (0.22 to 0.28)
20	2.78 (2.66 to 2.89)	0.28 (0.25 to 0.32)
21	2.58 (2.47 to 2.70)	0.34 (0.30 to 0.38)
22	2.73 (2.62 to 2.85)	0.35 (0.31 to 0.38)
23	2.40 (2.29 to 2.51)	0.39 (0.35 to 0.43)
24	2.41 (2.30 to 2.52)	0.38 (0.34 to 0.42)
25	2.05 (1.95 to 2.16)	0.37 (0.33 to 0.41)
26	1.88 (1.78 to 1.98)	0.32 (0.28 to 0.36)
27	1.62 (1.52 to 1.71)	0.35 (0.31 to 0.39)
28	1.53 (1.44 to 1.62)	0.41 (0.36 to 0.45)
29	1.45 (1.36 to 1.54)	0.34 (0.30 to 0.38)
30	1.36 (1.28 to 1.45)	0.31 (0.27 to 0.35)
31	1.23 (1.15 to 1.31)	0.36 (0.32 to 0.41)
32	1.12 (1.04 to 1.20)	0.34 (0.30 to 0.38)
33	1.04 (0.96 to 1.11)	0.34 (0.30 to 0.38)
34	0.97 (0.90 to 1.04)	0.35 (0.31 to 0.39)
35	0.90 (0.83 to 0.97)	0.35 (0.31 to 0.39)
36	0.82 (0.76 to 0.89)	0.30 (0.26 to 0.33)
37	0.75 (0.69 to 0.81)	0.34 (0.30 to 0.39)
38	0.70 (0.64 to 0.76)	0.34 (0.30 to 0.38)
39	0.62 (0.56 to 0.68)	0.28 (0.24 to 0.31)
40	0.61 (0.55 to 0.66)	0.31 (0.27 to 0.34)
41	0.51 (0.46 to 0.56)	0.30 (0.27 to 0.34)
42	0.50 (0.45 to 0.55)	0.33 (0.29 to 0.37)
43	0.43 (0.39 to 0.48)	0.32 (0.28 to 0.35)
44	0.40 (0.36 to 0.45)	0.30 (0.27 to 0.34)
45	0.40 (0.36 to 0.44)	0.30 (0.26 to 0.34)
46	0.43 (0.38 to 0.47)	0.32 (0.28 to 0.35)
47	0.38 (0.34 to 0.43)	0.30 (0.26 to 0.33)
48	0.40 (0.36 to 0.45)	0.33 (0.29 to 0.37)
49	0.38 (0.34 to 0.42)	0.32 (0.28 to 0.36)
50	0.37 (0.33 to 0.41)	0.31 (0.27 to 0.35)
51	0.42 (0.37 to 0.46)	0.32 (0.28 to 0.35)
52	0.41 (0.36 to 0.45)	0.32 (0.28 to 0.36)
53	0.41 (0.36 to 0.45)	0.32 (0.28 to 0.36)
54	0.37 (0.33 to 0.41)	0.34 (0.30 to 0.38)
55	0.38 (0.34 to 0.43)	0.31 (0.27 to 0.35)
56	0.38 (0.34 to 0.43)	0.29 (0.26 to 0.33)
57	0.32 (0.28 to 0.36)	0.30 (0.27 to 0.34)
58	0.33 (0.29 to 0.37)	0.32 (0.28 to 0.36)
59	0.25 (0.21 to 0.28)	0.28 (0.24 to 0.32)
60	0.23 (0.20 to 0.27)	0.33 (0.29 to 0.37)
61	0.20 (0.17 to 0.23)	0.33 (0.29 to 0.37)
62	0.18 (0.15 to 0.21)	0.33 (0.29 to 0.37)
63	0.19 (0.16 to 0.21)	0.37 (0.33 to 0.41)
64	0.16 (0.14 to 0.19)	0.31 (0.27 to 0.35)
65	0.14 (0.11 to 0.17)	0.31 (0.27 to 0.35)
66	0.13 (0.10 to 0.15)	0.31 (0.27 to 0.35)

67	0.16 (0.13 to 0.19)	0.33 (0.29 to 0.37)
68	0.11 (0.09 to 0.14)	0.28 (0.24 to 0.32)
69	0.09 (0.07 to 0.11)	0.27 (0.23 to 0.31)
70	0.11 (0.09 to 0.14)	0.29 (0.25 to 0.33)
71	0.08 (0.06 to 0.10)	0.27 (0.23 to 0.31)
72	0.09 (0.06 to 0.11)	0.29 (0.25 to 0.33)
73	0.06 (0.04 to 0.08)	0.23 (0.19 to 0.27)
74	0.09 (0.07 to 0.12)	0.22 (0.18 to 0.26)
75	0.06 (0.04 to 0.08)	0.19 (0.15 to 0.23)
76	0.05 (0.03 to 0.07)	0.22 (0.18 to 0.26)
77	0.06 (0.04 to 0.09)	0.16 (0.12 to 0.20)
78	0.06 (0.04 to 0.09)	0.15 (0.11 to 0.19)
79	0.07 (0.04 to 0.09)	0.15 (0.11 to 0.18)
80	0.05 (0.03 to 0.07)	0.11 (0.08 to 0.15)
81	0.07 (0.04 to 0.09)	0.08 (0.05 to 0.11)
82	0.05 (0.03 to 0.07)	0.12 (0.08 to 0.16)
83	0.08 (0.05 to 0.11)	0.08 (0.05 to 0.11)
84	0.07 (0.04 to 0.09)	0.06 (0.03 to 0.09)
85	0.06 (0.04 to 0.09)	0.07 (0.04 to 0.11)
86	0.03 (0.01 to 0.05)	0.07 (0.03 to 0.10)
87	0.10 (0.06 to 0.13)	0.06 (0.02 to 0.09)
88	0.08 (0.04 to 0.11)	0.06 (0.02 to 0.10)
89	0.05 (0.02 to 0.08)	0.14 (0.07 to 0.20)
90	0.06 (0.03 to 0.09)	0.07 (0.02 to 0.13)
91	0.06 (0.02 to 0.10)	0.04 (0.00 to 0.08)
92	0.08 (0.03 to 0.12)	0.05 (0.00 to 0.10)
93	0.04 (0.00 to 0.08)	0.08 (0.00 to 0.16)
94	0.02 (0.00 to 0.05)	/
95	0.04 (0.00 to 0.09)	/
96	/	/
97	0.03 (0.00 to 0.08)	/
98	/	/
99	/	/
100	/	/
ADHD		
0	0.00 (0.00 to 0.01)	0.00 (0.00 to 0.00)
1	0.01 (0.01 to 0.02)	0.04 (0.03 to 0.05)
2	0.05 (0.03 to 0.06)	0.16 (0.13 to 0.18)
3	0.15 (0.12 to 0.18)	0.46 (0.42 to 0.50)
4	0.36 (0.32 to 0.40)	1.21 (1.14 to 1.28)
5	0.89 (0.83 to 0.96)	2.85 (2.74 to 2.96)
6	1.50 (1.41 to 1.58)	4.91 (4.77 to 5.06)
7	2.05 (1.95 to 2.15)	6.61 (6.44 to 6.79)
8	2.67 (2.56 to 2.78)	8.53 (8.33 to 8.72)
9	3.22 (3.10 to 3.34)	9.38 (9.17 to 9.59)
10	3.43 (3.30 to 3.56)	9.67 (9.46 to 9.89)
11	3.34 (3.21 to 3.47)	9.27 (9.06 to 9.49)
12	3.45 (3.32 to 3.58)	8.52 (8.32 to 8.73)

13	4.10 (3.96 to 4.25)	7.82 (7.63 to 8.02)
14	5.44 (5.27 to 5.60)	7.83 (7.63 to 8.03)
15	6.46 (6.27 to 6.64)	7.23 (7.04 to 7.42)
16	6.29 (6.11 to 6.48)	5.88 (5.70 to 6.05)
17	6.52 (6.34 to 6.71)	5.57 (5.40 to 5.73)
18	5.28 (5.12 to 5.45)	4.69 (4.54 to 4.84)
19	3.38 (3.25 to 3.51)	2.95 (2.83 to 3.07)
20	2.78 (2.66 to 2.90)	2.55 (2.44 to 2.66)
21	2.54 (2.43 to 2.66)	2.39 (2.29 to 2.49)
22	2.52 (2.41 to 2.63)	2.32 (2.22 to 2.42)
23	2.43 (2.32 to 2.54)	2.31 (2.21 to 2.41)
24	2.37 (2.27 to 2.48)	2.48 (2.38 to 2.59)
25	2.28 (2.18 to 2.39)	2.27 (2.17 to 2.37)
26	2.19 (2.09 to 2.29)	2.48 (2.37 to 2.59)
27	2.08 (1.97 to 2.18)	2.21 (2.11 to 2.32)
28	2.01 (1.91 to 2.11)	2.17 (2.07 to 2.27)
29	1.90 (1.80 to 2.00)	2.21 (2.11 to 2.32)
30	1.95 (1.84 to 2.05)	2.06 (1.96 to 2.17)
31	1.82 (1.72 to 1.92)	1.92 (1.82 to 2.02)
32	1.75 (1.65 to 1.84)	1.85 (1.75 to 1.95)
33	1.71 (1.61 to 1.81)	1.81 (1.71 to 1.91)
34	1.66 (1.56 to 1.75)	1.69 (1.59 to 1.78)
35	1.61 (1.51 to 1.70)	1.60 (1.51 to 1.69)
36	1.59 (1.49 to 1.68)	1.50 (1.42 to 1.59)
37	1.43 (1.34 to 1.51)	1.58 (1.49 to 1.67)
38	1.37 (1.29 to 1.46)	1.35 (1.26 to 1.43)
39	1.29 (1.21 to 1.37)	1.33 (1.24 to 1.41)
40	1.30 (1.22 to 1.39)	1.26 (1.18 to 1.34)
41	1.24 (1.16 to 1.32)	1.14 (1.06 to 1.21)
42	1.10 (1.02 to 1.17)	1.13 (1.06 to 1.21)
43	1.05 (0.98 to 1.12)	1.07 (1.00 to 1.14)
44	0.94 (0.87 to 1.01)	1.04 (0.97 to 1.11)
45	0.90 (0.84 to 0.97)	0.91 (0.85 to 0.98)
46	0.86 (0.80 to 0.93)	0.87 (0.81 to 0.94)
47	0.82 (0.76 to 0.89)	0.89 (0.82 to 0.95)
48	0.75 (0.69 to 0.81)	0.77 (0.71 to 0.83)
49	0.64 (0.59 to 0.70)	0.76 (0.70 to 0.81)
50	0.62 (0.56 to 0.67)	0.65 (0.59 to 0.70)
51	0.54 (0.49 to 0.59)	0.64 (0.59 to 0.69)
52	0.49 (0.44 to 0.54)	0.54 (0.49 to 0.59)
53	0.45 (0.41 to 0.50)	0.54 (0.49 to 0.59)
54	0.40 (0.36 to 0.45)	0.42 (0.37 to 0.46)
55	0.37 (0.33 to 0.42)	0.46 (0.42 to 0.51)
56	0.28 (0.24 to 0.32)	0.37 (0.33 to 0.41)
57	0.26 (0.23 to 0.30)	0.30 (0.26 to 0.34)
58	0.23 (0.19 to 0.26)	0.25 (0.22 to 0.29)
59	0.16 (0.13 to 0.19)	0.23 (0.19 to 0.26)
60	0.12 (0.10 to 0.15)	0.16 (0.13 to 0.19)

61	0.12 (0.09 to 0.14)	0.12 (0.10 to 0.14)
62	0.08 (0.06 to 0.10)	0.12 (0.09 to 0.14)
63	0.09 (0.07 to 0.11)	0.12 (0.09 to 0.14)
64	0.06 (0.04 to 0.07)	0.08 (0.06 to 0.09)
65	0.06 (0.04 to 0.07)	0.04 (0.03 to 0.06)
66	0.05 (0.03 to 0.06)	0.07 (0.05 to 0.09)
67	0.02 (0.01 to 0.03)	0.04 (0.02 to 0.05)
68	0.03 (0.01 to 0.04)	0.04 (0.02 to 0.05)
69	0.03 (0.01 to 0.04)	0.03 (0.02 to 0.04)
70	0.01 (0.01 to 0.02)	0.02 (0.01 to 0.03)
71	0.02 (0.01 to 0.03)	0.02 (0.01 to 0.03)
72	0.01 (0.00 to 0.01)	0.01 (0.00 to 0.02)
73	0.01 (0.00 to 0.02)	0.02 (0.01 to 0.03)
74	0.01 (0.00 to 0.02)	0.01 (0.00 to 0.02)
75	0.01 (0.00 to 0.02)	0.01 (0.00 to 0.02)
76	0.00 (0.00 to 0.01)	0.02 (0.01 to 0.03)
77	0.01 (0.00 to 0.01)	0.01 (0.00 to 0.02)
78	0.01 (0.00 to 0.02)	0.02 (0.00 to 0.03)
79	0.00 (0.00 to 0.01)	0.00 (0.00 to 0.01)
80	0.01 (0.00 to 0.01)	0.01 (0.00 to 0.02)
81	0.00 (0.00 to 0.01)	0.01 (0.00 to 0.02)
82	0.01 (0.00 to 0.01)	0.01 (0.00 to 0.02)
83	0.02 (0.00 to 0.03)	/
84	0.01 (0.00 to 0.02)	0.00 (0.00 to 0.01)
85	0.01 (0.00 to 0.02)	0.01 (0.00 to 0.02)
86	0.01 (0.00 to 0.02)	0.01 (0.00 to 0.02)
87	0.00 (0.00 to 0.01)	/
88	0.01 (0.00 to 0.02)	0.01 (0.00 to 0.03)
89	/	/
90	/	/
91	/	/
92	0.01 (0.00 to 0.03)	0.02 (0.00 to 0.05)
93	/	/
94	0.01 (0.00 to 0.03)	/
95	/	/
96	/	/
97	/	/
98	/	/
99	/	/
100	/	/
Alcohol use disorders		
0	0.00 (0.00 to 0.01)	0.00 (0.00 to 0.00)
1	0.00 (0.00 to 0.01)	0.01 (0.01 to 0.02)
2	0.01 (0.00 to 0.01)	0.01 (0.00 to 0.01)
3	0.00 (0.00 to 0.01)	0.00 (0.00 to 0.00)
4	0.00 (0.00 to 0.01)	0.00 (0.00 to 0.00)
5	0.01 (0.00 to 0.01)	0.00 (0.00 to 0.01)
6	/	0.00 (0.00 to 0.01)

7	0.00 (0.00 to 0.01)	0.00 (0.00 to 0.01)
8	0.00 (0.00 to 0.01)	0.01 (0.00 to 0.01)
9	0.00 (0.00 to 0.01)	0.01 (0.00 to 0.01)
10	0.00 (0.00 to 0.00)	0.02 (0.01 to 0.03)
11	0.02 (0.01 to 0.03)	0.03 (0.02 to 0.04)
12	0.25 (0.21 to 0.28)	0.17 (0.14 to 0.19)
13	1.18 (1.10 to 1.25)	0.74 (0.68 to 0.80)
14	2.77 (2.66 to 2.89)	1.69 (1.60 to 1.77)
15	3.61 (3.48 to 3.74)	2.93 (2.81 to 3.04)
16	3.67 (3.54 to 3.80)	3.53 (3.40 to 3.65)
17	3.95 (3.82 to 4.09)	3.83 (3.70 to 3.96)
18	5.20 (5.04 to 5.35)	4.43 (4.29 to 4.57)
19	4.78 (4.63 to 4.93)	4.47 (4.33 to 4.61)
20	4.01 (3.87 to 4.14)	3.94 (3.81 to 4.08)
21	3.30 (3.17 to 3.43)	3.59 (3.47 to 3.72)
22	2.88 (2.76 to 3.00)	3.45 (3.32 to 3.58)
23	2.45 (2.34 to 2.55)	3.24 (3.12 to 3.36)
24	2.05 (1.95 to 2.15)	2.95 (2.83 to 3.06)
25	1.84 (1.74 to 1.94)	2.62 (2.51 to 2.73)
26	1.58 (1.49 to 1.67)	2.51 (2.40 to 2.62)
27	1.36 (1.28 to 1.45)	2.33 (2.23 to 2.44)
28	1.34 (1.26 to 1.42)	2.22 (2.12 to 2.33)
29	1.21 (1.13 to 1.29)	2.16 (2.06 to 2.27)
30	1.08 (1.00 to 1.15)	2.10 (2.00 to 2.20)
31	1.13 (1.05 to 1.21)	2.05 (1.95 to 2.16)
32	1.03 (0.95 to 1.11)	1.96 (1.86 to 2.06)
33	0.98 (0.90 to 1.05)	1.92 (1.82 to 2.02)
34	1.01 (0.94 to 1.08)	1.93 (1.83 to 2.03)
35	0.99 (0.92 to 1.07)	1.89 (1.79 to 1.99)
36	0.96 (0.89 to 1.04)	1.96 (1.86 to 2.06)
37	1.06 (0.98 to 1.13)	1.96 (1.86 to 2.06)
38	1.05 (0.98 to 1.12)	1.94 (1.85 to 2.04)
39	1.13 (1.05 to 1.20)	2.04 (1.94 to 2.14)
40	1.26 (1.18 to 1.34)	2.04 (1.95 to 2.14)
41	1.29 (1.21 to 1.37)	2.08 (1.98 to 2.18)
42	1.24 (1.16 to 1.32)	2.19 (2.09 to 2.29)
43	1.32 (1.24 to 1.40)	2.34 (2.23 to 2.44)
44	1.33 (1.25 to 1.41)	2.39 (2.28 to 2.49)
45	1.39 (1.31 to 1.47)	2.42 (2.32 to 2.53)
46	1.38 (1.29 to 1.46)	2.49 (2.39 to 2.60)
47	1.45 (1.37 to 1.54)	2.56 (2.45 to 2.67)
48	1.46 (1.38 to 1.55)	2.51 (2.40 to 2.62)
49	1.37 (1.29 to 1.45)	2.72 (2.60 to 2.83)
50	1.40 (1.32 to 1.48)	2.59 (2.48 to 2.70)
51	1.38 (1.29 to 1.46)	2.72 (2.61 to 2.84)
52	1.41 (1.32 to 1.49)	2.74 (2.62 to 2.85)
53	1.33 (1.25 to 1.41)	2.61 (2.50 to 2.73)
54	1.35 (1.26 to 1.43)	2.72 (2.61 to 2.84)

55	1.25 (1.18 to 1.33)	2.61 (2.50 to 2.72)
56	1.26 (1.18 to 1.34)	2.65 (2.54 to 2.77)
57	1.19 (1.12 to 1.27)	2.62 (2.51 to 2.74)
58	1.16 (1.08 to 1.23)	2.66 (2.54 to 2.77)
59	1.09 (1.02 to 1.17)	2.75 (2.64 to 2.87)
60	1.05 (0.98 to 1.12)	2.73 (2.61 to 2.84)
61	1.05 (0.98 to 1.12)	2.80 (2.68 to 2.92)
62	1.10 (1.03 to 1.18)	2.60 (2.48 to 2.71)
63	1.07 (0.99 to 1.14)	2.65 (2.53 to 2.76)
64	1.06 (0.99 to 1.13)	2.77 (2.65 to 2.89)
65	1.05 (0.97 to 1.12)	2.75 (2.63 to 2.87)
66	0.99 (0.92 to 1.06)	2.69 (2.57 to 2.81)
67	0.98 (0.91 to 1.05)	2.65 (2.52 to 2.77)
68	0.97 (0.90 to 1.04)	2.51 (2.39 to 2.63)
69	0.95 (0.87 to 1.02)	2.32 (2.20 to 2.43)
70	0.77 (0.71 to 0.84)	2.24 (2.13 to 2.36)
71	0.82 (0.75 to 0.89)	2.15 (2.03 to 2.27)
72	0.84 (0.77 to 0.92)	2.05 (1.93 to 2.17)
73	0.68 (0.61 to 0.74)	2.11 (1.99 to 2.23)
74	0.68 (0.62 to 0.75)	1.91 (1.79 to 2.03)
75	0.60 (0.54 to 0.67)	1.80 (1.68 to 1.91)
76	0.57 (0.51 to 0.63)	1.71 (1.59 to 1.83)
77	0.53 (0.47 to 0.59)	1.68 (1.55 to 1.80)
78	0.53 (0.47 to 0.60)	1.60 (1.48 to 1.73)
79	0.45 (0.39 to 0.51)	1.40 (1.28 to 1.52)
80	0.47 (0.40 to 0.53)	1.41 (1.29 to 1.53)
81	0.39 (0.33 to 0.45)	1.32 (1.20 to 1.45)
82	0.39 (0.33 to 0.44)	1.10 (0.98 to 1.22)
83	0.37 (0.31 to 0.43)	0.99 (0.88 to 1.11)
84	0.36 (0.30 to 0.42)	0.99 (0.87 to 1.11)
85	0.23 (0.18 to 0.27)	0.95 (0.83 to 1.08)
86	0.27 (0.22 to 0.33)	0.79 (0.67 to 0.91)
87	0.23 (0.18 to 0.28)	0.63 (0.51 to 0.74)
88	0.26 (0.20 to 0.32)	0.62 (0.49 to 0.74)
89	0.17 (0.12 to 0.22)	0.48 (0.36 to 0.60)
90	0.15 (0.10 to 0.21)	0.55 (0.41 to 0.70)
91	0.12 (0.07 to 0.17)	0.47 (0.33 to 0.62)
92	0.16 (0.10 to 0.23)	0.40 (0.25 to 0.56)
93	0.13 (0.06 to 0.19)	0.28 (0.13 to 0.43)
94	0.14 (0.06 to 0.21)	0.31 (0.11 to 0.50)
95	0.12 (0.04 to 0.20)	0.24 (0.05 to 0.43)
96	0.07 (0.00 to 0.14)	0.28 (0.03 to 0.54)
97	0.05 (0.00 to 0.12)	0.15 (0.00 to 0.36)
98	/	0.12 (0.00 to 0.34)
99	0.06 (0.00 to 0.18)	/
100	/	/
Autism spectrum disorders		
0	0.03 (0.02 to 0.04)	0.04 (0.02 to 0.05)

1	0.11 (0.09 to 0.13)	0.20 (0.17 to 0.22)
2	0.41 (0.37 to 0.46)	1.20 (1.13 to 1.27)
3	0.81 (0.75 to 0.87)	2.58 (2.48 to 2.68)
4	0.77 (0.72 to 0.83)	2.73 (2.63 to 2.84)
5	0.77 (0.71 to 0.83)	2.61 (2.50 to 2.71)
6	0.77 (0.71 to 0.83)	2.61 (2.51 to 2.72)
7	0.78 (0.72 to 0.84)	2.44 (2.34 to 2.54)
8	0.81 (0.75 to 0.87)	2.73 (2.62 to 2.84)
9	0.94 (0.87 to 1.00)	3.06 (2.94 to 3.18)
10	1.19 (1.11 to 1.26)	3.26 (3.14 to 3.39)
11	1.38 (1.30 to 1.47)	3.44 (3.31 to 3.56)
12	1.56 (1.47 to 1.65)	3.54 (3.41 to 3.67)
13	1.84 (1.74 to 1.93)	3.38 (3.25 to 3.51)
14	2.20 (2.09 to 2.31)	3.22 (3.09 to 3.34)
15	2.47 (2.36 to 2.58)	3.30 (3.17 to 3.43)
16	2.31 (2.20 to 2.42)	2.86 (2.74 to 2.98)
17	2.34 (2.23 to 2.45)	2.74 (2.63 to 2.86)
18	1.77 (1.68 to 1.86)	2.33 (2.22 to 2.43)
19	1.26 (1.18 to 1.34)	1.62 (1.54 to 1.71)
20	1.06 (0.99 to 1.13)	1.52 (1.44 to 1.61)
21	1.03 (0.96 to 1.10)	1.40 (1.32 to 1.48)
22	0.89 (0.83 to 0.95)	1.30 (1.22 to 1.37)
23	0.81 (0.75 to 0.87)	1.19 (1.12 to 1.27)
24	0.80 (0.74 to 0.87)	1.18 (1.11 to 1.26)
25	0.76 (0.70 to 0.82)	1.04 (0.97 to 1.11)
26	0.72 (0.67 to 0.78)	1.08 (1.01 to 1.15)
27	0.72 (0.66 to 0.78)	1.00 (0.93 to 1.07)
28	0.66 (0.60 to 0.71)	0.93 (0.86 to 0.99)
29	0.66 (0.61 to 0.72)	0.93 (0.86 to 1.00)
30	0.59 (0.54 to 0.65)	0.81 (0.74 to 0.87)
31	0.58 (0.52 to 0.63)	0.82 (0.75 to 0.88)
32	0.48 (0.43 to 0.54)	0.69 (0.63 to 0.75)
33	0.46 (0.41 to 0.51)	0.68 (0.62 to 0.74)
34	0.47 (0.41 to 0.52)	0.55 (0.49 to 0.60)
35	0.45 (0.40 to 0.50)	0.58 (0.53 to 0.64)
36	0.40 (0.36 to 0.45)	0.50 (0.45 to 0.55)
37	0.41 (0.36 to 0.45)	0.52 (0.47 to 0.57)
38	0.37 (0.33 to 0.42)	0.45 (0.40 to 0.49)
39	0.35 (0.31 to 0.40)	0.43 (0.39 to 0.48)
40	0.34 (0.30 to 0.38)	0.40 (0.36 to 0.45)
41	0.35 (0.31 to 0.39)	0.41 (0.37 to 0.46)
42	0.31 (0.28 to 0.35)	0.39 (0.35 to 0.43)
43	0.30 (0.27 to 0.34)	0.39 (0.34 to 0.43)
44	0.25 (0.22 to 0.29)	0.31 (0.28 to 0.35)
45	0.26 (0.23 to 0.30)	0.33 (0.29 to 0.37)
46	0.24 (0.20 to 0.27)	0.33 (0.29 to 0.37)
47	0.20 (0.17 to 0.23)	0.29 (0.25 to 0.32)
48	0.21 (0.18 to 0.24)	0.28 (0.24 to 0.31)

49	0.18 (0.15 to 0.21)	0.26 (0.23 to 0.29)
50	0.16 (0.13 to 0.18)	0.28 (0.24 to 0.31)
51	0.16 (0.13 to 0.19)	0.23 (0.20 to 0.26)
52	0.18 (0.15 to 0.21)	0.22 (0.18 to 0.25)
53	0.14 (0.11 to 0.16)	0.22 (0.19 to 0.25)
54	0.12 (0.09 to 0.14)	0.18 (0.15 to 0.21)
55	0.13 (0.10 to 0.15)	0.19 (0.16 to 0.22)
56	0.12 (0.09 to 0.14)	0.16 (0.14 to 0.19)
57	0.10 (0.08 to 0.12)	0.14 (0.12 to 0.17)
58	0.09 (0.07 to 0.11)	0.15 (0.12 to 0.17)
59	0.06 (0.05 to 0.08)	0.14 (0.11 to 0.16)
60	0.07 (0.05 to 0.09)	0.10 (0.08 to 0.13)
61	0.05 (0.04 to 0.07)	0.10 (0.08 to 0.12)
62	0.05 (0.03 to 0.06)	0.09 (0.07 to 0.11)
63	0.04 (0.03 to 0.06)	0.08 (0.06 to 0.10)
64	0.06 (0.04 to 0.08)	0.07 (0.05 to 0.09)
65	0.03 (0.02 to 0.04)	0.06 (0.05 to 0.08)
66	0.02 (0.01 to 0.03)	0.05 (0.04 to 0.07)
67	0.03 (0.02 to 0.04)	0.05 (0.04 to 0.07)
68	0.03 (0.02 to 0.04)	0.05 (0.03 to 0.07)
69	0.02 (0.01 to 0.03)	0.03 (0.01 to 0.04)
70	0.01 (0.00 to 0.02)	0.04 (0.03 to 0.06)
71	0.02 (0.01 to 0.03)	0.04 (0.03 to 0.06)
72	0.02 (0.01 to 0.02)	0.03 (0.01 to 0.04)
73	0.02 (0.01 to 0.03)	0.02 (0.01 to 0.03)
74	0.01 (0.00 to 0.02)	0.01 (0.00 to 0.01)
75	0.01 (0.00 to 0.02)	0.02 (0.01 to 0.03)
76	0.02 (0.01 to 0.03)	0.01 (0.00 to 0.03)
77	0.00 (0.00 to 0.01)	0.03 (0.02 to 0.05)
78	0.01 (0.00 to 0.01)	0.04 (0.02 to 0.05)
79	0.02 (0.01 to 0.03)	0.02 (0.01 to 0.04)
80	0.00 (0.00 to 0.01)	0.02 (0.01 to 0.04)
81	0.01 (0.00 to 0.02)	0.01 (0.00 to 0.03)
82	0.02 (0.01 to 0.03)	0.00 (0.00 to 0.01)
83	0.01 (0.00 to 0.03)	0.01 (0.00 to 0.02)
84	0.01 (0.00 to 0.01)	0.02 (0.00 to 0.03)
85	/	0.00 (0.00 to 0.01)
86	0.00 (0.00 to 0.01)	0.02 (0.00 to 0.04)
87	0.02 (0.00 to 0.03)	0.02 (0.00 to 0.05)
88	0.00 (0.00 to 0.01)	0.01 (0.00 to 0.03)
89	0.00 (0.00 to 0.01)	0.01 (0.00 to 0.02)
90	/	0.01 (0.00 to 0.03)
91	0.01 (0.00 to 0.02)	/
92	/	/
93	/	/
94	/	/
95	/	/
96	/	/

97	/	/
98	/	/
99	/	/
100	/	/
Drug use disorders		
0	0.03 (0.02 to 0.04)	0.03 (0.02 to 0.04)
1	0.06 (0.05 to 0.08)	0.09 (0.07 to 0.11)
2	0.06 (0.05 to 0.08)	0.07 (0.06 to 0.09)
3	0.02 (0.01 to 0.03)	0.04 (0.02 to 0.05)
4	0.00 (0.00 to 0.01)	0.02 (0.01 to 0.03)
5	0.02 (0.01 to 0.03)	0.01 (0.00 to 0.02)
6	0.01 (0.00 to 0.01)	0.01 (0.00 to 0.02)
7	0.01 (0.00 to 0.01)	0.01 (0.00 to 0.01)
8	0.01 (0.00 to 0.02)	0.01 (0.00 to 0.02)
9	0.01 (0.00 to 0.01)	0.01 (0.00 to 0.01)
10	0.00 (0.00 to 0.01)	0.02 (0.01 to 0.03)
11	0.02 (0.01 to 0.03)	0.03 (0.02 to 0.04)
12	0.09 (0.07 to 0.11)	0.06 (0.04 to 0.07)
13	0.35 (0.31 to 0.40)	0.25 (0.21 to 0.28)
14	0.91 (0.85 to 0.98)	0.84 (0.77 to 0.90)
15	1.36 (1.28 to 1.44)	1.54 (1.46 to 1.63)
16	1.74 (1.65 to 1.83)	2.29 (2.18 to 2.39)
17	2.03 (1.93 to 2.13)	3.10 (2.98 to 3.22)
18	2.53 (2.42 to 2.64)	3.36 (3.24 to 3.49)
19	2.59 (2.48 to 2.71)	3.69 (3.56 to 3.82)
20	2.46 (2.36 to 2.57)	3.64 (3.51 to 3.76)
21	2.26 (2.15 to 2.36)	3.91 (3.77 to 4.04)
22	2.05 (1.95 to 2.15)	3.54 (3.42 to 3.67)
23	1.93 (1.83 to 2.02)	3.45 (3.33 to 3.58)
24	1.61 (1.52 to 1.70)	3.11 (3.00 to 3.23)
25	1.50 (1.42 to 1.59)	2.89 (2.78 to 3.01)
26	1.28 (1.20 to 1.36)	2.67 (2.56 to 2.78)
27	1.17 (1.09 to 1.24)	2.42 (2.31 to 2.52)
28	1.10 (1.03 to 1.18)	2.11 (2.01 to 2.21)
29	0.97 (0.90 to 1.05)	1.98 (1.88 to 2.08)
30	1.02 (0.95 to 1.09)	1.71 (1.62 to 1.81)
31	0.85 (0.78 to 0.92)	1.72 (1.62 to 1.81)
32	0.92 (0.85 to 0.99)	1.40 (1.32 to 1.49)
33	0.79 (0.72 to 0.85)	1.36 (1.27 to 1.44)
34	0.74 (0.67 to 0.80)	1.22 (1.14 to 1.30)
35	0.74 (0.68 to 0.80)	1.13 (1.06 to 1.21)
36	0.72 (0.65 to 0.78)	1.11 (1.04 to 1.19)
37	0.79 (0.73 to 0.86)	1.05 (0.98 to 1.12)
38	0.72 (0.66 to 0.78)	0.96 (0.89 to 1.03)
39	0.74 (0.68 to 0.80)	0.93 (0.86 to 1.00)
40	0.73 (0.67 to 0.79)	0.84 (0.78 to 0.90)
41	0.79 (0.72 to 0.85)	0.87 (0.81 to 0.93)
42	0.72 (0.66 to 0.78)	0.80 (0.74 to 0.86)

43	0.72 (0.66 to 0.78)	0.89 (0.82 to 0.95)
44	0.73 (0.67 to 0.79)	0.83 (0.77 to 0.89)
45	0.72 (0.66 to 0.78)	0.80 (0.74 to 0.86)
46	0.69 (0.63 to 0.74)	0.78 (0.72 to 0.84)
47	0.67 (0.61 to 0.73)	0.84 (0.77 to 0.90)
48	0.70 (0.64 to 0.75)	0.74 (0.68 to 0.79)
49	0.64 (0.59 to 0.70)	0.79 (0.73 to 0.85)
50	0.68 (0.62 to 0.74)	0.76 (0.70 to 0.82)
51	0.60 (0.55 to 0.65)	0.72 (0.66 to 0.77)
52	0.59 (0.53 to 0.64)	0.67 (0.62 to 0.73)
53	0.56 (0.50 to 0.61)	0.70 (0.64 to 0.76)
54	0.55 (0.50 to 0.60)	0.61 (0.55 to 0.66)
55	0.57 (0.52 to 0.62)	0.61 (0.56 to 0.67)
56	0.55 (0.50 to 0.60)	0.58 (0.53 to 0.63)
57	0.48 (0.43 to 0.53)	0.53 (0.48 to 0.58)
58	0.51 (0.46 to 0.56)	0.52 (0.47 to 0.57)
59	0.45 (0.40 to 0.49)	0.50 (0.45 to 0.55)
60	0.40 (0.36 to 0.45)	0.51 (0.46 to 0.56)
61	0.41 (0.37 to 0.46)	0.47 (0.42 to 0.52)
62	0.41 (0.37 to 0.46)	0.41 (0.37 to 0.46)
63	0.39 (0.35 to 0.44)	0.37 (0.33 to 0.41)
64	0.36 (0.31 to 0.40)	0.38 (0.33 to 0.42)
65	0.36 (0.32 to 0.40)	0.37 (0.33 to 0.42)
66	0.35 (0.31 to 0.40)	0.37 (0.32 to 0.41)
67	0.37 (0.32 to 0.41)	0.32 (0.28 to 0.36)
68	0.34 (0.30 to 0.38)	0.33 (0.28 to 0.37)
69	0.35 (0.31 to 0.40)	0.29 (0.25 to 0.33)
70	0.31 (0.27 to 0.35)	0.31 (0.27 to 0.35)
71	0.36 (0.32 to 0.41)	0.26 (0.22 to 0.30)
72	0.30 (0.26 to 0.34)	0.29 (0.25 to 0.33)
73	0.31 (0.26 to 0.35)	0.25 (0.21 to 0.30)
74	0.38 (0.33 to 0.43)	0.29 (0.25 to 0.34)
75	0.32 (0.27 to 0.36)	0.31 (0.26 to 0.36)
76	0.39 (0.34 to 0.44)	0.30 (0.25 to 0.35)
77	0.36 (0.30 to 0.41)	0.22 (0.17 to 0.26)
78	0.33 (0.28 to 0.38)	0.26 (0.21 to 0.31)
79	0.32 (0.27 to 0.37)	0.22 (0.17 to 0.26)
80	0.36 (0.30 to 0.41)	0.25 (0.20 to 0.30)
81	0.34 (0.29 to 0.39)	0.29 (0.23 to 0.35)
82	0.35 (0.29 to 0.41)	0.21 (0.16 to 0.26)
83	0.35 (0.29 to 0.40)	0.30 (0.24 to 0.36)
84	0.39 (0.33 to 0.45)	0.23 (0.17 to 0.28)
85	0.33 (0.27 to 0.39)	0.28 (0.21 to 0.35)
86	0.37 (0.30 to 0.43)	0.36 (0.28 to 0.45)
87	0.32 (0.25 to 0.38)	0.31 (0.23 to 0.39)
88	0.39 (0.31 to 0.46)	0.42 (0.31 to 0.52)
89	0.46 (0.38 to 0.55)	0.30 (0.21 to 0.40)
90	0.36 (0.28 to 0.44)	0.32 (0.21 to 0.43)

91	0.29 (0.22 to 0.37)	0.54 (0.38 to 0.70)
92	0.30 (0.21 to 0.39)	0.27 (0.15 to 0.39)
93	0.32 (0.22 to 0.42)	0.30 (0.14 to 0.45)
94	0.38 (0.25 to 0.50)	0.22 (0.07 to 0.38)
95	0.26 (0.14 to 0.38)	0.34 (0.10 to 0.57)
96	0.31 (0.16 to 0.46)	0.21 (0.00 to 0.42)
97	0.25 (0.08 to 0.41)	0.29 (0.00 to 0.63)
98	0.22 (0.04 to 0.40)	0.12 (0.00 to 0.36)
99	0.17 (0.00 to 0.37)	0.33 (0.00 to 0.80)
100	/	/
Bipolar disorder		
0	0.00 (0.00 to 0.00)	0.00 (0.00 to 0.00)
1	/	/
2	/	0.00 (0.00 to 0.00)
3	0.00 (0.00 to 0.00)	/
4	/	0.00 (0.00 to 0.01)
5	0.00 (0.00 to 0.01)	0.00 (0.00 to 0.01)
6	0.01 (0.00 to 0.01)	0.01 (0.00 to 0.02)
7	0.01 (0.00 to 0.02)	0.01 (0.01 to 0.02)
8	0.02 (0.01 to 0.03)	0.03 (0.02 to 0.04)
9	0.01 (0.00 to 0.02)	0.04 (0.02 to 0.05)
10	0.02 (0.01 to 0.03)	0.05 (0.04 to 0.07)
11	0.03 (0.02 to 0.04)	0.07 (0.05 to 0.08)
12	0.08 (0.06 to 0.10)	0.07 (0.05 to 0.09)
13	0.13 (0.10 to 0.15)	0.08 (0.06 to 0.10)
14	0.28 (0.24 to 0.32)	0.13 (0.11 to 0.16)
15	0.42 (0.38 to 0.47)	0.15 (0.12 to 0.17)
16	0.61 (0.56 to 0.66)	0.21 (0.18 to 0.25)
17	0.97 (0.90 to 1.04)	0.34 (0.30 to 0.38)
18	1.36 (1.28 to 1.44)	0.45 (0.41 to 0.50)
19	1.44 (1.35 to 1.52)	0.50 (0.46 to 0.55)
20	1.43 (1.35 to 1.51)	0.51 (0.46 to 0.56)
21	1.60 (1.51 to 1.69)	0.64 (0.58 to 0.69)
22	1.63 (1.55 to 1.72)	0.71 (0.65 to 0.76)
23	1.62 (1.53 to 1.71)	0.68 (0.63 to 0.74)
24	1.63 (1.54 to 1.71)	0.77 (0.71 to 0.83)
25	1.55 (1.46 to 1.63)	0.75 (0.70 to 0.81)
26	1.48 (1.40 to 1.57)	0.77 (0.71 to 0.83)
27	1.44 (1.35 to 1.53)	0.74 (0.68 to 0.80)
28	1.47 (1.38 to 1.56)	0.74 (0.68 to 0.80)
29	1.38 (1.30 to 1.47)	0.76 (0.70 to 0.82)
30	1.34 (1.25 to 1.42)	0.73 (0.67 to 0.79)
31	1.31 (1.22 to 1.39)	0.75 (0.69 to 0.81)
32	1.33 (1.24 to 1.41)	0.67 (0.61 to 0.73)
33	1.25 (1.17 to 1.34)	0.63 (0.57 to 0.68)
34	1.23 (1.15 to 1.32)	0.69 (0.63 to 0.75)
35	1.18 (1.10 to 1.26)	0.67 (0.61 to 0.73)
36	1.20 (1.12 to 1.28)	0.64 (0.59 to 0.70)

37	1.09 (1.02 to 1.17)	0.58 (0.53 to 0.64)
38	1.08 (1.00 to 1.15)	0.62 (0.57 to 0.68)
39	1.04 (0.97 to 1.11)	0.59 (0.54 to 0.64)
40	1.01 (0.94 to 1.09)	0.61 (0.55 to 0.66)
41	1.04 (0.97 to 1.11)	0.55 (0.50 to 0.60)
42	0.92 (0.85 to 0.99)	0.58 (0.53 to 0.63)
43	0.89 (0.83 to 0.96)	0.57 (0.52 to 0.62)
44	0.87 (0.81 to 0.93)	0.55 (0.50 to 0.60)
45	0.83 (0.77 to 0.90)	0.55 (0.50 to 0.60)
46	0.83 (0.76 to 0.89)	0.54 (0.49 to 0.59)
47	0.78 (0.72 to 0.84)	0.59 (0.54 to 0.64)
48	0.78 (0.72 to 0.84)	0.52 (0.47 to 0.56)
49	0.78 (0.72 to 0.85)	0.56 (0.51 to 0.61)
50	0.75 (0.69 to 0.81)	0.48 (0.44 to 0.53)
51	0.73 (0.67 to 0.79)	0.53 (0.48 to 0.58)
52	0.71 (0.65 to 0.77)	0.50 (0.45 to 0.55)
53	0.59 (0.53 to 0.64)	0.54 (0.49 to 0.59)
54	0.57 (0.52 to 0.62)	0.45 (0.41 to 0.50)
55	0.59 (0.54 to 0.65)	0.48 (0.43 to 0.53)
56	0.51 (0.46 to 0.56)	0.45 (0.41 to 0.50)
57	0.49 (0.45 to 0.54)	0.45 (0.41 to 0.50)
58	0.45 (0.40 to 0.49)	0.44 (0.39 to 0.48)
59	0.50 (0.46 to 0.55)	0.42 (0.37 to 0.46)
60	0.43 (0.39 to 0.48)	0.37 (0.33 to 0.42)
61	0.41 (0.37 to 0.45)	0.40 (0.36 to 0.45)
62	0.37 (0.33 to 0.41)	0.31 (0.27 to 0.35)
63	0.42 (0.37 to 0.46)	0.34 (0.30 to 0.38)
64	0.32 (0.28 to 0.36)	0.31 (0.27 to 0.35)
65	0.33 (0.29 to 0.37)	0.26 (0.22 to 0.29)
66	0.33 (0.28 to 0.37)	0.26 (0.23 to 0.30)
67	0.30 (0.26 to 0.34)	0.26 (0.22 to 0.30)
68	0.32 (0.28 to 0.36)	0.25 (0.21 to 0.29)
69	0.32 (0.28 to 0.37)	0.25 (0.21 to 0.29)
70	0.32 (0.27 to 0.36)	0.22 (0.18 to 0.26)
71	0.33 (0.28 to 0.38)	0.26 (0.22 to 0.30)
72	0.34 (0.29 to 0.39)	0.25 (0.21 to 0.29)
73	0.34 (0.29 to 0.38)	0.21 (0.17 to 0.25)
74	0.34 (0.29 to 0.39)	0.22 (0.18 to 0.26)
75	0.29 (0.25 to 0.34)	0.23 (0.19 to 0.28)
76	0.28 (0.24 to 0.33)	0.21 (0.17 to 0.25)
77	0.26 (0.22 to 0.31)	0.20 (0.16 to 0.24)
78	0.34 (0.28 to 0.39)	0.19 (0.15 to 0.24)
79	0.30 (0.25 to 0.35)	0.18 (0.14 to 0.22)
80	0.24 (0.19 to 0.28)	0.17 (0.13 to 0.21)
81	0.29 (0.24 to 0.34)	0.18 (0.13 to 0.22)
82	0.28 (0.23 to 0.33)	0.21 (0.16 to 0.26)
83	0.29 (0.23 to 0.34)	0.21 (0.15 to 0.26)
84	0.25 (0.20 to 0.30)	0.17 (0.12 to 0.22)

85	0.28 (0.22 to 0.33)	0.21 (0.15 to 0.27)
86	0.22 (0.17 to 0.27)	0.17 (0.12 to 0.23)
87	0.21 (0.16 to 0.26)	0.13 (0.08 to 0.18)
88	0.20 (0.15 to 0.25)	0.17 (0.10 to 0.23)
89	0.16 (0.11 to 0.21)	0.16 (0.09 to 0.23)
90	0.12 (0.08 to 0.17)	0.17 (0.09 to 0.25)
91	0.14 (0.08 to 0.19)	0.18 (0.08 to 0.27)
92	0.14 (0.08 to 0.21)	0.06 (0.00 to 0.12)
93	0.14 (0.07 to 0.20)	0.19 (0.07 to 0.32)
94	0.16 (0.07 to 0.24)	0.02 (0.00 to 0.07)
95	0.03 (0.00 to 0.07)	0.07 (0.00 to 0.18)
96	0.03 (0.00 to 0.07)	/
97	0.11 (0.00 to 0.21)	/
98	0.07 (0.00 to 0.17)	/
99	/	/
100	/	/
Schizophrenia		
0	/	/
1	/	0.00 (0.00 to 0.00)
2	/	/
3	/	0.00 (0.00 to 0.01)
4	0.00 (0.00 to 0.00)	/
5	/	/
6	0.00 (0.00 to 0.00)	/
7	/	0.00 (0.00 to 0.00)
8	0.00 (0.00 to 0.00)	0.00 (0.00 to 0.01)
9	0.00 (0.00 to 0.00)	0.00 (0.00 to 0.00)
10	/	/
11	0.00 (0.00 to 0.01)	0.00 (0.00 to 0.01)
12	/	0.00 (0.00 to 0.00)
13	0.00 (0.00 to 0.00)	0.00 (0.00 to 0.01)
14	0.01 (0.00 to 0.02)	0.01 (0.00 to 0.01)
15	0.02 (0.01 to 0.03)	0.02 (0.01 to 0.03)
16	0.02 (0.01 to 0.03)	0.02 (0.01 to 0.03)
17	0.03 (0.02 to 0.04)	0.05 (0.03 to 0.06)
18	0.09 (0.07 to 0.10)	0.10 (0.08 to 0.12)
19	0.09 (0.07 to 0.11)	0.13 (0.10 to 0.15)
20	0.09 (0.07 to 0.11)	0.18 (0.16 to 0.21)
21	0.12 (0.09 to 0.14)	0.22 (0.19 to 0.26)
22	0.09 (0.07 to 0.12)	0.27 (0.24 to 0.31)
23	0.14 (0.11 to 0.17)	0.22 (0.19 to 0.26)
24	0.11 (0.09 to 0.14)	0.31 (0.27 to 0.35)
25	0.13 (0.11 to 0.16)	0.26 (0.23 to 0.30)
26	0.12 (0.09 to 0.14)	0.28 (0.25 to 0.32)
27	0.12 (0.09 to 0.14)	0.27 (0.24 to 0.31)
28	0.13 (0.10 to 0.15)	0.24 (0.21 to 0.28)
29	0.10 (0.08 to 0.12)	0.30 (0.26 to 0.33)
30	0.11 (0.09 to 0.13)	0.27 (0.23 to 0.31)

31	0.11 (0.09 to 0.14)	0.26 (0.23 to 0.30)
32	0.11 (0.09 to 0.14)	0.21 (0.18 to 0.25)
33	0.10 (0.08 to 0.12)	0.23 (0.19 to 0.26)
34	0.11 (0.08 to 0.13)	0.19 (0.16 to 0.23)
35	0.10 (0.08 to 0.13)	0.23 (0.20 to 0.26)
36	0.09 (0.07 to 0.11)	0.17 (0.14 to 0.20)
37	0.12 (0.10 to 0.15)	0.21 (0.18 to 0.24)
38	0.09 (0.07 to 0.11)	0.17 (0.14 to 0.20)
39	0.12 (0.09 to 0.14)	0.18 (0.15 to 0.21)
40	0.10 (0.08 to 0.12)	0.20 (0.17 to 0.23)
41	0.12 (0.10 to 0.15)	0.14 (0.11 to 0.17)
42	0.14 (0.11 to 0.16)	0.19 (0.16 to 0.22)
43	0.11 (0.09 to 0.13)	0.15 (0.13 to 0.18)
44	0.12 (0.10 to 0.15)	0.16 (0.13 to 0.18)
45	0.11 (0.09 to 0.14)	0.17 (0.14 to 0.20)
46	0.14 (0.12 to 0.17)	0.14 (0.12 to 0.17)
47	0.13 (0.10 to 0.15)	0.16 (0.14 to 0.19)
48	0.12 (0.10 to 0.14)	0.15 (0.12 to 0.17)
49	0.14 (0.11 to 0.17)	0.15 (0.12 to 0.17)
50	0.12 (0.10 to 0.14)	0.13 (0.11 to 0.16)
51	0.10 (0.08 to 0.13)	0.16 (0.13 to 0.19)
52	0.13 (0.10 to 0.15)	0.13 (0.10 to 0.15)
53	0.10 (0.08 to 0.12)	0.11 (0.09 to 0.14)
54	0.12 (0.09 to 0.14)	0.12 (0.09 to 0.14)
55	0.11 (0.09 to 0.13)	0.12 (0.10 to 0.15)
56	0.12 (0.09 to 0.14)	0.11 (0.09 to 0.14)
57	0.11 (0.08 to 0.13)	0.10 (0.08 to 0.12)
58	0.13 (0.10 to 0.15)	0.10 (0.08 to 0.12)
59	0.13 (0.11 to 0.15)	0.10 (0.08 to 0.13)
60	0.14 (0.12 to 0.17)	0.10 (0.08 to 0.12)
61	0.11 (0.09 to 0.13)	0.09 (0.07 to 0.12)
62	0.11 (0.08 to 0.13)	0.09 (0.07 to 0.11)
63	0.11 (0.08 to 0.13)	0.09 (0.07 to 0.11)
64	0.11 (0.09 to 0.14)	0.09 (0.07 to 0.11)
65	0.12 (0.10 to 0.15)	0.09 (0.06 to 0.11)
66	0.11 (0.09 to 0.13)	0.08 (0.06 to 0.10)
67	0.10 (0.08 to 0.13)	0.09 (0.07 to 0.11)
68	0.09 (0.07 to 0.12)	0.06 (0.04 to 0.08)
69	0.13 (0.11 to 0.16)	0.07 (0.05 to 0.09)
70	0.12 (0.09 to 0.14)	0.08 (0.06 to 0.11)
71	0.11 (0.08 to 0.13)	0.08 (0.06 to 0.10)
72	0.11 (0.08 to 0.14)	0.08 (0.06 to 0.11)
73	0.09 (0.07 to 0.12)	0.09 (0.06 to 0.11)
74	0.09 (0.07 to 0.12)	0.07 (0.05 to 0.10)
75	0.11 (0.08 to 0.13)	0.08 (0.06 to 0.11)
76	0.11 (0.08 to 0.14)	0.09 (0.06 to 0.12)
77	0.09 (0.06 to 0.12)	0.05 (0.03 to 0.07)
78	0.12 (0.09 to 0.15)	0.08 (0.05 to 0.11)

79	0.10 (0.07 to 0.13)	0.08 (0.05 to 0.11)
80	0.12 (0.09 to 0.15)	0.07 (0.04 to 0.10)
81	0.12 (0.09 to 0.16)	0.10 (0.07 to 0.14)
82	0.10 (0.07 to 0.13)	0.09 (0.05 to 0.12)
83	0.07 (0.04 to 0.09)	0.08 (0.05 to 0.12)
84	0.12 (0.09 to 0.16)	0.07 (0.04 to 0.10)
85	0.10 (0.06 to 0.13)	0.03 (0.01 to 0.05)
86	0.11 (0.07 to 0.14)	0.07 (0.04 to 0.11)
87	0.11 (0.07 to 0.15)	0.08 (0.04 to 0.12)
88	0.10 (0.07 to 0.14)	0.04 (0.01 to 0.08)
89	0.05 (0.02 to 0.08)	0.05 (0.01 to 0.08)
90	0.08 (0.04 to 0.12)	0.05 (0.01 to 0.10)
91	0.05 (0.02 to 0.08)	0.05 (0.00 to 0.09)
92	0.06 (0.02 to 0.10)	0.06 (0.00 to 0.12)
93	0.06 (0.01 to 0.10)	0.04 (0.00 to 0.11)
94	0.07 (0.02 to 0.13)	0.07 (0.00 to 0.16)
95	0.01 (0.00 to 0.04)	0.04 (0.00 to 0.12)
96	0.02 (0.00 to 0.06)	/
97	0.03 (0.00 to 0.08)	/
98	0.04 (0.00 to 0.11)	/
99	/	/
100	/	/

ADHD, attention deficit hyperactivity disorder. /, no case in this group.
Incidence rate was standardized by distribution of calendar period of the accumulated person-years during follow-up.

Table S4. Incidence rate differences of clinically diagnosed psychiatric disorders over the lifespan in females compared to males: a nationwide cohort study in Sweden, 2003-2019.

Age/Psychiatric disorders	Incidence rate differences per 1000 person-years
Any psychiatric disorder	
0-4	-1.45 (-1.52 to -1.38)
5-9	-8.93 (-9.08 to -8.79)
10-14	-1.53 (-1.69 to -1.37)
15-19	9.33 (9.12 to 9.54)
20-24	4.19 (4.00 to 4.38)
25-29	3.22 (3.05 to 3.39)
30-34	3.68 (3.53 to 3.84)
35-39	3.35 (3.21 to 3.49)
40-44	2.43 (2.30 to 2.57)
45-49	1.72 (1.59 to 1.85)
50-54	1.38 (1.25 to 1.51)
55-59	0.72 (0.60 to 0.84)
60-64	0.00 (-0.11 to 0.12)
65-69	-0.14 (-0.25 to -0.02)
70-74	0.28 (0.15 to 0.41)
75-79	1.06 (0.90 to 1.22)
80-84	1.81 (1.61 to 2.01)
85-89	1.95 (1.69 to 2.22)
90-94	1.29 (0.87 to 1.70)
95-99	1.48 (0.73 to 2.23)
Depressive disorders	
5-9	-0.08 (-0.09 to -0.06)
10-14	1.48 (1.39 to 1.58)
15-19	5.70 (5.53 to 5.87)
20-24	3.03 (2.87 to 3.20)
25-29	2.45 (2.29 to 2.61)
30-34	2.55 (2.40 to 2.70)
35-39	2.14 (2.00 to 2.27)
40-44	1.63 (1.50 to 1.75)
45-49	1.16 (1.03 to 1.28)
50-54	1.02 (0.90 to 1.14)
55-59	0.68 (0.57 to 0.80)
60-64	0.59 (0.48 to 0.69)
65-69	0.64 (0.53 to 0.74)
70-74	0.82 (0.70 to 0.95)
75-79	1.16 (1.00 to 1.32)
80-84	1.41 (1.21 to 1.62)
85-89	1.30 (1.02 to 1.58)
90-94	0.53 (0.11 to 0.96)
95-99	0.66 (-0.08 to 1.41)
Anxiety disorders	
5-9	0.01 (-0.01 to 0.03)
10-14	0.52 (0.46 to 0.57)

15-19	2.51 (2.40 to 2.61)
20-24	1.97 (1.86 to 2.09)
25-29	1.44 (1.33 to 1.55)
30-34	1.18 (1.08 to 1.28)
35-39	0.98 (0.89 to 1.06)
40-44	0.73 (0.65 to 0.81)
45-49	0.55 (0.48 to 0.62)
50-54	0.40 (0.34 to 0.47)
55-59	0.35 (0.29 to 0.40)
60-64	0.33 (0.28 to 0.38)
65-69	0.30 (0.25 to 0.34)
70-74	0.28 (0.23 to 0.33)
75-79	0.31 (0.26 to 0.37)
80-84	0.28 (0.22 to 0.34)
85-89	0.23 (0.15 to 0.30)
90-94	0.15 (0.05 to 0.26)
95-99	0.01 (-0.20 to 0.22)
Stress-related disorders	
0-4	-0.00 (-0.01 to 0.00)
5-9	-0.08 (-0.10 to -0.05)
10-14	0.43 (0.37 to 0.48)
15-19	2.32 (2.22 to 2.42)
20-24	2.23 (2.11 to 2.34)
25-29	2.09 (1.97 to 2.21)
30-34	2.36 (2.24 to 2.49)
35-39	2.24 (2.12 to 2.36)
40-44	2.14 (2.03 to 2.25)
45-49	1.82 (1.71 to 1.93)
50-54	1.45 (1.35 to 1.55)
55-59	0.97 (0.88 to 1.06)
60-64	0.60 (0.53 to 0.67)
65-69	0.28 (0.23 to 0.33)
70-74	0.22 (0.16 to 0.27)
75-79	0.17 (0.11 to 0.23)
80-84	0.11 (0.04 to 0.17)
85-89	0.03 (-0.05 to 0.11)
90-94	-0.14 (-0.26 to -0.03)
Eating disorders	
0-4	-0.02 (-0.03 to -0.00)
5-9	0.01 (-0.01 to 0.03)
10-14	1.52 (1.46 to 1.58)
15-19	3.71 (3.62 to 3.80)
20-24	2.22 (2.15 to 2.30)
25-29	1.36 (1.29 to 1.42)
30-34	0.80 (0.75 to 0.86)
35-39	0.44 (0.39 to 0.49)
40-44	0.18 (0.14 to 0.22)
45-49	0.09 (0.05 to 0.12)

50-54	0.07 (0.04 to 0.11)
55-59	0.03 (-0.00 to 0.07)
60-64	-0.14 (-0.17 to -0.11)
65-69	-0.18 (-0.21 to -0.15)
70-74	-0.17 (-0.20 to -0.14)
75-79	-0.11 (-0.14 to -0.09)
80-84	-0.03 (-0.05 to -0.00)
85-89	-0.01 (-0.04 to 0.02)
90-94	0.00 (-0.04 to 0.05)
ADHD	
0-4	-0.26 (-0.29 to -0.23)
5-9	-4.45 (-4.58 to -4.32)
10-14	-4.49 (-4.65 to -4.34)
15-19	0.18 (0.04 to 0.32)
20-24	0.12 (0.02 to 0.21)
25-29	-0.15 (-0.25 to -0.06)
30-34	-0.09 (-0.17 to -0.00)
35-39	-0.02 (-0.09 to 0.05)
40-44	-0.01 (-0.08 to 0.05)
45-49	-0.04 (-0.10 to 0.01)
50-54	-0.05 (-0.10 to -0.01)
55-59	-0.06 (-0.09 to -0.03)
60-64	-0.02 (-0.04 to -0.00)
65-69	-0.01 (-0.02 to 0.00)
70-74	-0.00 (-0.01 to 0.01)
Alcohol use disorders	
10-14	0.32 (0.27 to 0.38)
15-19	0.40 (0.28 to 0.52)
20-24	-0.50 (-0.62 to -0.39)
25-29	-0.89 (-0.98 to -0.80)
30-34	-0.94 (-1.02 to -0.86)
35-39	-0.92 (-1.00 to -0.85)
40-44	-0.93 (-1.01 to -0.84)
45-49	-1.13 (-1.22 to -1.04)
50-54	-1.30 (-1.39 to -1.21)
55-59	-1.47 (-1.55 to -1.38)
60-64	-1.65 (-1.73 to -1.56)
65-69	-1.61 (-1.70 to -1.52)
70-74	-1.35 (-1.44 to -1.26)
75-79	-1.11 (-1.20 to -1.03)
80-84	-0.78 (-0.87 to -0.69)
85-89	-0.49 (-0.58 to -0.40)
90-94	-0.30 (-0.41 to -0.19)
Autism spectrum disorders	
0-4	-0.93 (-0.98 to -0.87)
5-9	-1.92 (-2.00 to -1.84)
10-14	-1.66 (-1.76 to -1.57)
15-19	-0.54 (-0.63 to -0.45)

20-24	-0.41 (-0.47 to -0.34)
25-29	-0.29 (-0.35 to -0.23)
30-34	-0.19 (-0.24 to -0.14)
35-39	-0.10 (-0.14 to -0.06)
40-44	-0.07 (-0.11 to -0.04)
45-49	-0.08 (-0.11 to -0.05)
50-54	-0.07 (-0.10 to -0.05)
55-59	-0.06 (-0.08 to -0.03)
60-64	-0.03 (-0.05 to -0.01)
65-69	-0.02 (-0.04 to -0.01)
70-74	-0.01 (-0.03 to -0.00)
75-79	-0.01 (-0.02 to -0.00)
Drug use disorders	
0-4	-0.01 (-0.03 to -0.00)
5-9	0.00 (-0.01 to 0.01)
10-14	0.04 (0.01 to 0.07)
15-19	-0.71 (-0.80 to -0.62)
20-24	-1.49 (-1.59 to -1.39)
25-29	-1.22 (-1.31 to -1.14)
30-34	-0.60 (-0.67 to -0.53)
35-39	-0.28 (-0.34 to -0.23)
40-44	-0.11 (-0.17 to -0.05)
45-49	-0.10 (-0.16 to -0.05)
50-54	-0.09 (-0.14 to -0.04)
55-59	-0.03 (-0.08 to 0.01)
60-64	-0.03 (-0.07 to 0.01)
65-69	0.02 (-0.02 to 0.06)
70-74	0.05 (0.01 to 0.09)
75-79	0.08 (0.04 to 0.13)
80-84	0.10 (0.05 to 0.15)
85-89	0.04 (-0.03 to 0.11)
90-94	-0.02 (-0.14 to 0.09)
95-99	0.01 (-0.20 to 0.21)
Bipolar disorder	
5-9	-0.01 (-0.02 to -0.00)
10-14	0.03 (0.01 to 0.05)
15-19	0.62 (0.57 to 0.67)
20-24	0.94 (0.87 to 1.00)
25-29	0.72 (0.65 to 0.78)
30-34	0.59 (0.52 to 0.65)
35-39	0.49 (0.43 to 0.55)
40-44	0.38 (0.32 to 0.43)
45-49	0.25 (0.20 to 0.30)
50-54	0.17 (0.12 to 0.21)
55-59	0.06 (0.02 to 0.10)
60-64	0.04 (0.01 to 0.08)
65-69	0.06 (0.03 to 0.09)
70-74	0.09 (0.06 to 0.13)

75-79	0.09 (0.05 to 0.13)
80-84	0.08 (0.04 to 0.13)
85-89	0.05 (0.00 to 0.10)
90-94	0.00 (-0.07 to 0.07)
Schizophrenia	
15-19	-0.02 (-0.03 to -0.00)
20-24	-0.13 (-0.16 to -0.11)
25-29	-0.15 (-0.18 to -0.13)
30-34	-0.12 (-0.15 to -0.10)
35-39	-0.09 (-0.11 to -0.06)
40-44	-0.05 (-0.07 to -0.03)
45-49	-0.02 (-0.05 to -0.00)
50-54	-0.02 (-0.04 to 0.01)
55-59	0.01 (-0.01 to 0.03)
60-64	0.02 (0.00 to 0.04)
65-69	0.03 (0.01 to 0.05)
70-74	0.02 (0.00 to 0.04)
75-79	0.03 (0.00 to 0.05)
80-84	0.02 (-0.00 to 0.05)
85-89	0.04 (0.01 to 0.07)
90-94	0.01 (-0.03 to 0.06)

ADHD, attention deficit hyperactivity disorder.
The incidence rate differences were calculated for every 5-year age group and adjusted for age (as a continuous variable) and calendar year (grouped as every 4 years from 2003 and 2015-2019) at follow-up.
An incidence rate difference above 0 indicates a higher incidence rate among females whereas an incidence rate difference below 0 indicates a higher incidence rate among males.

Table S5. Incidence rate differences of psychiatric disorders over the lifespan in females compared to males, stratified analysis by calendar period: a nationwide cohort study in Sweden, 2003-2019

Age/psychiatric disorder	Incidence rate differences per 1000 person-years			
	2003-2006	2007-2010	2011-2014	2015-2019
Any psychiatric disorder				
0-4	-0.81 (-0.96 to -0.67)	-0.96 (-1.10 to -0.82)	-1.46 (-1.61 to -1.31)	-2.22 (-2.37 to -2.08)
5-9	-5.41 (-5.65 to -5.16)	-7.30 (-7.58 to -7.02)	-10.78 (-11.11 to -10.46)	-11.14 (-11.43 to -10.85)
10-14	-0.84 (-1.07 to -0.62)	-2.30 (-2.61 to -1.99)	-3.93 (-4.33 to -3.53)	0.17 (-0.21 to 0.55)
15-19	7.15 (6.82 to 7.49)	6.77 (6.39 to 7.15)	9.09 (8.61 to 9.57)	14.95 (14.45 to 15.45)
20-24	4.93 (4.58 to 5.28)	4.35 (3.97 to 4.74)	3.29 (2.91 to 3.68)	4.28 (3.90 to 4.65)
25-29	3.13 (2.82 to 3.43)	3.52 (3.16 to 3.88)	3.06 (2.69 to 3.44)	3.19 (2.86 to 3.51)
30-34	3.45 (3.18 to 3.72)	3.62 (3.30 to 3.94)	3.53 (3.18 to 3.89)	4.10 (3.78 to 4.43)
35-39	3.05 (2.79 to 3.31)	3.32 (3.04 to 3.61)	3.57 (3.25 to 3.88)	3.51 (3.21 to 3.82)
40-44	1.83 (1.56 to 2.10)	2.37 (2.10 to 2.63)	2.46 (2.18 to 2.74)	3.06 (2.79 to 3.33)
45-49	1.20 (0.94 to 1.46)	1.63 (1.35 to 1.90)	1.51 (1.24 to 1.77)	2.43 (2.18 to 2.67)
50-54	0.76 (0.51 to 1.01)	1.23 (0.96 to 1.50)	1.48 (1.21 to 1.76)	1.95 (1.72 to 2.18)
55-59	0.74 (0.51 to 0.96)	0.83 (0.58 to 1.08)	0.59 (0.33 to 0.86)	0.72 (0.49 to 0.95)
60-64	-0.20 (-0.43 to 0.03)	0.04 (-0.19 to 0.26)	0.13 (-0.11 to 0.37)	0.04 (-0.18 to 0.26)
65-69	0.09 (-0.17 to 0.34)	0.02 (-0.23 to 0.27)	0.10 (-0.13 to 0.33)	-0.59 (-0.79 to -0.39)
70-74	0.45 (0.16 to 0.73)	0.66 (0.36 to 0.96)	0.50 (0.22 to 0.79)	-0.17 (-0.38 to 0.03)
75-79	1.15 (0.84 to 1.46)	1.54 (1.19 to 1.90)	1.32 (0.97 to 1.68)	0.49 (0.21 to 0.76)
80-84	1.68 (1.31 to 2.05)	1.77 (1.36 to 2.19)	2.26 (1.82 to 2.69)	1.60 (1.24 to 1.96)
85-89	1.48 (0.97 to 1.99)	1.97 (1.44 to 2.50)	2.23 (1.65 to 2.81)	2.08 (1.59 to 2.57)
90-94	0.47 (-0.33 to 1.27)	1.49 (0.66 to 2.33)	1.51 (0.63 to 2.38)	1.47 (0.72 to 2.22)
95-99	1.02 (-0.32 to 2.36)	0.50 (-1.07 to 2.06)	2.22 (0.61 to 3.82)	1.82 (0.46 to 3.18)
Depressive disorders				
5-9	-0.06 (-0.09 to -0.02)	-0.07 (-0.10 to -0.03)	-0.09 (-0.13 to -0.04)	-0.09 (-0.12 to -0.05)
10-14	0.50 (0.39 to 0.61)	0.85 (0.69 to 1.01)	1.38 (1.16 to 1.60)	2.96 (2.73 to 3.19)
15-19	3.70 (3.45 to 3.95)	4.53 (4.23 to 4.83)	6.01 (5.63 to 6.40)	8.56 (8.14 to 8.98)
20-24	3.07 (2.77 to 3.37)	3.32 (2.98 to 3.65)	2.88 (2.54 to 3.21)	2.91 (2.58 to 3.24)
25-29	2.12 (1.85 to 2.40)	2.76 (2.42 to 3.10)	2.59 (2.24 to 2.94)	2.37 (2.08 to 2.67)
30-34	2.08 (1.83 to 2.33)	2.67 (2.36 to 2.98)	2.94 (2.60 to 3.28)	2.58 (2.28 to 2.88)
35-39	1.94 (1.70 to 2.18)	2.29 (2.01 to 2.57)	2.57 (2.27 to 2.87)	1.80 (1.53 to 2.08)
40-44	1.65 (1.40 to 1.90)	1.81 (1.54 to 2.07)	1.77 (1.50 to 2.03)	1.31 (1.07 to 1.54)
45-49	1.04 (0.80 to 1.29)	1.54 (1.26 to 1.81)	1.08 (0.84 to 1.33)	1.00 (0.80 to 1.21)
50-54	0.95 (0.71 to 1.18)	1.25 (0.98 to 1.51)	1.11 (0.85 to 1.36)	0.84 (0.64 to 1.03)
55-59	0.85 (0.64 to 1.06)	0.85 (0.60 to 1.09)	0.69 (0.45 to 0.93)	0.37 (0.18 to 0.57)
60-64	0.66 (0.45 to 0.88)	0.78 (0.57 to 0.99)	0.67 (0.45 to 0.88)	0.26 (0.08 to 0.43)
65-69	0.91 (0.66 to 1.16)	0.89 (0.66 to 1.13)	0.68 (0.48 to 0.87)	0.26 (0.10 to 0.42)
70-74	0.93 (0.64 to 1.23)	1.26 (0.95 to 1.56)	0.95 (0.69 to 1.21)	0.46 (0.28 to 0.63)
75-79	1.47 (1.12 to 1.81)	1.41 (1.04 to 1.79)	1.34 (0.98 to 1.70)	0.66 (0.41 to 0.91)
80-84	1.45 (1.03 to 1.87)	1.64 (1.19 to 2.09)	1.63 (1.18 to 2.09)	1.04 (0.70 to 1.39)
85-89	1.17 (0.58 to 1.77)	1.62 (1.03 to 2.21)	1.43 (0.82 to 2.05)	1.02 (0.54 to 1.50)
90-94	0.38 (-0.49 to 1.24)	0.69 (-0.23 to 1.60)	0.37 (-0.57 to 1.30)	0.66 (-0.06 to 1.38)
95-99	0.89 (-0.54 to 2.31)	0.40 (-1.19 to 1.99)	0.96 (-0.67 to 2.60)	0.50 (-0.78 to 1.78)

Anxiety disorders				
5-9	0.00 (-0.03 to 0.03)	0.04 (0.00 to 0.07)	-0.01 (-0.06 to 0.03)	0.01 (-0.03 to 0.05)
10-14	0.16 (0.09 to 0.22)	0.19 (0.11 to 0.27)	0.33 (0.20 to 0.46)	1.23 (1.09 to 1.38)
15-19	1.08 (0.94 to 1.23)	1.53 (1.36 to 1.70)	2.51 (2.28 to 2.74)	4.86 (4.60 to 5.13)
20-24	1.28 (1.08 to 1.48)	1.66 (1.44 to 1.89)	2.00 (1.76 to 2.24)	2.70 (2.46 to 2.93)
25-29	0.71 (0.53 to 0.90)	1.34 (1.11 to 1.58)	1.54 (1.29 to 1.79)	1.94 (1.73 to 2.15)
30-34	0.74 (0.58 to 0.91)	1.07 (0.86 to 1.29)	1.25 (1.02 to 1.48)	1.63 (1.42 to 1.84)
35-39	0.68 (0.52 to 0.83)	0.96 (0.78 to 1.14)	1.12 (0.92 to 1.31)	1.17 (0.98 to 1.35)
40-44	0.49 (0.34 to 0.65)	0.81 (0.65 to 0.98)	0.84 (0.67 to 1.01)	0.75 (0.60 to 0.90)
45-49	0.40 (0.26 to 0.54)	0.57 (0.41 to 0.74)	0.53 (0.38 to 0.68)	0.68 (0.55 to 0.81)
50-54	0.28 (0.15 to 0.40)	0.40 (0.25 to 0.55)	0.47 (0.32 to 0.62)	0.45 (0.34 to 0.57)
55-59	0.32 (0.21 to 0.42)	0.49 (0.36 to 0.62)	0.30 (0.17 to 0.43)	0.28 (0.17 to 0.39)
60-64	0.32 (0.23 to 0.42)	0.43 (0.32 to 0.53)	0.32 (0.21 to 0.44)	0.24 (0.15 to 0.34)
65-69	0.32 (0.22 to 0.42)	0.29 (0.19 to 0.39)	0.38 (0.29 to 0.48)	0.21 (0.13 to 0.29)
70-74	0.24 (0.14 to 0.34)	0.29 (0.19 to 0.40)	0.39 (0.29 to 0.50)	0.22 (0.15 to 0.30)
75-79	0.28 (0.18 to 0.37)	0.37 (0.25 to 0.49)	0.37 (0.25 to 0.50)	0.26 (0.16 to 0.35)
80-84	0.22 (0.12 to 0.33)	0.31 (0.19 to 0.43)	0.35 (0.21 to 0.49)	0.26 (0.14 to 0.37)
85-89	0.16 (0.03 to 0.30)	0.29 (0.14 to 0.44)	0.32 (0.16 to 0.48)	0.15 (-0.00 to 0.29)
90-94	0.08 (-0.10 to 0.25)	0.24 (0.04 to 0.44)	0.19 (-0.02 to 0.40)	0.12 (-0.09 to 0.32)
95-99	-0.01 (-0.27 to 0.26)	0.01 (-0.50 to 0.51)	0.13 (-0.25 to 0.51)	-0.06 (-0.45 to 0.34)
Stress-related disorders				
0-4	-0.00 (-0.02 to 0.01)	-0.00 (-0.02 to 0.02)	-0.01 (-0.02 to 0.01)	-0.00 (-0.02 to 0.01)
5-9	-0.02 (-0.05 to 0.02)	-0.07 (-0.11 to -0.02)	-0.12 (-0.17 to -0.06)	-0.10 (-0.14 to -0.05)
10-14	0.21 (0.13 to 0.28)	0.31 (0.21 to 0.42)	0.54 (0.40 to 0.68)	0.63 (0.51 to 0.75)
15-19	1.48 (1.32 to 1.64)	1.93 (1.75 to 2.11)	2.54 (2.32 to 2.77)	3.33 (3.10 to 3.56)
20-24	1.56 (1.35 to 1.76)	1.93 (1.70 to 2.16)	2.22 (1.99 to 2.45)	2.94 (2.71 to 3.18)
25-29	1.02 (0.83 to 1.22)	1.52 (1.29 to 1.75)	2.09 (1.83 to 2.34)	3.20 (2.96 to 3.44)
30-34	1.36 (1.17 to 1.55)	1.81 (1.58 to 2.05)	2.25 (1.99 to 2.52)	3.84 (3.56 to 4.12)
35-39	1.30 (1.11 to 1.48)	1.75 (1.53 to 1.96)	2.12 (1.87 to 2.37)	3.81 (3.52 to 4.09)
40-44	1.00 (0.81 to 1.19)	1.50 (1.29 to 1.70)	2.10 (1.86 to 2.34)	3.80 (3.53 to 4.06)
45-49	0.86 (0.67 to 1.04)	1.35 (1.14 to 1.55)	1.67 (1.46 to 1.89)	3.10 (2.87 to 3.34)
50-54	0.69 (0.53 to 0.86)	1.12 (0.93 to 1.31)	1.39 (1.17 to 1.60)	2.35 (2.14 to 2.56)
55-59	0.55 (0.42 to 0.69)	0.79 (0.62 to 0.96)	0.92 (0.73 to 1.11)	1.59 (1.39 to 1.78)
60-64	0.33 (0.21 to 0.45)	0.45 (0.32 to 0.57)	0.66 (0.52 to 0.81)	0.94 (0.78 to 1.10)
65-69	0.29 (0.18 to 0.40)	0.23 (0.12 to 0.34)	0.34 (0.23 to 0.45)	0.26 (0.16 to 0.35)
70-74	0.14 (0.03 to 0.25)	0.25 (0.14 to 0.37)	0.21 (0.10 to 0.32)	0.24 (0.15 to 0.33)
75-79	0.21 (0.10 to 0.31)	0.17 (0.05 to 0.30)	0.10 (-0.03 to 0.23)	0.18 (0.07 to 0.29)
80-84	0.07 (-0.05 to 0.18)	0.14 (0.01 to 0.27)	0.13 (-0.02 to 0.27)	0.09 (-0.03 to 0.22)
85-89	0.07 (-0.07 to 0.21)	0.05 (-0.09 to 0.20)	0.06 (-0.11 to 0.22)	-0.04 (-0.20 to 0.12)
90-94	-0.14 (-0.38 to 0.10)	-0.12 (-0.34 to 0.09)	-0.14 (-0.37 to 0.09)	-0.16 (-0.37 to 0.05)
Eating disorders				
0-4	-0.01 (-0.04 to 0.01)	-0.01 (-0.04 to 0.01)	0.01 (-0.02 to 0.03)	-0.05 (-0.09 to -0.02)
5-9	0.01 (-0.02 to 0.03)	-0.01 (-0.03 to 0.02)	0.05 (0.02 to 0.08)	-0.01 (-0.05 to 0.03)
10-14	0.76 (0.68 to 0.85)	1.12 (1.01 to 1.24)	1.62 (1.48 to 1.76)	2.46 (2.31 to 2.60)
15-19	2.84 (2.68 to 3.00)	3.35 (3.19 to 3.52)	4.03 (3.83 to 4.23)	4.62 (4.41 to 4.82)
20-24	2.27 (2.11 to 2.43)	2.42 (2.26 to 2.58)	2.09 (1.94 to 2.24)	2.16 (2.02 to 2.30)
25-29	1.39 (1.26 to 1.52)	1.40 (1.26 to 1.54)	1.22 (1.08 to 1.35)	1.40 (1.28 to 1.52)
30-34	0.78 (0.68 to 0.88)	0.84 (0.73 to 0.95)	0.79 (0.66 to 0.92)	0.81 (0.69 to 0.92)

35-39	0.45 (0.36 to 0.53)	0.48 (0.39 to 0.57)	0.44 (0.34 to 0.55)	0.39 (0.30 to 0.49)
40-44	0.15 (0.09 to 0.22)	0.20 (0.12 to 0.27)	0.21 (0.13 to 0.30)	0.15 (0.06 to 0.23)
45-49	0.10 (0.03 to 0.17)	0.07 (-0.01 to 0.15)	0.12 (0.04 to 0.19)	0.07 (-0.00 to 0.14)
50-54	0.08 (0.02 to 0.15)	0.06 (-0.02 to 0.14)	0.15 (0.07 to 0.24)	0.01 (-0.06 to 0.08)
55-59	0.03 (-0.02 to 0.09)	0.05 (-0.02 to 0.12)	0.11 (0.03 to 0.19)	-0.04 (-0.11 to 0.03)
60-64	-0.11 (-0.16 to -0.05)	-0.14 (-0.21 to -0.08)	-0.10 (-0.17 to -0.03)	-0.20 (-0.26 to -0.14)
65-69	-0.11 (-0.16 to -0.05)	-0.21 (-0.28 to -0.14)	-0.15 (-0.22 to -0.09)	-0.22 (-0.27 to -0.16)
70-74	-0.16 (-0.22 to -0.10)	-0.22 (-0.29 to -0.15)	-0.15 (-0.22 to -0.09)	-0.17 (-0.22 to -0.12)
75-79	-0.02 (-0.06 to 0.03)	-0.15 (-0.22 to -0.08)	-0.15 (-0.21 to -0.09)	-0.14 (-0.19 to -0.08)
80-84	0.04 (-0.01 to 0.09)	-0.05 (-0.11 to 0.01)	-0.04 (-0.09 to 0.01)	-0.06 (-0.11 to -0.01)
85-89	0.03 (-0.03 to 0.10)	-0.03 (-0.10 to 0.04)	-0.01 (-0.07 to 0.05)	-0.04 (-0.09 to 0.02)
90-94	0.01 (-0.11 to 0.13)	-0.01 (-0.10 to 0.08)	0.02 (-0.04 to 0.09)	-0.01 (-0.08 to 0.07)
ADHD				
0-4	-0.15 (-0.20 to -0.10)	-0.19 (-0.24 to -0.14)	-0.30 (-0.36 to -0.24)	-0.35 (-0.41 to -0.29)
5-9	-1.63 (-1.79 to -1.48)	-3.23 (-3.45 to -3.00)	-5.40 (-5.68 to -5.11)	-6.51 (-6.79 to -6.24)
10-14	-1.88 (-2.04 to -1.73)	-4.15 (-4.42 to -3.88)	-6.36 (-6.74 to -5.97)	-5.97 (-6.34 to -5.60)
15-19	-0.61 (-0.74 to -0.49)	-0.94 (-1.16 to -0.72)	-0.12 (-0.46 to 0.22)	2.49 (2.11 to 2.87)
20-24	-0.23 (-0.32 to -0.14)	-0.24 (-0.42 to -0.05)	0.05 (-0.17 to 0.27)	0.72 (0.49 to 0.95)
25-29	-0.20 (-0.28 to -0.13)	-0.43 (-0.61 to -0.25)	-0.29 (-0.51 to -0.07)	0.17 (-0.04 to 0.38)
30-34	-0.10 (-0.16 to -0.03)	-0.27 (-0.43 to -0.12)	-0.07 (-0.29 to 0.14)	0.07 (-0.14 to 0.28)
35-39	-0.05 (-0.11 to 0.00)	-0.11 (-0.25 to 0.02)	-0.02 (-0.20 to 0.16)	0.11 (-0.09 to 0.30)
40-44	-0.06 (-0.12 to -0.00)	-0.08 (-0.20 to 0.04)	-0.00 (-0.16 to 0.15)	0.09 (-0.07 to 0.25)
45-49	-0.06 (-0.11 to -0.02)	-0.13 (-0.25 to -0.02)	-0.09 (-0.21 to 0.04)	0.09 (-0.04 to 0.21)
50-54	-0.04 (-0.07 to -0.01)	-0.10 (-0.18 to -0.01)	-0.10 (-0.21 to 0.01)	0.00 (-0.10 to 0.10)
55-59	-0.01 (-0.02 to 0.01)	-0.06 (-0.11 to 0.00)	-0.07 (-0.16 to 0.01)	-0.10 (-0.18 to -0.02)
60-64	0.00 (-0.01 to 0.01)	-0.01 (-0.04 to 0.02)	-0.03 (-0.08 to 0.02)	-0.05 (-0.10 to 0.00)
65-69	0.00 (-0.01 to 0.01)	-0.01 (-0.04 to 0.01)	-0.01 (-0.04 to 0.02)	-0.01 (-0.03 to 0.02)
70-74	-0.00 (-0.01 to 0.01)	-0.01 (-0.02 to 0.00)	-0.01 (-0.03 to 0.01)	0.00 (-0.01 to 0.02)
Alcohol use disorders				
10-14	0.39 (0.28 to 0.49)	0.32 (0.19 to 0.45)	0.26 (0.16 to 0.36)	0.31 (0.23 to 0.40)
15-19	0.53 (0.29 to 0.77)	0.19 (-0.06 to 0.45)	0.35 (0.08 to 0.62)	0.53 (0.30 to 0.76)
20-24	-0.55 (-0.76 to -0.34)	-0.49 (-0.72 to -0.25)	-0.66 (-0.90 to -0.42)	-0.35 (-0.55 to -0.14)
25-29	-0.80 (-0.96 to -0.64)	-1.07 (-1.26 to -0.87)	-1.00 (-1.20 to -0.79)	-0.77 (-0.93 to -0.61)
30-34	-0.75 (-0.88 to -0.61)	-1.06 (-1.22 to -0.89)	-1.11 (-1.29 to -0.93)	-0.88 (-1.04 to -0.72)
35-39	-0.94 (-1.09 to -0.80)	-1.00 (-1.16 to -0.85)	-0.90 (-1.06 to -0.73)	-0.85 (-1.01 to -0.70)
40-44	-1.14 (-1.31 to -0.97)	-1.02 (-1.19 to -0.84)	-0.85 (-1.02 to -0.68)	-0.73 (-0.88 to -0.57)
45-49	-1.22 (-1.40 to -1.05)	-1.39 (-1.58 to -1.19)	-1.16 (-1.34 to -0.98)	-0.83 (-0.98 to -0.68)
50-54	-1.33 (-1.51 to -1.16)	-1.50 (-1.69 to -1.30)	-1.42 (-1.62 to -1.23)	-1.04 (-1.20 to -0.88)
55-59	-1.39 (-1.55 to -1.23)	-1.52 (-1.70 to -1.34)	-1.66 (-1.86 to -1.47)	-1.33 (-1.50 to -1.17)
60-64	-1.73 (-1.90 to -1.55)	-1.72 (-1.89 to -1.55)	-1.68 (-1.86 to -1.50)	-1.47 (-1.64 to -1.30)
65-69	-1.46 (-1.65 to -1.28)	-1.68 (-1.87 to -1.49)	-1.64 (-1.81 to -1.46)	-1.63 (-1.79 to -1.46)
70-74	-1.14 (-1.32 to -0.97)	-1.41 (-1.60 to -1.22)	-1.37 (-1.56 to -1.18)	-1.41 (-1.57 to -1.26)
75-79	-0.74 (-0.90 to -0.59)	-1.14 (-1.32 to -0.95)	-1.22 (-1.41 to -1.02)	-1.28 (-1.45 to -1.11)
80-84	-0.63 (-0.78 to -0.49)	-0.76 (-0.94 to -0.59)	-0.89 (-1.08 to -0.70)	-0.83 (-1.00 to -0.66)
85-89	-0.38 (-0.55 to -0.22)	-0.47 (-0.65 to -0.29)	-0.55 (-0.74 to -0.37)	-0.53 (-0.70 to -0.36)
90-94	-0.26 (-0.47 to -0.04)	-0.22 (-0.43 to 0.00)	-0.24 (-0.44 to -0.04)	-0.44 (-0.66 to -0.21)
Autism spectrum disorders				
0-4	-0.30 (-0.38 to -0.23)	-0.59 (-0.68 to -0.50)	-1.01 (-1.12 to -0.89)	-1.53 (-1.66 to -1.41)

5-9	-0.79 (-0.90 to -0.68)	-1.32 (-1.46 to -1.17)	-2.31 (-2.49 to -2.12)	-2.81 (-2.98 to -2.63)
10-14	-0.66 (-0.76 to -0.55)	-1.31 (-1.47 to -1.15)	-2.47 (-2.71 to -2.23)	-2.31 (-2.54 to -2.08)
15-19	-0.37 (-0.47 to -0.28)	-0.67 (-0.81 to -0.52)	-0.94 (-1.15 to -0.73)	-0.21 (-0.45 to 0.03)
20-24	-0.29 (-0.37 to -0.21)	-0.41 (-0.53 to -0.28)	-0.53 (-0.67 to -0.38)	-0.38 (-0.53 to -0.24)
25-29	-0.17 (-0.24 to -0.10)	-0.30 (-0.41 to -0.19)	-0.36 (-0.49 to -0.22)	-0.31 (-0.44 to -0.19)
30-34	-0.11 (-0.16 to -0.06)	-0.26 (-0.35 to -0.17)	-0.28 (-0.41 to -0.16)	-0.13 (-0.24 to -0.01)
35-39	-0.09 (-0.14 to -0.04)	-0.13 (-0.21 to -0.06)	-0.12 (-0.22 to -0.02)	-0.06 (-0.16 to 0.04)
40-44	-0.07 (-0.12 to -0.02)	-0.14 (-0.20 to -0.07)	-0.08 (-0.16 to 0.01)	-0.01 (-0.09 to 0.08)
45-49	-0.06 (-0.10 to -0.02)	-0.17 (-0.24 to -0.11)	-0.08 (-0.15 to -0.01)	-0.03 (-0.09 to 0.04)
50-54	-0.05 (-0.08 to -0.02)	-0.07 (-0.12 to -0.02)	-0.10 (-0.17 to -0.03)	-0.08 (-0.13 to -0.02)
55-59	-0.03 (-0.06 to -0.01)	-0.07 (-0.11 to -0.03)	-0.07 (-0.13 to -0.02)	-0.06 (-0.11 to -0.01)
60-64	-0.02 (-0.05 to -0.00)	-0.02 (-0.05 to 0.01)	-0.05 (-0.09 to -0.01)	-0.04 (-0.07 to 0.00)
65-69	-0.01 (-0.03 to 0.01)	-0.02 (-0.05 to -0.00)	-0.03 (-0.06 to -0.00)	-0.02 (-0.05 to 0.00)
70-74	-0.01 (-0.03 to 0.01)	-0.01 (-0.03 to 0.01)	-0.02 (-0.04 to 0.01)	-0.02 (-0.04 to 0.00)
75-79	0.00 (-0.01 to 0.02)	-0.01 (-0.03 to 0.01)	-0.03 (-0.05 to 0.00)	-0.02 (-0.04 to 0.00)
Drug use disorders				
0-4	-0.05 (-0.09 to -0.01)	-0.00 (-0.03 to 0.02)	-0.01 (-0.02 to 0.01)	-0.00 (-0.02 to 0.01)
5-9	0.02 (-0.00 to 0.03)	-0.00 (-0.02 to 0.01)	-0.01 (-0.02 to 0.00)	0.00 (-0.01 to 0.01)
10-14	0.07 (0.02 to 0.11)	0.04 (-0.01 to 0.10)	0.02 (-0.05 to 0.09)	0.04 (-0.03 to 0.10)
15-19	0.21 (0.07 to 0.35)	-0.55 (-0.72 to -0.39)	-1.42 (-1.64 to -1.21)	-1.09 (-1.30 to -0.87)
20-24	-0.98 (-1.16 to -0.79)	-1.25 (-1.45 to -1.04)	-1.84 (-2.05 to -1.63)	-1.75 (-1.96 to -1.53)
25-29	-0.81 (-0.95 to -0.67)	-1.11 (-1.29 to -0.94)	-1.36 (-1.55 to -1.18)	-1.48 (-1.65 to -1.31)
30-34	-0.30 (-0.41 to -0.19)	-0.64 (-0.78 to -0.51)	-0.69 (-0.84 to -0.53)	-0.78 (-0.94 to -0.63)
35-39	-0.19 (-0.29 to -0.08)	-0.27 (-0.38 to -0.15)	-0.32 (-0.45 to -0.20)	-0.37 (-0.50 to -0.24)
40-44	-0.18 (-0.30 to -0.07)	-0.13 (-0.24 to -0.02)	-0.12 (-0.24 to -0.01)	-0.01 (-0.12 to 0.10)
45-49	-0.19 (-0.30 to -0.09)	-0.20 (-0.31 to -0.09)	-0.11 (-0.22 to -0.00)	0.05 (-0.05 to 0.15)
50-54	-0.06 (-0.15 to 0.03)	-0.15 (-0.25 to -0.05)	-0.15 (-0.26 to -0.04)	-0.04 (-0.13 to 0.06)
55-59	0.05 (-0.02 to 0.13)	-0.06 (-0.15 to 0.03)	-0.08 (-0.18 to 0.02)	-0.05 (-0.14 to 0.04)
60-64	0.04 (-0.03 to 0.11)	-0.00 (-0.08 to 0.07)	-0.02 (-0.11 to 0.06)	-0.12 (-0.20 to -0.03)
65-69	0.02 (-0.06 to 0.10)	0.05 (-0.03 to 0.12)	0.01 (-0.07 to 0.09)	0.01 (-0.06 to 0.09)
70-74	0.04 (-0.04 to 0.12)	0.04 (-0.05 to 0.12)	0.06 (-0.03 to 0.14)	0.06 (-0.01 to 0.13)
75-79	0.02 (-0.06 to 0.10)	0.08 (-0.00 to 0.17)	0.12 (0.02 to 0.22)	0.10 (0.02 to 0.19)
80-84	0.06 (-0.02 to 0.14)	0.12 (0.03 to 0.22)	0.07 (-0.05 to 0.18)	0.15 (0.04 to 0.25)
85-89	-0.00 (-0.13 to 0.12)	0.06 (-0.06 to 0.18)	0.05 (-0.10 to 0.20)	0.04 (-0.11 to 0.19)
90-94	-0.04 (-0.24 to 0.17)	0.05 (-0.14 to 0.23)	0.03 (-0.16 to 0.22)	-0.11 (-0.35 to 0.14)
95-99	0.02 (-0.25 to 0.29)	-0.33 (-0.88 to 0.21)	0.15 (-0.29 to 0.58)	0.11 (-0.24 to 0.46)
Bipolar disorder				
5-9	-0.00 (-0.02 to 0.01)	-0.02 (-0.04 to -0.00)	-0.00 (-0.02 to 0.02)	-0.01 (-0.02 to 0.00)
10-14	0.00 (-0.02 to 0.02)	0.02 (-0.02 to 0.07)	0.02 (-0.02 to 0.07)	0.05 (0.02 to 0.09)
15-19	0.17 (0.11 to 0.23)	0.64 (0.55 to 0.74)	0.72 (0.61 to 0.83)	0.92 (0.81 to 1.03)
20-24	0.32 (0.23 to 0.41)	0.95 (0.81 to 1.09)	1.09 (0.95 to 1.23)	1.24 (1.10 to 1.38)
25-29	0.28 (0.19 to 0.38)	0.77 (0.62 to 0.92)	0.90 (0.75 to 1.06)	0.85 (0.72 to 0.98)
30-34	0.26 (0.18 to 0.35)	0.60 (0.47 to 0.73)	0.70 (0.55 to 0.85)	0.79 (0.65 to 0.92)
35-39	0.24 (0.15 to 0.32)	0.65 (0.53 to 0.78)	0.64 (0.51 to 0.78)	0.46 (0.33 to 0.58)
40-44	0.15 (0.06 to 0.24)	0.46 (0.35 to 0.58)	0.46 (0.35 to 0.58)	0.41 (0.31 to 0.52)
45-49	0.17 (0.08 to 0.26)	0.28 (0.16 to 0.40)	0.29 (0.18 to 0.40)	0.25 (0.16 to 0.34)
50-54	0.07 (-0.02 to 0.16)	0.21 (0.10 to 0.33)	0.15 (0.05 to 0.25)	0.21 (0.14 to 0.29)
55-59	0.02 (-0.06 to 0.10)	0.11 (0.01 to 0.21)	0.11 (0.01 to 0.20)	0.02 (-0.05 to 0.10)

60-64	0.02 (-0.06 to 0.11)	0.11 (0.03 to 0.19)	0.03 (-0.05 to 0.10)	0.01 (-0.05 to 0.07)
65-69	0.09 (0.00 to 0.18)	0.08 (-0.01 to 0.16)	0.07 (0.00 to 0.13)	0.02 (-0.03 to 0.07)
70-74	0.12 (0.02 to 0.22)	0.14 (0.05 to 0.22)	0.10 (0.03 to 0.18)	0.05 (0.00 to 0.10)
75-79	0.11 (0.02 to 0.20)	0.11 (0.01 to 0.21)	0.09 (0.01 to 0.17)	0.06 (0.01 to 0.12)
80-84	0.09 (0.00 to 0.18)	0.10 (-0.00 to 0.21)	0.10 (0.01 to 0.19)	0.05 (-0.02 to 0.11)
85-89	0.10 (-0.02 to 0.21)	0.04 (-0.08 to 0.15)	0.04 (-0.07 to 0.15)	0.04 (-0.04 to 0.12)
90-94	-0.01 (-0.16 to 0.14)	-0.01 (-0.17 to 0.16)	0.03 (-0.11 to 0.17)	-0.01 (-0.11 to 0.09)
Schizophrenia				
15-19	-0.02 (-0.05 to 0.01)	-0.01 (-0.04 to 0.02)	-0.03 (-0.06 to -0.00)	-0.01 (-0.03 to 0.02)
20-24	-0.16 (-0.22 to -0.10)	-0.11 (-0.17 to -0.06)	-0.11 (-0.16 to -0.07)	-0.14 (-0.19 to -0.10)
25-29	-0.13 (-0.19 to -0.07)	-0.16 (-0.22 to -0.09)	-0.14 (-0.19 to -0.08)	-0.18 (-0.22 to -0.13)
30-34	-0.11 (-0.17 to -0.06)	-0.14 (-0.19 to -0.08)	-0.12 (-0.18 to -0.07)	-0.13 (-0.17 to -0.08)
35-39	-0.12 (-0.18 to -0.07)	-0.09 (-0.14 to -0.03)	-0.06 (-0.10 to -0.01)	-0.08 (-0.12 to -0.04)
40-44	-0.07 (-0.13 to -0.01)	-0.06 (-0.11 to -0.00)	-0.05 (-0.09 to -0.00)	-0.04 (-0.07 to -0.00)
45-49	-0.04 (-0.10 to 0.02)	-0.05 (-0.11 to 0.00)	0.00 (-0.04 to 0.04)	-0.01 (-0.04 to 0.02)
50-54	-0.02 (-0.07 to 0.04)	-0.05 (-0.10 to 0.00)	0.01 (-0.04 to 0.05)	-0.01 (-0.04 to 0.02)
55-59	0.01 (-0.04 to 0.06)	-0.00 (-0.05 to 0.04)	0.04 (0.00 to 0.09)	-0.01 (-0.04 to 0.02)
60-64	0.03 (-0.02 to 0.09)	0.02 (-0.03 to 0.06)	0.02 (-0.01 to 0.06)	0.01 (-0.01 to 0.04)
65-69	0.06 (0.00 to 0.12)	0.04 (-0.00 to 0.09)	0.02 (-0.01 to 0.05)	0.02 (-0.01 to 0.05)
70-74	-0.01 (-0.07 to 0.06)	0.06 (0.00 to 0.11)	0.02 (-0.02 to 0.06)	0.02 (-0.01 to 0.04)
75-79	0.01 (-0.04 to 0.07)	0.05 (-0.01 to 0.11)	0.01 (-0.04 to 0.06)	0.03 (-0.00 to 0.07)
80-84	0.05 (-0.01 to 0.10)	0.04 (-0.03 to 0.11)	0.02 (-0.04 to 0.08)	0.00 (-0.04 to 0.04)
85-89	0.04 (-0.03 to 0.12)	0.06 (-0.01 to 0.12)	0.05 (-0.01 to 0.12)	0.02 (-0.02 to 0.07)
90-94	0.01 (-0.08 to 0.11)	0.03 (-0.07 to 0.13)	0.01 (-0.08 to 0.10)	-0.00 (-0.08 to 0.07)

ADHD, attention deficit hyperactivity disorder.
The incidence rate differences were calculated for every 5-year age group and adjusted for age (as a continuous variable) at follow-up.
An incidence rate difference above 0 indicates a higher incidence rate among females whereas an incidence rate difference below 0 indicates a higher incidence rate among males.
